# Proteomic Analysis of Pig (*Sus scrofa*) Olfactory Soluble Proteome Reveals *O*-Linked-N-Acetylglucosaminylation of Secreted Odorant-Binding Proteins

**DOI:** 10.3389/fendo.2014.00202

**Published:** 2014-12-05

**Authors:** Patricia Nagnan-Le Meillour, Anne-Sophie Vercoutter-Edouart, Frédérique Hilliou, Chrystelle Le Danvic, Frédéric Lévy

**Affiliations:** ^1^UMR 8576, USC-Unité de Glycobiologie Structurale et Fonctionnelle, INRA, CNRS, Université de Lille 1, Villeneuve d’Ascq, France; ^2^UMR 8576, Unité de Glycobiologie Structurale et Fonctionnelle, CNRS, Université de Lille 1, Villeneuve d’Ascq, France; ^3^UMR 7254, UMR 1355 Institut Sophia Agrobiotech, INRA, CNRS, Université de Nice Sophia Antipolis, Sophia Antipolis, France; ^4^Unité de Glycobiologie Structurale et Fonctionnelle, Union Nationale des Coopératives Agricoles d’Elevage et d’Insémination Animale (UNCEIA), Villeneuve d’Ascq, France; ^5^UMR 7247, UMR 85 Unité de Physiologie de la Reproduction et des Comportements, INRA, CNRS, Université François Rabelais, Haras Nationaux, Nouzilly, France

**Keywords:** olfaction, odorant-binding protein, extracellular *O*-linked-N-acetylglucosaminylation, olfactory secretome, *O*-GlcNAc transferase

## Abstract

The diversity of olfactory binding proteins (OBPs) is a key point to understand their role in molecular olfaction. Since only few different sequences were characterized in each mammalian species, they have been considered as passive carriers of odors and pheromones. We have explored the soluble proteome of pig nasal mucus, taking benefit of the powerful tools of proteomics. Combining two-dimensional electrophoresis, mass spectrometry, and western-blot with specific antibodies, our analyses revealed for the first time that the pig nasal mucus is mainly composed of secreted OBP isoforms, some of them being potentially modified by *O*-GlcNAcylation. An ortholog gene of the glycosyltransferase responsible of the *O*-GlcNAc linking on extracellular proteins in *Drosophila* and Mouse (EOGT) was amplified from tissues of pigs of different ages and sex. The sequence was used in a phylogenetic analysis, which evidenced conservation of EOGT in insect and mammalian models studied in molecular olfaction. Extracellular *O*-GlcNAcylation of secreted OBPs could finely modulate their binding specificities to odors and pheromones. This constitutes a new mechanism for extracellular signaling by OBPs, suggesting that they act as the first step of odor discrimination.

## Introduction

Olfaction is generally considered as a minor, primitive sense for human beings, and the study of this sense in mammals has been neglected. However, many odor-guided behaviors are involved in the establishment of biological functions: reproduction *via* the choice of mate partner, establishment of the mother–young bond, maintenance of the social hierarchy, and to a less extent choice of food. This dialog between partners of the same species is driven by pheromones. The sex pheromone of pig is one of the few characterized in mammals: a mixture of androstenol and androstenone (1) secreted in testis is transported by lipocalins in blood to the saliva. During sex behavior, the male produces high quantity of saliva that, when perceived by the female, evokes a typical posture called lordosis, meaning the male acceptation by the female (2). Besides the identification of pheromones, studies have focused for the two past decades on the molecular and cellular mechanisms involved in pheromone reception, starting with the discovery of a gene family encoding odorant receptors (3). A general scheme of olfactory coding hypothesized that pheromones are detected by sensory neurons of the vomeronasal organ (VNO), while other odors are detected by the main olfactory epithelium (MOE) sensory neurons [reviewed in Ref. (4, 5)]. There is a large body of evidence that the coding of olfactory signals is more complex. Some pheromone-mediated behaviors are still effective after VNO lesions (6–8). Conversely, mouse VNO neurons can be stimulated by odorants emitted by other species, such as floral and woody smelling compounds (9).

The reception of olfactory signals takes place in the nasal mucus. The biochemical players are olfactory receptors (ORs), olfactory binding proteins (OBPs), and odorant degrading enzymes, whose kinetic interactions are not fully understood. Among them, OBPs are the best characterized. They are small water-soluble proteins secreted in high quantity in the nasal mucus by Bowman’s gland of the olfactory epithelium (10, 11). One major unresolved question in mammalian olfaction is the nature of the ligand of ORs. Two hypotheses have been proposed: (1) the ligand is the odorant molecule itself solubilized and transported to the receptor by OBPs. In this scheme, the binding between odorant molecules and OBPs is unspecific, which is supported by the small number of OBP genes in each animal species [reviewed in Ref. (12)]. OBPs are also assumed to concentrate odors and/or to scavenge them from receptors in a deactivation process (13). (2) The ligand is the complex formed by the specific binding between a given odorant molecule and a specific OBP. This hypothesis involves a conformational change of the protein upon ligand binding, which confers an “activated” form to the complex, able to interact with a specific OR. Recently, it was shown that the complexes are internalized by the olfactory epithelium after activation of the receptors (14), supporting the hypothesis that OBP/odor complexes are the ligand of OR. Contrary to insects, where c. a. 30 OBP genes were identified in olfactory tissues (15, 16), no more than 3–4 OBP genes have been characterized in pig, rat, and human (17–19). As the few number of OBPs limits the possibility of a key-role in the coding of pheromones and odors, they have been considered as passive carriers in mammals (20).

However, the possibility of OBP diversity at the protein level has been evoked since the time of their discovery (17, 21–23). Recently, Stopkova et al. (24) identified eight OBP genes in mouse genome, suggesting a larger OBP diversity than previously described. In pig, we have demonstrated that post-translational modifications (PTM) generate OBP isoforms with specific binding properties, reinforcing the possibility of an active role of mammalian OBPs in pheromone and odor coding. Thus, we have demonstrated that two OBPs in pig, the OBP (*stricto sensu*) and the VEG (Von Ebner’s Gland protein), can undergo phosphorylation, which generates several isoforms for each corresponding primary sequence (25). Moreover, we have shown that the binding specificity for pheromones is driven by phosphorylation (26) and/or *O*-GlcNAcylation for two VEG isoforms (27). This diversity of isoforms with different binding abilities rekindles the debate on the OBPs role in pheromone coding in the pig, and in mammals in general. Previous studies were performed in one-dimensional electrophoresis and did not allow a precise characterization of the pig OBP diversity. To go further in the characterization of both OBP isoforms and their PTM, in particular the *O*-GlcNAcylation unexpected for secreted proteins, we have explored the soluble proteome of pig nasal mucus, taking benefit of the powerful tools of proteomics. Combining two-dimensional electrophoresis (2-DE), mass spectrometry, and western-blot with specific antibodies, our analyses revealed for the first time that the pig nasal mucus is mainly composed of OBP isoforms, some of them being potentially modified by *O*-GlcNAcylation. As a glycosyltransferase (GT) responsible for *O*-GlcNAcylation of extracellular domains has been reported in *Drosophila* (28), we have searched for such a GT in the pig olfactory tissues. The encoding cDNA was cloned and the obtained sequence was used in a phylogenetic analysis to determine whether this modification could eventually occur in other model species used for the study of olfaction mechanisms.

## Materials and Methods

### Animals and tissues

Animals (Large White *Sus scrofa*) were maintained at the Experimental Farm of INRA (UEPAO, Nouzilly, France). Individuals coming from the same offspring (brothers and sisters) were slaughtered in agreement with EU directives about animal welfare. Pigs were sacrificed by a licensed butcher in an official slaughterhouse (authorization No. A37801 E37-175-2 agreement UEPAO). Four animals of different physiological status were used in this study: a pre-pubertal male, a pubertal male, a pre-pubertal female, and a pubertal female. Respiratory mucosa (RM) and VNO were dissected immediately after death from each anesthetized animal and stored half in tubes at −80°C for protein extraction, and half in RNA*later* RNA Stabilization Reagent for RNA extraction (Qiagen).

### Protein extraction

The proteins were extracted from pig frozen tissues by phase partition using chloroform/methanol (v:v, 2:1) on ice. The resulting samples were centrifuged (15,000 *g* for 15 min at 4°C) and the methanol phase was collected then evaporated in a Speed-vac concentrator. Aliquots were tested by native-polyacrylamide gel electrophoresis as already described (29) in order to obtain a standard quantity of proteins for each tissue (1X corresponds to 1 microg/band). The relative quantification of protein bands was calculated by using Image J software. Dried samples were stored at −20°C.

### Two-dimensional electrophoresis

All chemicals and reagents were from Sigma-Aldrich. Dried proteins (100 μg) were solubilized in 150 μl of rehydration buffer [8 M Urea, 2 M Thiourea, 2% (w/v) CHAPS, 10 mM dithiothreitol (DTT), 1.2% (v/v) Immobilized pH Gradient (IPG) buffer (pH 3–5.6) (GE Healthcare), and bromophenol blue]. After vigorous shaking, proteins were loaded onto 7-cm IPG strip (pH 3–5.6, GE Healthcare) by overnight passive rehydration at room temperature. The first-dimensional isoelectric focusing (IEF) was carried out on a Protean IEF Cell (Bio-Rad) using the following program: 300 V for 30 min (rapid voltage ramping), 1000 V for 1 h (rapid voltage ramping), 5000 V for 2 h (rapid voltage ramping), and 500 V for 3 h (rapid voltage ramping), with a current limit at 50 μA/gel. When IEF was complete (10,000 VH final), strips were incubated twice for 15 min in the equilibration buffer [375 mM Tris-HCL pH 8.8, 6 M urea, 2% (w/v) SDS, and 30% (v/v) glycerol] containing 1.5% (w/v) DTT then 2% (w/v) iodoacetamide. The second dimension separation was performed using 16.8% SDS–PAGE in Mini PROTEAN Tetra Cell (Bio-Rad).

### Staining and western-blot after 2-DE

After 2-D electrophoresis, gels were either stained with colloidal Coomassie blue R solution (12% trichloroacetic acid, 5% ethanolic solution of 0.035% Serva blue R 250), or transferred onto nitrocellulose (Hybond C-Extra, GE Healthcare) or PVDF (Immobilon P, Millipore) membranes. For immunodetection, membranes were blocked in 5% (w/v) non-fat dry milk in Tris-Buffered Saline-0.05% Tween-20 (TBS-T) for probing with polyclonal antibodies (anti-OBP, anti-SAL, and anti-VEG) and 3% Bovine Serum Albumin (BSA, Sigma-Aldrich) in TBS-T for probing with monoclonal anti-*O*-GlcNAc antibodies. Membranes were then incubated with antibodies in TBS-T 1 h at room temperature [RL2 (Thermofisher) 1:2,000; CTD 110.6 (Thermofisher), 1:2,000; anti-OBP, 1:30,000; anti-VEG, 1:5,000; and anti-SAL, 1:10,000]. After three washes in TBS-T, membranes were incubated with the appropriate horseradish peroxidase-conjugated secondary antibody (anti-mouse IgG-HRP linked, 1:30,000, Thermofisher, for RL2; anti-mouse IgM-HRP linked, 1:30,000, Thermofisher, for CTD 110.6; and anti-rabbit IgG-HRP linked, 1:30,000, Thermofisher for polyclonal antibodies) for 1 h at room temperature. After three washes in TBS-T, blots were developed using enhanced chemiluminescence (ECL Plus and ECL Prime Reagents, Hyperfilm™ MP, GE Healthcare). Coomassie blue stained gels and membranes were scanned on GS800 calibrated imaging Densitometer including the Quantity One^®^ software for image acquisition and analysis (Bio-Rad, Marnes-La-Coquette). Images were merged using Image J^®^ software.

### Mass spectrometry analysis

The spots of interest were excised from the gels, destained and digested by Trypsin Gold Mass Spectrometry grade (Promega) overnight at 37°C as previously described (26). Protein identification was performed either by MALDI-TOF MS or by Nano-LC-ESI-MS/MS. MALDI-TOF MS analysis was performed on an Applied Biosystem, Voyager DE Pro MALDI-TOF. The instrument was used in reflector mode, measuring peptide masses on a range of 500–4,000 Da. Calibration points were based on the masses of the matrix cluster or trypsin autolysis products (m/z 842, m/z 2211). Protein identification was performed by comparison of measured peptide masses with the theoretical mass fingerprint of porcine OBP (GenBank accession number NP_998961), VEG (S77587), and SAL (P81608)^1^ obtained with the following parameters: trypsin digestion with two missed cleavages allowed, cysteines in reduced form, acrylamide adducts, methionine oxidized (MSO), and monoisotopic peptide masses as [M + H]^+^. Nano-LC-ESI-MS/MS was carried on a hybrid quadrupole time-of-flight mass spectrometer (Q-Star, Applied Biosystems) equipped with a nano-electrospray ion source coupled with a nano HPLC system (LC Packings Dionex). Database searching was performed using Mascot software (MS/MS ion search module) in the NCBInr database. Search parameters were as follow: other mammalians as taxonomy, 50 ppm tolerance for the parent ion mass and 50 amu for the MS/MS fragment ions, one missed cleavage allowed, carbamidomethylation of cysteine and methionine oxidation as possible modifications. Only candidate proteins with a significant Mascot score were taken into consideration (significance threshold for candidate <0.05 using MudPIT scoring method and an expectation value for ion peptides <0.05).

### Search for putative OBP and VEG splice variants by rapid amplication of cDNA ends-polymerase chain reaction and *in silico* analysis

Total RNA was extracted from 30 mg of each tissue conserved in RNA*later* with RNeasy Fibrous tissue Mini Kit according to manufacturer’s instructions (Qiagen, Courtaboeuf). Rapid amplication of cDNA ends-polymerase chain reaction (RACE-PCR) was performed with the GeneRacer™ kit according to manufacturer’s instructions (Invitrogen). Capped mRNAs (for 5′ RACE) and native mRNAs (for 3′ RACE) were reverse transcribed into cDNA with the Sensiscript III RT kit (Invitrogen) and oligo dT primer. Amplification of cDNA coding for *OBP* and *VEG* putative variants was performed with 1 μl of each RT product, Platinum^®^ Taq DNA polymerase High Fidelity (Invitrogen), and the set of primers described in Table 1, by touchdown PCR, as follows: 94°C for 2 min, followed by 5 cycles of 30 s at 94°C, 2 min at 72°C, 5 cycles of 30 s at 94°C, 2 min at 70°C, 25 cycles of 30 s at 94°C, 30 s at 65°C, 2 min at 68°C, and a final elongation of 10 min at 68°C. The PCR products were cloned into *pCR4*^®^*-TOPO* vector (Invitrogen). The recombinant plasmids were amplified into *Escherichia coli* One Shot Top 10 Chemically Competent cells (Invitrogen), then purified with the QIAprep Spin Miniprep kit (Qiagen, Courtaboeuf), and sequenced in both senses (GATC Biotech). In parallel, splicing variants were searched in the pig genome (Ensembl *Sus scrofa*) by performing Blastn analysis with mRNA *OBP*, *VEG*, and *SAL* sequences (NCBI Ref Seq: NM_213796, NM_213856, NM_213814, respectively).

**Table 1:** **Primers used in the RACE-PCR experiments for amplification of putative OBP and VEG splice variants**.

	5′RACE		3′RACE	
	5′Primer	3’ Primer	5′Primer	3′Primer
*OBP*	GeneRacer™ 5′primer	3′OBP primer: TCACTTGGCAGGACAGTCATCTCT	5′OBP primer: CACCCAGGAACCTCAACCTGAACA	GeneRacer™ 3′primer
*VEG*	GeneRacer™ 5′primer	3′VEG primer: CTAGTTCCCTCCTGGAGAGCAGGTTTCGCTTT	5′VEG primer: CAGGAGTTCCCGGCCGTGGGGCA	GeneRacer™ 3′primer

*The sequence of the GeneRacer primers is available at www.invitrogen.com. Gene specific 5′ primers correspond to the first codons coding for the N-terminal of protein sequence (without signal peptide) and gene specific 3′ primers to the last codons of the C-terminal of the protein sequence*.

### Amplification and molecular cloning of *Sus scrofa EOGT*

Messenger RNA obtained by total RNA extraction (see above) were reverse transcribed into cDNA with the Sensiscript III RT kit (Invitrogen) using a 3′-end gene specific primer (5′-TCACTGGGATCCCCGAGCTCATCATGTTTGTTCTT-3′) designed from the transcript ID ENSSSCT00000012589 (Ensembl database, *Sus scrofa* genome). PCR amplification of *EOGT* in each tissue was carried out on a thermal cycler (Bio-Rad) with 1 μl of RT product as template with Platinum^®^ Taq DNA polymerase High Fidelity (Invitrogen), with primers (Sense 5′-ATGTTAATGTTGCTTGTCTTTGGAGCATTGCTT-3′ and antisense 5′-TTAGAGCTCATCATGTTTGTTCTTAAAAGGCCACTT-3′), according to manufacturer’s instructions. Touchdown PCR was performed as described above. The single product obtained by amplification from adult male RM was cloned into *pCR*^®^ *4-TOPO* vector (Invitrogen). The recombinant plasmid was amplified and sequenced as described above.

### Sequence analysis and phylogenetic reconstruction

Sequences were retrieved by BLASTp searches of the NCBI non-redundant database using *S. scrofa* EOGT protein sequence (ENSSSCP00000012260) and *S. scrofa* OGT protein sequence (ENSSSCP00000013198) as a query (Data 1 in Supplementary Material). Only sequences that aligned the entire length with an *e*-value not higher than 10^−6^ were kept for multiple sequence alignment in order to keep as much informative sites as possible for phylogenetic reconstruction. Sequences of a same species that were 100% identical to one another or entirely included in a longer one were eliminated to remove redundancy. We performed phylogenetic analyses using two different approaches, Bayesian estimation and bootstrapped maximum likelihood (ML). The phylogenic reconstruction was performed with 48 sequences from model insect species (*Drosophila melanogaster*, *Bombyx mori*, *Anopheles gambiae*, *Aedes aegypti*, and *Apis mellifera*) as well as from other models or agronomical species (*Mus musculus*, *Rattus norvegicus*, *Danio rerio*, *Tetraodon nigroviridis*, *Takifugu rubripes*, *Xenopus laevis*, *Xenopus tropicalis*, *Bos taurus*, *Equus caballus*, *Sus scrofa*, *Gallus gallus*, and *Homo sapiens)*. Sequences were aligned using MUSCLE (30) with default parameters and the multiple sequence alignment was manually examined using JALVIEW (31, 32) (Data 2 in Supplementary Material). We performed phylogenetic analyses using ML estimation with the RAxML software (33). We systematically ran 100 bootstrap replicates followed by a ML search for the best-scoring tree. We chose WAG model of amino acids evolution because it returned the best posterior probability score in the corresponding Bayesian phylogenetic analysis (data not shown) done using MRBAYES software (34). We used a model with four categories of estimated gamma rates of evolution as well as an estimate of the proportion of invariable sites. The tree was generated using MEGA 5 (35) and is presented with bootstrap values. Each protein was also assessed for targeting to the extracellular space using two different prediction methods: WoLF PSORT (36) and SignalP 4.0 (37).

## Results

### Analysis of the profile of nasal mucus soluble proteins by one-dimensional native-PAGE

**Figure 1:**
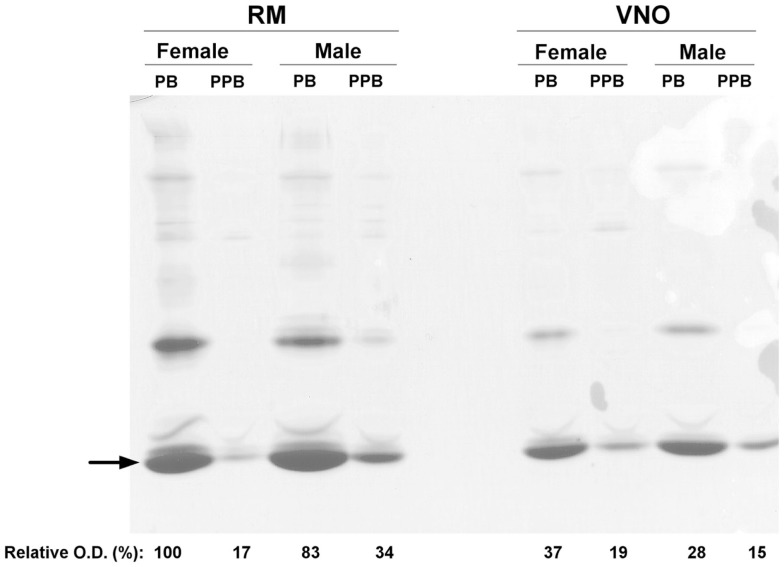
**One-dimensional native-PAGE of soluble proteins extracted from respiratory mucosa (RM) and vomeronasal mucosa (VNO) of pigs**. The gel was stained with colloidal Coomassie blue. Wells were loaded for each tissue as follows: pubertal female (PB), pre-pubertal female (PPB), pubertal male (PB), and pre-pubertal male (PPB). Arrow: faster migrating band used as a standard for quantification and normalization of the samples. Relative optical density (in %) was calculated by using ImageJ software.

Soluble proteins were extracted from each tissue (RM or VNO) of each animal (pre-pubertal male, pre-pubertal female, pubertal male, and pubertal female). Proteins were separated by one-dimensional native-PAGE, in order to confirm the range of pH to be used further in 2-DE. The most abundant proteins are localized in the lower part of the gel (Figure 1) indicating an acidic pI, consistent with the theoretical values calculated from porcine OBPs primary sequences: 4.23 for OBP, 4.89 for VEG, and 5.07 for SAL. Strips in the pI range of 3–5.6 were thus used in 2-DE analyses. The quantity of each sample corresponds to 1/250 of the proteins extracted from 30 mg of tissue. This analysis allowed standardization of the protein amount for 2-DE, the quantity 1X corresponding to 1 μg of the faster migrating band (Figure 1, arrow). Mainly quantitative changes in the protein composition of samples were observed, with higher quantities in pubertal male and female RM and VNO than in pre-pubertal samples (Figure 1).

### Analysis of olfactory proteome from male RM by 2-DE

**Figure 2:**
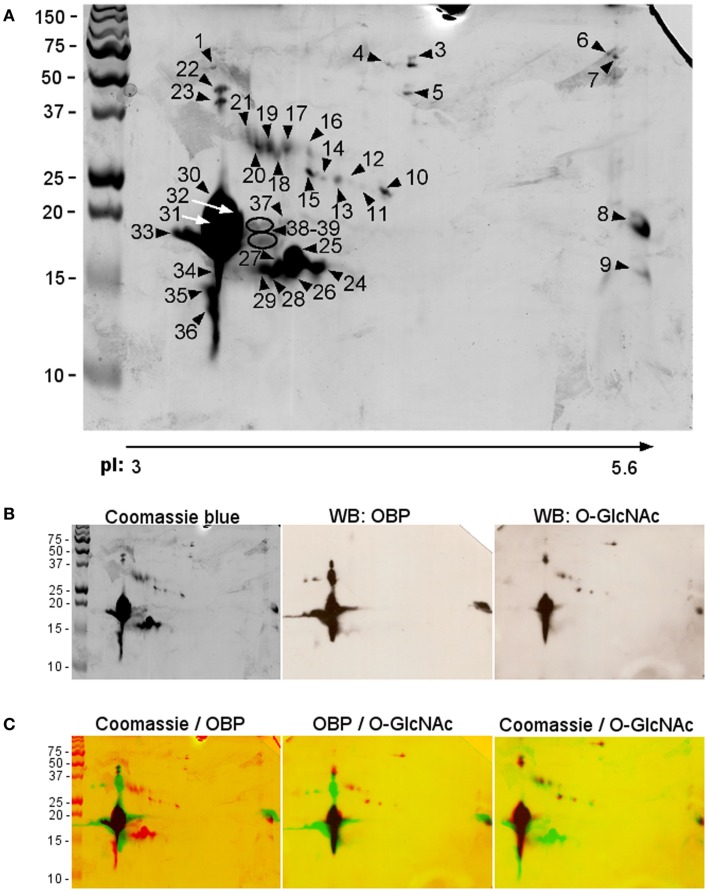
**Two-dimensional electrophoresis of soluble proteins extracted from pubertal male respiratory mucosa**. **(A)** Coomassie blue staining. Protein spots were identified by mass spectrometry (numbering corresponds with data in Tables 2 and 3). **(B)** Raw images used in the merges, Coomassie blue staining, western-blot with anti-OBP, and RL2 antibodies. **(C)** Merges of the three images above.

We proceeded with 2-DE, as it provides a more detailed pattern of soluble proteome diversity. Image analysis of 2-D gels of an adult male sample (Figure 2A) revealed a majority of protein spots in the predicted region of pI (3–5.6) and in the MW range of 10–50 kDa. After trypsin digestion, 39 protein spots (numbering in Figure 2A) were subjected to Nano-ESI-LC-MS/MS (protein spots of minor intensity, Table 2) or to MALDI-TOF MS (protein spots of major intensity, Table 3) analyses. The 39 spots were analyzed successfully, resulting in the identification of 10 different proteins in the gel. Among them, seven proteins dispatched in eight spots did not belong to the OBP family: selenium-binding protein (SELENBP1), alpha-1-acid glycoprotein (AGP), vitamin D-binding protein (DBP), haptoglobin precursor (HG), hemopexin precursor (HPX), superoxide dismutase (SOD), and lactoylgluthathione lyase (LGL).

**Table 2:** **Identification by Nano-ESI-LC-MS/MS of proteins from pubertal male respiratory mucosa**.

Spot No.	Protein identification	NCBI accession number	Score	Percentage sequence covered (peptide matched)	*O*-GlcNAc signal
1	Selenium-binding protein (SELENBP1)	gi|194036227	113	19 (2)	
	Alpha-1 acid glycoprotein (AGP)	gi|164302	40	5 (1)	
3	Vitamin D-binding protein (DBP)	gi|335293644	137	12 (9)	+
4	Vitamin D-binding protein (DBP)	gi|335293644	149	10 (8)	
5	Haptoglobin precursor (HG)	gi|47522826	139	15 (6)	
6	Hemopexin precursor (HPX)	gi|47522736	47	4 (3)	
7	Hemopexin precursor (HPX)	gi|47522736	53	23 (25)	
9	VEG	gi|27657971	405	28 (20)	
	Superoxide dismutase (SOD)	gi|15082144	51	6 (3)	
11	SAL	gi|21465464	110	28 (5)	
12	SAL		132	20 (6)	
14	SAL		477	52 (20)	
16	SAL		298	41 (16)	+
18	SAL		622	37 (28)	
37	OBP	gi|3122574	560	51 (27)	
	VEG	gi|27657971	85	12 (3)	
38	OBP	gi|3122574	512	32 (23)	
	VEG	gi|27657971	50	4 (1)	
39	OBP	gi|3122574	418	44 (18)	+
	VEG	gi|27657971	63	12 (4)	

*Spots labeled by RL2 antibody are indicated in the last column*.

**Table 3:** **Identification by MALDI-TOF MS of proteins from pubertal male respiratory mucosa**.

Spot No.	Protein identification	Percentage sequence covered (peptides matched)	Observed MW (kDa)	Comments	*O*-GlcNAc signal
8	OBP	72 (9)	18	With N-ter & C-ter	+
10	Lactoylgluthathione lyase (LGL)	15 (9)	23		+
13	SAL	32 (7)	25		+
15	SAL	35 (6)	26		+
17	SAL	60 (8)	32		+
19	SAL	25 (5)	32		
20	SAL	38 (6)	32		+
21	SAL	21 (4)	34		+
22	OBP	62 (7)	48	With N-ter & C-ter	+
23	OBP	33 (6)	40	With C-ter	+
24	VEG	50 (6)	15	No N-ter	
25	VEG	82 (9)	17	With N-ter & C-ter	
26	VEG	66 (9)	15	No N-ter	
27	VEG	62 (8)	15	No N-ter	
28	VEG	60 (6)	15	No N-ter	
	OBP	32 (6)		No N-ter	
29	VEG	57 (6)	15	No N-ter	
	OBP	15 (3)		No N-ter	
30	OBP	27 (5)	21	With N-ter & C-ter	+
	VEG	10 (1)		With N-ter & C-ter	
31	OBP	45 (5)	20	With N-ter & C-ter	+
	VEG	10 (1)		With N-ter & C-ter	
32	OBP	41 (7)	20		+
	SAL	18 (5)			
33	OBP	57 (9)	18	With N-ter & C-ter	
	VEG	10 (1)		With N-ter	
34	OBP	50 (6)	15	No N-ter	+
35	OBP	31 (6)	14	No N-ter	+
36	OBP	40 (7)	13	No N-ter	+

*Spots labeled by RL2 antibody are indicated in the last column*.

The most abundant proteins (in number and quantity) identified in male RM proteome are isoforms of the three proteins already described in the nasal mucus of pig (29, 38): 15 isoforms of OBP (spots 8, 22–23, 28–36, 37–39), 11 isoforms of SAL (spots 11–21), and 13 isoforms of VEG (spots 9, 24–33, 37–39). The protein spots 28–31, 33, and 37–39 contained OBP and VEG in mixture, spot 32 contained OBP and SAL in mixture. OBP isoforms were found in the acidic part of the gel, except spot 8 (Figure 2A). The main difference between OBP isoforms lies in molecular weight. Isoforms at around 20 kDa correspond to the theoretical MW of 17835 Da, while those at around 50 kDa correspond to dimers, which are ever observed, even in denaturing conditions. This tendency to strong aggregation is a typical feature of porcine OBP, and dimers are difficult to dissociate without 8 M urea treatment and overnight heating (39). More surprising is the observation of OBP isoforms at lower molecular weight, between 13 and 15 kDa. In MALDI-TOF MS, we have analyzed the spectra to find out what part of the sequence is missing (Table 3). In shorter forms, the N-terminal part of the protein is missing [peptide (1–15)], never the C-terminal ones.

Thirteen isoforms of VEG were separated by 2-DE. Isoforms in spots 30, 31, 33, 37, 38, and 39 were in mixture with OBP isoforms and have an expected apparent molecular mass of 17–18 kDa. Isoforms of spots 24, 26, 27, 28, and 29 have a lower apparent molecular mass of 15 kDa, corresponding to the loss of the first N-terminal 16 amino acids. VEG isoform of spot 25 has a higher MW and an N-terminus, but has a lower apparent MW than the forms contained in spots 37–39. Isoform of spot 9 has a MW of 15 kDa and an apparent “basic” pI of 6, as well as spot 8 containing OBP. The spot 33, containing both OBP and VEG, is the most acidic ones of the profile. Only one primary sequence was observed, corresponding to the expected form described in RM (Leu141), and not to the VNO form (Pro141) (38). It is interesting to notice that VEG dimers were not observed, as spots 22 and 23 contained only OBP isoforms.

The pattern of the 11 SAL isoforms is in accordance with the theoretical MW of 19916 Da (Figure 2A). Two nucleotide sequences were reported for porcine SAL (38). The mass spectra obtained by MALDI-TOF MS after trypsin digestion of spots 13, 15, 17, 19–21 indicated that the primary sequence corresponds to the product of gene SAL1 referenced as NM_213814. The masses corresponding to amino acids replacements Val45 →Ala, Ile48 →Val, and Ala73 →Val described in Scaloni et al. (38) were not found. The increase of molecular weight from spot 11 to spot 21 can easily been explained by the fact that SAL is *N*-glycosylated on Asn53 (38, 40). The polymorphism of glycan chains, the structure of which is still unknown, could generate SAL sub-populations of different MW, and, if processed as other mammalian *N*-glycans, of different pI due to sialic acids in terminal position. It could be noticed that SAL isoforms are of expected MW or higher, contrary to OBP and VEG that display shorter isoforms. Shorter forms lacking the N-terminus could come from protease degradation, but there are no obvious reason to explain why SAL would be protected against enzymatic action, and not OBP and VEG. In addition, action of proteases would generate much more random forms and not specifically forms lacking the N-terminal peptide.

### Search for OBP and VEG splicing variants by RACE-PCR and “*in silico*” analysis

Interestingly, the sequence of the identified OBP and VEG isoforms with apparent MW of around 15 kDa always lacks the N-terminus (Table 3). VEG isoforms of lower MW were already observed in human tears [four isoforms of 14–16 kDa in Ref. (41, 42)]. The origin of N-terminal heterogeneity of VEG has been debated and led authors to speculate involvement of genetic polymorphism. The structure of porcine VEG encoding gene (called *LCN1*, GenBank #U96150) is known (43), but not its regulation. So, we have first hypothesized that this short OBP and VEG isoforms could come from alternative splicing of their coding gene. Amplification of potential transcripts of different sizes was undertaken several times and on different animals by RACE-PCR. We obtained each time a single transcript of full-length sequence for VEG (data not shown). This is consistent with expression studies of human *VEG/LCN1* gene, that showed a single size of RT-PCR products obtained with specific primers in not only olfactory tissue, but also in lachrymal gland, placenta, and mammary gland (19). The search for short variants of OBP by RACE-PCR was also unsuccessful and we obtained a single-size PCR product around 500 bp (data not shown). Since these results, the pig genome has been sequenced and made available at Ensembl database. We have thus performed blast analysis to search for putative splicing variants for OBP, VEG, and SAL. The *SAL1* gene (Ensembl: ENSSSCG00000005474) has only one transcript (ENSSSCT00000006020) corresponding to the coding of the full-length (mature) protein of 175 amino acids. The gene encoding porcine VEG, *LCN1* (ENSSSCG00000024779) has two transcripts corresponding to a mutation leading to the VNO form (AAB34720.1, Pro141) and the RM form (AAO18367.1, Leu141), both already described (38). The sequences of the two variants coming from the alternative splicing of exon 3 of the OBP gene (ENSSSCG00000012095) were obtained from Ensembl: transcript ID ENSSSCT00000013229 of 522 bp coding for the mature protein of 158 amino acids already described (GenBank RefSeq NM_213796, OBP1), and transcript ID ENSSSCT00000033772 of 605 bp encoding a mature protein of 156 aminoacids (OBP2, Ensembl ENSSSCP00000028674). Both proteins have the same theoretical pI of 4.23 (calculated with expasy.org/protparam/). The proteins differ from the amino acid 82 (underlined): ^81^NYAGNNKFV^89^ for OBP1, ^81^NCNNKFV^87^ for OBP2. On MALDI-TOF spectra, the two proteins could be distinguished by the peptide at m/z 1498.6227, only found for OBP2 ([73–85] peptide) but this mass is closed to that of peptide at m/z 1498.7424 obtained only for OBP1 digestion ([16–28] peptide). The two OBP transcripts encode a C-terminal Lysine as already described (29). However, OBP isoforms of male RM all miss the Lysine in C-terminal, indicated by the peak at m/z 2296.0714 Da corresponding to the peptide [138–157] with the Alanine as terminus. This could come from proteolysis degradation or by alternative expression of two alleles of the OBP gene as it was already suggested (29).

### Immunodetection of *O*-GlcNAcylation

In a previous work, we have shown that a VEG isoform is modified by an *O*-GlcNAc moiety (27). We have performed western-blot on an aliquot of male RM soluble proteome separated by 2-DE in the same conditions as above. The specific antibody RL2 (Figure 2B, WB: *O*-GlcNAc) labeled mostly OBP isoforms (Figure 2B, WB: OBP), including the “acidic” full-length (spot 8) or short-length (spots 34–36) isoforms, and the “dimers” in spots 22–23. This labeling is highly specific because short-length VEG isoforms in spots 24–29 (and VEG in spot 25 of MW 17 kDa) are not labeled at all, despite their quantity (Figures 2B,C: Coomassie/*O*-GlcNAc). This constitutes a negative control of the RL2 specificity for these proteins. The “basic” spot (9) contains also a short-length VEG isoform and is not labeled by RL2 (Figure 2C: Coomassie/*O*-GlcNAc). Contrary to OBP isoforms of short size, VEG isoforms of short size are never labeled by RL2, suggesting that the modification occurs on the N-terminal part that is missing in these forms (peptide [1–16]). This also suggests that the VEG isoform identified previously (27) as *O*-GlcNAc modified is of full-length sequence and probably contained in spots where OBP and VEG are in mixture, and labeled by RL2 antibodies (spots 30–32, 39). Meanwhile, VEG isoform of spot 25, with N-terminal and C-terminal has a smaller apparent molecular weight than those of isoform contained in spots 39. This difference could be attributed to the modification of VEG 39 by *O*-GlcNAc, immunoreactive to RL2, whilst VEG 25 is not labeled by RL2. Eight spots containing SAL isoforms were labeled by RL2: 13, 15–17, 20–21, and spot 32 containing OBP and SAL in mixture (Figure 2C). Concerning proteins unrelated to OBPs, spot 3 containing DBP and spot 10 containing LGL were labeled by RL2.

### Comparison of olfactory proteome between tissues of the same animal (male RM and VNO) and between male and female RM

**Figure 3:**
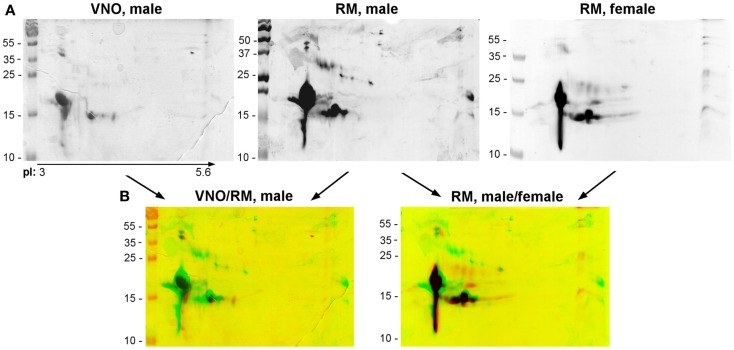
**Comparison of soluble proteins profiles between tissues (RM and VNO) of adult male, and between RM of male and female**. **(A)** Coomassie blue staining. **(B)** Merges of Coomassie blue stained gels.

**Figure 4:**
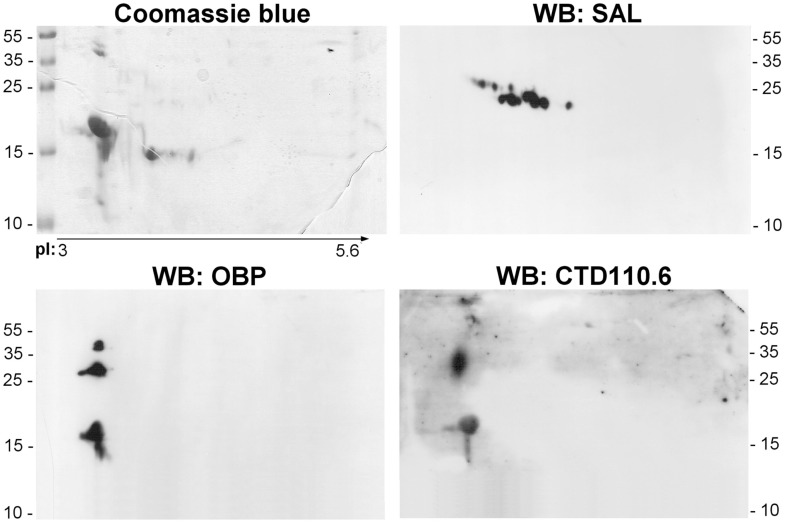
**Analysis of male VNO soluble proteome**. Aliquots of the same sample were used for Coomassie blue staining, and western-blot with anti-OBP, anti-SAL, and CTD110.6 antibodies.

For ethical reasons, it is difficult to obtain littermates to constitute replicates. So, we have in a first approach compared the soluble proteome: (1) between VNO and RM of pubertal male and (2) between male and female RM. Soluble proteins from these tissues were separated in the same conditions than the RM sample analyzed above (Figure 3A). The three profiles obtained after 2-DE are similar, but slight differences were observed. The quantity and number of isoforms is higher in the male RM soluble proteome, since the samples correspond each to the same weight of tissue (30 mg) before protein extraction. In addition, the merge of images of the two tissues stained by Coomassie blue (Figure 3B: VNO/RM, male) indicates a different pattern of OBP, VEG, and SAL isoforms. In the VNO, six isoforms of OBP and three to four isoforms of VEG are visible. The comparison between male and female RM showed less differences (Figure 3B: RM, male/female). In particular, the pattern of OBP and VEG isoforms in female sample is similar to those of male, except that SAL and the basic OBP are not visible in the female. Interestingly, a spot of the VEG pattern is similar to spot 25 of male RM. At first glance, no spot can be observed at the position of SAL isoforms migration on the gel stained by Coomassie blue, either in VNO nor in female RM. However, western-blot with anti-SAL antibodies revealed 13 spots in male VNO (Figure 4, WB: SAL) in a similar distribution to isoforms of RM extract (Figure 3A). An aliquot of male VNO was treated by western-blot with CTD110.6 antibodies, and only OBP isoforms were labeled (Figure 4: WB: CTD110.6). This result is surprising because SAL isoforms are labeled by RL2 in male RM (Figure 2C: OBP/*O*-GlcNAc).

### Molecular cloning of *Sus scrofa EOGT* cDNA and phylogenetic analysis

**Figure 5:**
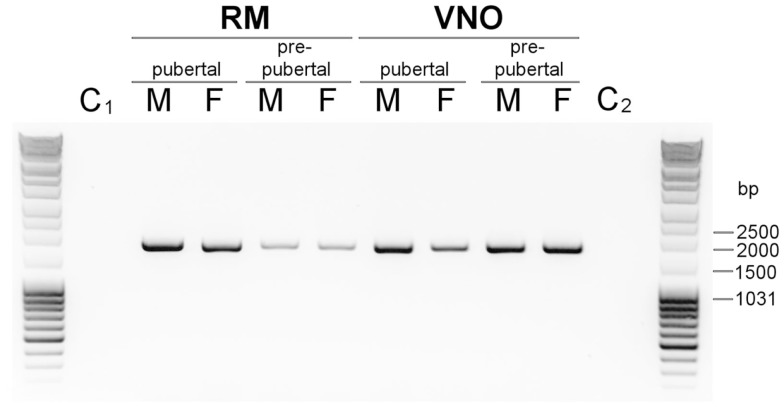
**Expression of *Sus scrofa* EOGT in RM and VNO of males and females**. Agarose gel (1% in Tris Acetate EDTA buffer) electrophoresis of PCR products amplified with specific primers designed from 5′ and 3′ ends of transcript ID ENSSSCT00000012589 (Ensembl database, *Sus scrofa* genome). C_1_, and C_2,_ controls: PCR mix without cDNA.

The report of a new OGT in *Drosophila*, linking a single *O*-GlcNAc moiety on Ser and Thr residues of secreted proteins (28) supported our findings on OBP *O*-GlcNAcylation. The availability of pig genome sequence gave us the opportunity to identify and clone the *EOGT* cDNA from pig olfactory tissues. We have blasted the pig genome (Ensembl database) with *D. melanogaster EOGT* sequence and designed primers from the 3′ and 5′ ends of the transcript ID ENSSSCT00000012589. A single PCR product was obtained in all tissues of RM and VNO from pre-pubertal and pubertal males and females (Figure 5), but not in the controls (PCR mix without DNA template or without DNA polymerase). The PCR product from male RM was cloned and sequenced. The cDNA sequence is 1696 bp long, and starts with the ATG codon for initiating the translation and a predicted signal peptide of 18 amino acids [SignalP, (37)], leading after cleavage to a mature protein of 509 amino acids. The *EOGT* sequence obtained from pig tissue was 100% identical to the unique transcript ID ENSSSCT00000012589 reported in Ensembl database (pig genome). The full-length cDNA sequence of *S. scrofa EOGT* was deposited in GenBank database under accession number JX546149. *S. scrofa* EOGT is 48.9% identical to *D. melanogaster* EOGT.

**Figure 6:**
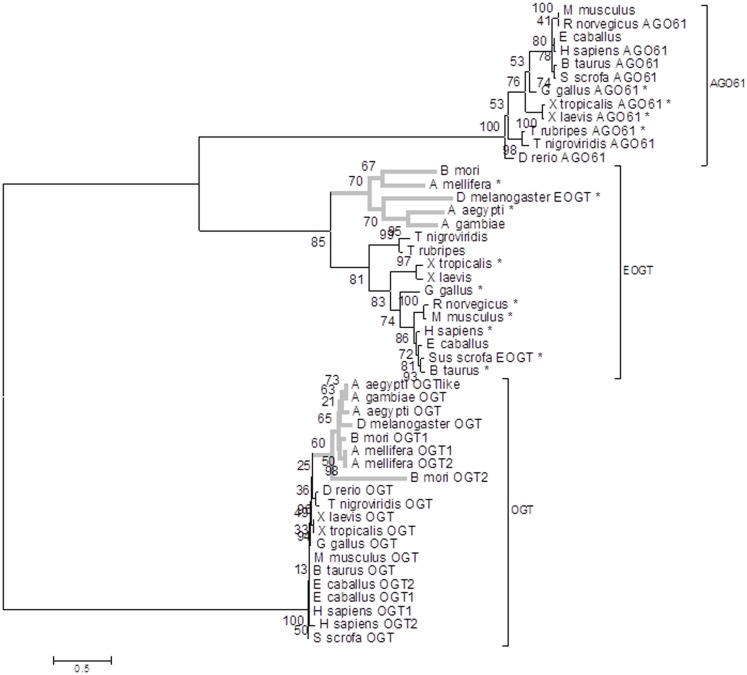
**Maximum likelihood phylogenetic tree of genes encoding OGT, EOGT, and AGO61**. RAxML bootstrap support values are shown at the nodes of the tree. *Outlined secreted proteins detected by WoLF PSORT or SignalP 4.0. Protostomes are indicated in gray lines.

To confirm the cloning of an *EOGT* in *S. scrofa* we performed a phylogenetic analysis. Glycosyl transferases (EC 2.4.x.y) are classified according to their enzymatic activities as well as their structure in the Carbohydrate-Active enzyme (CAZy) web site [(44)]^2^. *S. scrofa* OGT is a GT from the characterized family GT41 with UDP-GlcNAc (EC 2.4.1.94) and UDP-Glc (EC 2.4.1.-) known activities, whereas *S. scrofa* EOGT and *S. scrofa* AGO61 belong to the characterized family GT61 with β-1,2-xylosyltransferase (EC 2.4.2.38) and *O*-β-N-acetylglucosaminyltransferase (EC 2.4.1.94). OGT, EOGT, and AGO61 proteins clustered in two different branches of our phylogenic tree (Figure 6) with EOGT and AGO61 proteins belonging to two different branches of the same sub-tree. OGT and EOGT groups sequences from deuterostomes and protostomes (in gray) phyla whereas AGO61 branch only groups deuterostome sequences. Most of the sequences clustered in the EOGT branch are predicted as targeted to the extracellular space, which is in agreement with the function of the EOGT described by Sakaidani et al. (28).

## Discussion

The proteomic analysis of pig nasal mucus described above brought important novelty at several aspects:

### Soluble proteome of pig nasal mucus is mainly composed of OBP, VEG, and SAL isoforms

The composition of the pig nasal mucus has never been investigated by 2-DE. However, body fluids such as tears, saliva, and nasal mucus could provide important information on health conditions of animals and humans, as alterations in their profile could be associated with specific diseases (45). Until now, such studies concerned proteomic analyses of body fluids in which VEG and SAL are also secreted, tears (41) and saliva (46, 47), but not OBP, as it is specific to the nasal area. Three VEG and five SAL isoforms were identified in porcine saliva (47), and four VEG isoforms in human saliva (46). The human saliva did not contain any SAL isoform, as the encoding gene *SAL1* is a pseudogene in this species (48). But in both cases, these isoforms are not the major proteins (<1% of the total spots). On the contrary, porcine nasal mucus is mainly composed of OBP, VEG, and SAL isoforms, whatever the animal (male or female) or tissue (male RM or VNO). Indeed, only seven proteins identified in eight spots were not OBPs: SELENBP1, AGP, DBP, HG, HPX, SOD, and LGL. Most of these proteins have antioxidant properties and are involved in the protection of cells against oxidative stress. They function probably to protect the cells of the olfactory epithelium exposed to oxygen. Their expression level could be a marker of oxidative stress in nasal mucus. The 32 other spots contained isoforms of OBP, VEG, and SAL, alone or in mixture. The comparison between tissues and animals of the two sexes showed similar profiles, which gives a good confidence in the reproducibility of our analyses. The differences observed in isoforms number and distribution could either be due to inter individual variability, frequently observed in mammals, or to differential expression under hormonal control. This later hypothesis is supported by numerous works on the olfactory system, which displays a high plasticity at different moments in the individual life, determined by hormonal switch, such as puberty [e.g., Ref. (49, 50)]. In pig, the perception of pheromones is differently interpreted according to age and sex of the animals. For example, the sex steroid androstenone is abundant in the saliva of sexually active males and induces acceptation of the male in estrus females (heat period), but not in di-estrus females (2). When perceived by pre-pubertal animals, it has appeasing effects and is a signal of submission for piglets (51). This multiple role in social relationships is due to the endocrine status of the receiver animal, in particular to the level of testosterone, the hormone of puberty in males that is very low in pre-pubertal individuals of both sexes, and in adult females. Considering the high and rapid turnover of OBPs (14) at the peripheral level, these proteins are good targets for olfactory plasticity and we could hypothesize that their post-translational regulation is under control of hormones determining the endocrinal status of animals.

### OBP isoforms are potentially modified by atypical *O*-GlcNAcylation

In a previous work, we have identified an *O*-GlcNAc moiety on a VEG isoform that seems to determine its specific binding to testosterone (27). This PTM affects nuclear and cytoplasmic proteins (52, 53), *via* the action of a cytosolic GT, the *O*-linked *N*-acetylglucosaminyltransferase [OGT, (54)]. This dynamic modification of Ser/Thr residues was not supposed to affect proteins of the secretion pathway that are processed in Endoplasmic Reticulum, Golgi, and secretion vesicles. Meanwhile, since 2008, some authors reported the presence of *O*-GlcNAc on extracellular domains of receptors, which are synthesized through the secretion pathway. Atypical *O*-GlcNAcylation was initially reported as specific to the 20th EGF domain of recombinant Notch expressed in insect cells (55). Thus, the enzyme that catalyzes the *O*-GlcNAcylation of extracellular domains of Notch or Dumpy in *Drosophila*, EOGT, was proposed to modify EGF repeats of these proteins (28, 56). Our results clearly show that RL2 and CTD110.6 antibodies labeled some OBP isoforms in a specific manner, as VEG isoforms were never labeled. OBP do not display any EGF repeat in its sequence (nor VEG or SAL), but is potentially modified by *O*-GlcNAcylation. Interestingly, ORs and VNO receptors, which do not have EGF repeats in their sequence, have been characterized by Click-chemistry as *O*-GlcNAc modified (57). Extensive data have now highlighted the importance of *O*-GlcNAcylation in intracellular signaling (58), and it is not so surprising to consider its involvement in extracellular signaling, especially crucial in pheromone communication. Indeed, at an evolutionary point of view, the accurate perception of pheromone determines the fitness of an individual, and more largely, is one of the strongest prezygotic reproductive barriers involved in interspecific isolation (59). OBPs and ORs are parts of the first step of odors and pheromone reception, and so, it is reasonable to suggest that their binding properties need to be regulated by fine molecular mechanisms, such as *O*-GlcNAcylation.

### Is *Sus scrofa* EOGT responsible for *O*-GlcNAcylation of OBP isoforms?

We have cloned and sequenced the cDNA encoding EOGT in *Sus scrofa* from tissues coming from RM and VNO in animals of different age and sex. Thus, it seems that EOGT is expressed whatever the physiological status of pigs. In addition, the phylogenetic analysis confirmed that our corresponding *Sus scrofa* protein clustered with the characterized DmEOGT (28) as well as with mammalian EOGT orthologs. They belong to a specific EOGT phylogenic sub-group where the majority of proteins are predicted as targeted to the extracellular space. This EOGT sub-group clearly segregates from the two other sub-groups AGO61 and OGT. AGO61 is a putative GT from the same family as EOGT in the CAZy database (60). One *Sus scrofa* gene is found in each of these sub-groups but the *Sus scrofa* gene described in this paper is the ortholog of *DmEOGT*. This reinforces the claim that extracellular *O*-GlcNAcylation is a fundamental biochemical mechanism conserved during evolution. In particular, this process is theoretically conserved in model species where the role of OBPs is extensively studied: *B. mori* (moth), *A. mellifera* (bee), *D. melanogaster*, and *A. aegypti/gambiae* (mosquitoes) for insects, and *S. scrofa*, *R. norvegicus*, and *M. musculus* for mammals. It would be of interest to search for OBP *O*-GlcNAcylation in these species, as a potential mechanism for odor and pheromone discrimination by OBPs.

The fact that an ortholog *EOGT* gene exists in the pig genome does not mean that the enzyme is responsible for *O*-GlcNAc modification of secreted OBPs. The EOGT-like AGO61 could be a candidate for *O*-GlcNAcylation of secreted proteins, but this hypothesis was eliminated after demonstration that mouse EOGT was the sole enzyme to catalyze the linking of *O*-GlcNAc on Notch1 (61). More experiments are required to demonstrate the role of *Sus scrofa* EOGT. The EOGT could be expressed in the yeast, since no similar sequence to those of EOGT has been found yet in the databases. Thus, the EOGT purified from the yeast would come only from overexpression of the porcine form. We have already overexpressed the porcine OBP in *Pichia pastoris* with very good yields (26) and the linking of *O*-GlcNAc on recombinant OBP by recombinant EOGT could easily be monitored with UDP-GlcNAc as substrate. Alternatively, expression of OBP in mammalian cell lines deficient in EOGT would be useful.

### Binding specificities of OBP isoforms could be driven by PTM pattern

Previously, we have shown that OBP and VEG are modified by phosphorylation (25), which is also an unusual PTM for secreted proteins, although several reports of extracellular phosphorylation were published (62, 63). Moreover, binding experiments indicated that recombinant OBP isoforms display different binding affinities for the pheromone components in the pig (26). None of these isoforms binds testosterone, the natural and specific ligand of *O*-GlcNAc modified VEG (27). The results obtained here indicate that porcine OBP is modified by *O*-GlcNAc. Implications of PTM in OBP binding properties need to be investigated. First of all, purification of these isoforms has to be performed as it was done for recombinant OBP isoforms (26). Then, localization of PTM sites (phosphorylation and/or glycosylation) by high-resolution mass spectrometry would permit to rely their PTM patterns to specific binding properties. Our results indicate that *O*-GlcNAcylation could be a fine mechanism to control OBP binding specificity. These data draw a new scheme of interactions between OBPs and their odorant ligands. OBP should no more be considered as passive carriers, but more likely as players of the first step of odor and pheromone coding by the olfactory system.

## Author Contributions

Patricia Nagnan-Le Meillour, Anne-Sophie Vercoutter-Edouart, Frédérique Hilliou, Chrystelle Le Danvic, and Frédéric Lévy conceived and designed the experiments. All authors performed experiments and wrote the paper.

## Conflict of Interest Statement

The authors declare that the research was conducted in the absence of any commercial or financial relationships that could be construed as a potential conflict of interest.

## Supplementary Material

The Supplementary Material for this article can be found online at http://www.frontiersin.org/Journal/10.3389/fendo.2014.00202/abstract

## Footnotes

^1^http://expasy.org/tools/peptide-mass.html

^2^http://www.cazy.org
