# Supplementary material for: Proteomic Analysis of Pig (Sus scrofa) Olfactory Soluble Proteome Reveals O-Linked-N-Acetylglucosaminylation of Secreted Odorant-Binding Proteins
Source: Front Endocrinol (Lausanne). 2014 Dec 5;5:202. doi: 10.3389/fendo.2014.00202 (PMC4257092; doi:10.3389/fendo.2014.00202)
Supplement: Supplementary file 1 [file Data_Sheet_1.PDF]

Supplementary Material

Proteomic analysis of pig (*Sus scrofa*) olfactory secretome reveals *O*-linked-N-acetylglucosaminylation of secreted Odorant-Binding Proteins

Patricia Nagnan-Le Meillour[1]*, Anne-Sophie Vercoutter-Edouart[2], Frédérique Hilliou[3], Chrystelle Le Danvic[4], and Frédéric Lévy[5]

[1] INRA, USC-Unité de Glycobiologie Structurale et Fonctionnelle, CNRS UMR 8576, Université de Lille 1, Villeneuve d'Ascq, France
[2] CNRS, UMR 8576 Unité de Glycobiologie Structurale et Fonctionnelle, Université de Lille 1, Villeneuve d'Ascq, France
[3] INRA, UMR 1355 Institut Sophia Agrobiotech, CNRS UMR 7254, Université de Nice Sophia Antipolis, Sophia Antipolis, France
[4] UNCEIA, Unité de Glycobiologie Structurale et Fonctionnelle, Villeneuve d'Ascq, France
[5] INRA, UMR 85 Unité de Physiologie de la Reproduction et des Comportements, CNRS UMR 7247, Université François Rabelais, Haras Nationaux, Nouzilly, France

*** Correspondence:** Dr. Patricia Nagnan-Le Meillour, INRA, USC-Unité de Glyobiologie Structurale et Fonctionnelle, CNRS UMR 8576, Université de Lille1, Bâtiment C9, Cité Scientifique, Villeneuve D'Ascq, 59655, France.
patricia.le-meillour@univ-lille1.fr

1. Supplementary Data 1

>Apis_mellifera_328779613_eOGT.pro

mmidhalsikcsvtivivfavtstysnytdidlpsdhikyyfnyfptvaqecrnntvcpykdsldtkacwgyepnckae

nsfsvpqcpgdhrgwvttkkaqvetfyaqgdfgyvrdqrkemsifceplfvddsslecsehmrfcrarniminftdlir

rkepirykmdvlkegqiggyctlnekrleenadhisplqswgpelrnfrklsrppivnhdcdiviekptfvmkidaivn

myhhfcdffnlyaslhvnlshpagfstdnhimiwesysyrsafqdafeaftrnplwdlktfrgetvcfknlvfpllprm

ifglyyntpliygcensglfkafgehvlhrlriplherknqrirvtllsrdtqyrrilnedelvralkenplykvkkvv

ynkkvsfkkqleitrnsdifigihgaglthlmflpdwaavfeiyncedpgcykdlarlrgvkyftwennsklvqqdpgt

hpdggahakftnysfdveeflrivsqatdyvknhdsfknfvakkiqhkgtemknqtnvmsdvkesatskskevtelksd

trskdel

>bombyx_mori_BGIBMGA005921_eOGT.pro

MLNFTGLVGRGDNLRYKTDILSAGQIGGYCKFYSDRLMKEAEHMSALQSWGPEMVNFVKTPK KPIADGMCDIVIDKPTY

IMKLDAGWETFTYDSAFKDAFKAFTENPIWDLKRFRGKVVCFKNAVFPLLPRMIFGLYYNTPLIYGCERSGLFHAFSKH

ILHSLNIKLHMRTDDRVRVTLLSRGTTYRSILNEKEIVDALLKEDGYYVQRVVYDRTVSFTKQLEITHNTDVFIGMHGA

GLTHLLFLPDWAAVFEIYNCEDPNCYADLSRLRGLKYVTWEDKSKLVQQDEGHSPGGGSHAKFTNYSFDVKEFLRLVAK

CAEYVRNRQDFQNFVEASLIKMHEEL

>anopheles_gambiae_AGAP009279_eOGT.pro

MVQYIPSATCGKPYEYINLPKAHLPMYFKRFPALEKQCAEDETCPYRTVIASQSYQNRKEGCWGYEEGCTERNRYANHS

CPGSHIGYVKSKQAQLDTFYSQADFGFVRDQMRETRIMCEPQFPHDSSLECSKYLRFCRGRNLMLNFTDLVHRTEPLRY

KMDVLGPGQIGGHCRLHGERLRDELQHISPLQSWGPELRFFERLERPPIETGVCDVVIERPAFIMKIDAAINMYHHFCD

FLNLYASLHVNLSHAGGFDTDTQVLVWESFTYASPFADTFKVFSKHPIADLKTYAGKVVCFKNVVLPLLPRMIFGLYYN

TPIIYGCENSGLFHAFSEHVLHRLKVRMTTRPDERVRITFLSRQTRYRRVLNEDELMGRIAKNPNYAVQRVSYGHDLPF

VEQLRITRNTDIFIGMHGAGLTHLLFLPKWATLFELYHCEDPNCYRDLARLRGVHYLTWERDHLVYPEGEGKHPERDER

HAKFTNYAFDVGEFERLVAKAAAYVQGHAEFQKFLTTNANRAVKRKDEL

>Aedes_aegypti_XP_001658768_eOGT.pro

MIRVLLVVLTFGAFAGGSKNDYEYINLPKSHLPLYFRRFPRLEKRCLKDETCEYQKLLKSEAFKAKKDTCWGYEDDCRK

ENRFSKPECPGDFHGYVKSKEAQLETFYAQADFGFVRDQIRETRIMCEPTFPHDSALECSKYLRFCRGRNIMVNFTDLI

HRTEPLRYKMDVLSHGQIGGHCKLHRKRLEDELEHISPLQSWGPELRFFDTVDKPLSQGGTCDVTIDRPAFIMKIDATI

NMYHHFCDFINLYGSLHANLSDPYGFSTDVQIMVWESYTYDSPFAETFKVFTKHPIADLKTYAGKVVCFKNLVLPLLPR

MIFGLYYNTPIITGCENSGFFQAFSEHVLHRLKVPQRSRSDRKLRITFLSRQTKFRRVLNENALL

EEISENEDYLVNQA

SFTYKTDFREQLKITRNTDIFIGMHGAGLTHLLFLPKWAVLFELYHCEDPNCYKDLARLKGVR YLSWERDDLVYPEDEG

HHPDGGGRHAKFTNYAFDAKEFARLVAIGAEHVWNHEEYQQFLERSRRKQEKLSGKDEL

>Dm_eOGT_AB675601_prot.pro

MPILPILIGILHLSLAEDAKHLDGFSLPSLPSEHLIRYLNTFPKLKQQLPTNLTGKGTISSACWGH ERDCTPAGRFQTP

QCPGEHTGWARSKEAQVRTFYNQADFGYIQEQLSQLTPQCVPTYLGDSSLECTHYLRFCRGRN LLFDFRGLEQREERIR

YHMDVLGPGQLLGHCKLNRTRLSGEMEHIGSALQSWGPELRNFDVLPHPVLESGLCDVVVNT PTFIMKIDATYNMYHHF

CDFFNLYASLFVNQSHPAAFNTDVQILIWETYPYDSPFRDTFKAFSQRPVWTLSDVEGKRVCFK NVVLPLLPRMIFGLF

YNTPIIQGCSNSGLFRAFSEFILHRLQIPYKPPQQKIRITYLSRRTKYRQVLNEDELLAPLEANDK YDVQRVSYERLPF

TNQLAITRNTDILIGMHGAGLTHLLFLPNWACIFELYNCEDPNCYKDLARLRGVRYRTWEQRD LVYPQDEGHHPEGGAH

AKFTNYSFDVKEFVHLVDGAAEEILSHKEFPRRASENPSKTQRNEL

>takifugu_rubripes_eOGT_Q5NDK8.pro

MLLEVVLGIVFPFAVVTADSATNHKPAPLLSYNGTSLPLDHVPYFLNNNKKLAKQCRSDPLCPF RVSCDALQDLSVCWG

YEKNCDPGKRFSYPVCTRADYGWTHSLETAREIFWKQADFGYVKERLSELKALCKAKKLGGS SLKCSSYTRFCKATNLY

LDLRKPRRSHERYKEDFIQKGELGGHCRLNKAALAAEGDHKSPLQSWYAELQTYTELDSDPIV NGQCDLTVDKPTVFMK

LDAGVNMYHHFCDFVNLYISQHINNSFSSDINIVMWDTSSYEYGDLFSETWRAFSQNDIIHLKVY DNKRVCFRDALFSL

LPRMRYGLFYNTPLISDCYSEGMFRAFSQHILHRLHVPQDGPKDGRVRVTLLARSTEYRKILNQ VELVNALKTVPNLEV

NVVDYKYKDVPFLVQLKTTHNSDIFIGMHGAGLTHLLFLPDWAVIFELYNCQDESCYRDLARL RGVRYVTWQQMNKVFP

QDKGHHPTLGDHPKFTNYSFDVAEFMRIVLEAADYVTRHPKWQRRTPRDEL

>tetraodon_nigroviridis_eOGT_Q5NDK9.pro

MLLEVVLVSVFSFCVVTAESPVNNTPVPLTSYNGISLPLQHVPYFFNNNKKLAKQCRSDPFCPFK VRHDALQDLSVCWG

YEKNCDPGKRFSYPVCIRADSGWTHSLETAREIFWKQADFGYVKERLSELKTLCKATKPVSLA HFLITHTRFCKTTNLY

LDLREPNRSHERYKEDFIERGELGGYCRLNKAALAAEGDHKSPLQSWYAELQTYTELDSDPIVN GQCDLTVDKPTVFMK

LDAGVNMYHHFCDFVNLYISQHINNSFSSDINIVMWDTSFYEYGDLFSETWRAFSENDIIHLKTY DSKRVCFRDAFFSL

LPRMRYGLFYNTPLISDCYSEGMFRAFSQHVLHRLNIPQDGPKDGRVRVTLLARSTEYRKILNQ VELVNALKTVPHLKV

NVVDFKYKDVPFLVQLKITHNSDIFIGMHGAGLTHLLFLPDWAVIFELYNCQDESCYRDLARLR GVRYVTWQKMNQVFP

QDKGHHPTLGDHPKFTNYSFDVAEFVRLVLEAADYVTRHPEWRRQTPRDEL

>Gg_eOGT_Q5NDL3.pro LOCUS AER61_CHICK 535 aa linear VRT 21-SEP-2011

mfillmfvlllqeilansrdenltelnsvleeptysyrainlpaehipyflhnnrhiagickqdsrcpykvgfyfvlhk

ylkklkscwgyeksckskdyrfsypvcdyvesgwandietaqqifwkqadfgyirerlnemkthckptvtgdssltcsqf

lqhcratnlyidlrtakrnherfkedffqkgeigghctldvkaflaegqrksplqswfaelqtftslnfrplddgkcdi

viekptyfmkldagvnmyhhfcdfvnlyitqhinnsfstdvnivmwdtssygygdlfsetwkaftdydiiylktfdskr

vcfkeavfsllprmryglfyntplisgchgtglfrafsqhvlhrlnitqegpkdgkirvtilarstdyrkilnqnelvn

alktvstlevkvvdykykelefseqlrithnsdifigmhgaglthllflpdwavvfelyncedercyldlarlrgihyi

twrkrnkvfpqdqghhptlgehpkftnysfdveefmylvllaanhvsqhskwpfrvkhdef

>Xenopus_laevis_eOGT_Q6GQ23.pro LOCUS AER61_XENLA 525 aa linear VRT 28-JUN-2011

mvplrlvlllhiihfscenevgsaanngsaqlynyrkihlpddhipyylhsnrhvaalclqdlhcpykqhlqnlnscwg

yektcaeghrfgypvcdqvdfgwaktieesqqvfwrqadfgyvkerlaetqilcrpqeqgdsmlacsqnlqhcratnly

ldlrhprrgqenfkedflqegeigghcdldkqallsqgawksplqswfaelqsyssfkfkpiedahcdiiiekptyfmk

ldagvnmyhhfcdfvnlyitqhvnnsfstdinivmwttsvygygdlfsdtwkaftdyeithlkaydnkrvcfkdavfal

lprmryglfyntplishchgsglfrafsqhvlhrlnitqhpateakirvtilvrstefrkilnldelvqaleavptfqv

kvvdykyrvlgfleqlsithnsdifigmhgaglthllflpdwavvfelyncedarcyldlarlrgiqymtwekgdkvfp

qdkghhpnlgehpkftnyafdveeflrlvqqgatyvsrhskwplrrtrdel

>xenopus_tropicalis_Q08CY9_eOGT.pro

MVPLWLLLLFHVIHFSHGNEIDSAASNGSALSYHYGKLYLPDDHIPYYLHSNRHIAALCRRDPH CPFKQHLQNLNSCWG

YEKSCTKGHGYSYPVCDQVDFGWAKTIEESQEVFWKQADFGYVKERLAETQILCRPQEQGDS MLACSRNLQHCRATNLY

LDLRNPRRGQENFKEDFLQEGEIGGRCNLDKQALLSQGAWKSPLQSWFAELQSYSSLTFKPVE DAHCDIIIDKPTYFMK

LDAGVNMYHHFCDFVNLYITQHVNNSFSTDINIVMWTTSVYGYGDLFSDTWKAFTDYDITHLK AYDNKRVCFKDAVFAL

LPRMRYGLFYNTPLISNCHGSGLFRAFSQHVLHRLNITQQLPKEAKIRITILVRSTEFRKILNLDE LVHALEAEPTFQV

KVVDYKYRVLGFLEQLEITHNSDIFIGMHGAGLTHLLFLPDWAVVFELYNCEDERCYLDLARL RGIRYMTWENRDKVFP

QDKGHHPNLGEHPKFTNYAFDVEEFLRLVRQAAKNVSRHSKWPFRRTRDEL

>Mm_eOGT_Q8BYW9.pro LOCUS AER61_MOUSE 527 aa linear ROD 21-SEP-2011

mlmllvfgvllhevplsgqdkahseaddapgkalydysslrlpaehipfflhnnrhvasvcredshcpykkhlenlnyc

wgyekscapefrfgspvcsyvdlgwtdtlesaqdmfwrqadfgyarerlgeirticqperasdsslvcsrylqycratg

lyldlrnikrnhdrfkedflqggeiggyckldshalvsegqrksplqswfaelqgytqlnfrpiedakcdivvekptyf

mkldaginmyhhfcdflnlyltqhvnnsfstdvyivmwdtstygygdlfsdtwkaftdydvihlktydskkvcfkeavf

sllprmryglfyntplisgcqntglfrafsqhvlhrlnitqegpkdgkvrvtilarsteyrkilnqdelvnalktvstf

evrvvdykyrelgfldqlrithntdifigmhgaglthllflpdwaavfelyncedercyldlarlrgihyitwrkpskv

fpqdkghhptlgehpkftnysfdveefmylvlqaaehvlqhpqwpfkkkhdel

>Rn_eOGT_Q5NDL0.pro LOCUS AER61_RAT 527 aa linear ROD 21-SEP-2011

mlmllvfgvllhevplsgqdeahpeadrvpgealydysslrlpeehipfflhsnrhvasvcredshcpykkhleslnsc

wgyeksctpesrfgspicsyvdlgwtdtlesaqdmfwkqadfgyarerleeirmfcrpesasdssllcsrylqycratg

lyldlrnikrnhdrfkedflqggdiggyckldrhalvsegqrksplqswfaelqgytqlnfrpiedakcdivvekptyf

mkldaginmyhhfcdflnlyltqhinnsfstdvyivmwdtssygygdlfsdtwkaftdydvihlktydskkvcfkeavf

sllprmryglfyntplisgcqntglfrafsqhvlhrlnisqegpkdgklrvtilarsteyrkilnqnelvnalktvstf

evrvvdykyrelgfldqlrithntdifigmhgaglthllflpdwaavfelyncedercyldlarlrgiyyitwqkpskv

fpqdkghhptlgehpkftnysfdveefmylvlqaaehvlqhpqwplkknhdel

>Hs_eOGT_Q5NDL2.pro LOCUS AER61_HUMAN 527 aa linear PRI 14-DEC-2011

mlmlfvfgvllhevslsgqneappnthsipgeplynyasirlpeehipfflhnnrhiatvcrkdslcpykkhleklkyc

wgyeksckpefrfgypvcsyvdmgwtdtlesaedifwkqadfgyarerleemhvlcqpketsdsslvcsrylqycratn

lyldlrnikrnhdrfkedffqsgeigghckldirtltsegqrksplqswfaelqsytqlnfrpiedakcdiviekptyf

mkldagvnmyhhfcdfinlyitqhvnnsfstdvyivmwdtssygygdlfsdtwnaftdydvihlktydskrvcfkeavf

sllprmryglfyntplisgcqntglfrafaqhvlhrlnitqegpkdgkirvtilarsteyrkilnqnelvnalktvstf

evqivdykyrelgfldqlrithntdifigmhgaglthllflpdwaavfelyncedercyldlarlrgvhyitwrrqnkv

fpqdkghhptlgehpkftnysfdveefmylvlqaadhvlqhpkwpfkkkhdel

>Ss_eOGT_ENSSSCP00000012260.pro

MLMLLVFGALLHEVPLSGQDKAPPQADGILGAPLFNYASLRLPEEHIPFFLHNNRHIATVCKKDSRCPYKKYLENLKYC

WGYEKSCKPQFRFGYPVCTYVDMGWTDTLESAEDIFWKQADFGYAGERLEELHVLCQAEEMNDSSLVCSRYLQYCRATN

IYLDLRNIKRNHDRFKEDFFQNGEIGGHCKLDIRTLMSEGQRKSPLQSWFAELQSYTQLNFRPIEDAACDIVIEKPTYF

MKLDAGVNMYHHFCDFVNLYITQHVNNSFSTDVYVVMWDTSSYGYGDLFSDTWKAFTDYDVIHLKTYDSKRVCFKEAIF

SLLPRMRYGLFYNTPLISGCQNTGLFRAFSQHVLHRLNITQQGPKDGKIRVTILARSTEYRKILNQNELVNALKTVSTF

EVRIVDYKYKELAFLDQLRITHNTDIFIGMHGAGLTHLLFLPDWAAVFELYNCGDERCYLDLA

RLRGVHYITWRRQNKV

FPQDKGHHPTLGEHPKFTNYSFDVEEFMFLVLQAADHVLQHPKWPFKNKHDEL

>Bt_eOGT_A0JND3.pro LOCUS AER61_BOVIN 527 aa linear MAM 16-NOV-2011

mfmllvfgallpevplsgqdkappqadgisatplfnyaslrlpeehipfflhnnrhiatvcrkdshcpykkylenlkyc

wgyekscrpefrfgypvctyvdmgwtdtlesaqeifwkqadfgyaaerleelhvlcqpkekndsslvcsrylqycratn

iyldlrnikrnhdrfkedfvqsgeiggyckldirslmsqgqrksplqswfaelqsytelnfrpvedaqcdiviekptyf

mkldagvnmyhhfcdfinlyitqhvnnsfstdvyvvmwdtssygygdlfsdtwkaftdydvihlktydakrvcfkeaif

sllprmryglfyntplisgcqntglfrafsqhvlhrlnitqegpkggkirvtilarsteyrkilnqnelvnalktvstf

evqivdykykelgfldqlrithntdifigmhgaglthllflpdwaavfelyncgdercyldlarlrgvhyitwrrqnkv

fpqdkghhptlgehpkftnysfdveefmylvlqaadyvlqhpkwpfkkkhdel

>eqquus_caballus_XP_003363099.pro

MLMLLVFGVLLHDIPLSGQAEASPEADGIPGEPLFNYASIRLPEEHIPFFLHNNGHIATVCKKDS HCPYKKHLENLKYC

WGYEKSCKPEFRFGYPVCTYVDMGWTDTLESAQDIFWKQADFGYAGERLGELRVLCRPEEVN DSSLVCSRYLQYCRAAN

LYLDLRNIQRNHDRFKEDFFQSGEIGGHCKLDIRTLMSEGQRKSPLQSWFAELQSYTQLPFRPIE DAKCDIVIEKPTYF

MKLDAGVNMYHHFCDFINLYITQHVNNSFSTDVHIVMWDTSSYGYGDLFSDTWKAFTDYDVIH LKTYDSKRVCFKEAVF

SLLPRMRYGLFYNTPLISGCQNTGLFRAFSQHVLHRLNITQEGPKDGKIRVTILARSTEYRKILN QNELVNALKTVSTF

EVQIVDYKYKQLGFLDQLRITHNTDIFIGMHGAGLTHLLFLPDWAAVFELYNCEDERCYLDLA RLRGVHYITWRRQNKV

FPQDKGHHPTLGEHPKFTNYSFDVEEFMYLVLQAADYVLQHPKWPFKKKRDEL

>Hs_Q8NAT1_AGO61.pro LOCUS AGO61_HUMAN 580 aa linear PRI 14-DEC-2011

mhlsavfnallvsvlaavlwkhvrlrehaatleeelalsrqatepapalridypkalqilmeggthmvctgrthtdric

rfkwlcysneaeefiffhgntsvmlpnlgsrrfqpalldlstvedhntqyfnfvelpaaalrfmpkpvfvpdvalianr

fnpdnlmhvfhddllplfytlrqfpglahearlffmegwgegahfdlykllspkqpllraqlktlgrllcfshafvgls

kittwyqygfvqpqgpkanilvsgneirqfarfmteklnvshtgvplgeeyilvfsrtqnrlilneaelllalaqefqm

ktvtvsledhtfadvvrlvsnasmlvsmhgaqlvttlflprgatvvelfpyavnpdhytpyktlamlpgmdlqyvawrn

mmpentvthperpwdqggithldraeqarilqsrevprhlccrnpewlfriyqdtkvdipsliqtirrvvkgrpgprkq

kwtvglypgkvrearcqasvhgasearltvswqipwnlkylkvrevkyevwlqeqgentyvpyilalqnhtftenikpf

ttylvwvrcifnkillgpfadvlvcnt

>eqquus_caballus_XP_001501466_AGO61.pro

MHLSAVFNALLVSVLAAVLWKHVRLREHAATLEEELALSRQAPEPAPALRIDYPKALQILMEG GTHMVCTGRTHTDRIC

RFKWLCYSNEAEEFIFFHGNTSVMLPNLGSRRFQPALLDLSTVEDHNTQYFNFVELPAAALRFM PKPVFVPDVALIANR

FNPDNLMHVFHDDLLPLFYTLRQFPGLAQEARLFFMEGWGEGAHFDLYKLLSPKQPLLRAQL KTLGRLLCFSHAFVGLS

KITTWYQYGFVQPQGPKANILVSGNEIRQFARFMTEKLNVSHTGAPLGEEYILVFSRTQNRLIL NEAELLLALAQEFQM

KTVTVSLEDYAFADVVRLVSNASMLVSMHGAQLVTALFLPRGATVVELFPYAVNPDHYTPYKT LAMLPGMDLQYVAWRN

MMPENTVTHPERPWDQGGITHLDQAEQARILQSREVPRHLCCRNPEWLFRIYQDTKVDIPSLIQ SIRRVVKGRPGPRKQ

KWTVGLYPGKVREARCQAAVQGASEARLTVSWQIPWNLKYLKVREVKYEVWLQEQGENTYV PYMLALQNHTFTENIKPF

TTYLVWVRCIFNKTLLGPFADVLVCNT

>Mm_Q8BW41_AGO61.pro LOCUS AGO61_MOUSE 605 aa linear ROD 19-OCT-2011

mhlsavfnallvsvlaavlwkhvrlrehaatleeelalgqqsldpvlglkidypkalqilmeggthmvctgrthtdric

rfkwlcysneaeefiffhgnssvmlpnlgsrrfqpalldlstvedhnaqyfnfvelpaaalrfmpkpvfvpdvalianr

fnpdnlmhvfhddllplfytlrqfpglaqearlffmegwgegahfdlykllspkqpllraqlktlgrllcfshafvgls

kvttwyqygfvqpqgpkanilvsgneirqftrfmterlnvshagaplgeeyilvfsrtqnrlilneaelllelaqefqm

ktvtvsledhtfadvvrlvsnasmlvsmhgaqlvtalflprgatvvelfpyavnpdhytpyktlatlpgmdlqyvawrn

mirentvthperpwdqggithldraeqarilqsrevprhlccrnpewlfriyqdtrvdipslmqsirrvvkgrpgprrq

rwaislypgkvrearcqasvqgatearlsvswqipwnlkylkvrevkyevwlqeqgentyvpymltlqnhtftenikpf

ttylvwvrcifnrsllgpfcrcagvqhvasrpwpglwraplagsafpgpqfc

>Rn_Q5NDF0_AGO61.pro LOCUS AGO61_RAT 580 aa linear ROD 19-OCT-2011

mhlsavfnallvsvlaavlwkhvrlrehaatleeelalgqqsldpvpglridypkalqilmeggthmvctgrthtdric

rfkwlcysneaeefiffhgnssvmlpnlgsrrfqpalldlstvedhnaqyfnfvelpaaalrfmpkpvfvpdvalianr

fnpdnlmhvfhddllplfytlrqfpglaqearlffmegwgegahfdlykllspkqpllrsqlktlgrllcfshafvgls

kvttwyqygfvqpqgpkanilvsgneirqftrfmterlnvshagaplgeeyilvfsrtqnrlilneaelllelaqefqm

ktvtvsledhtfadvvrlvsnasmlvsmhgaqlvtalflprgatvvelfpyavnpdhytpyktlatlpgmdlqyvawrn

mirentvthperpwdqggithldraeqarilqsrevprhlccrnpewlfriyqdtrvdipslmqsirrvvkgrpgprrq

rwaislypgkvrearcqasvqgatearlsvswqipwnlkylkvrevryevwlqeqgentyvpymltlqnhtftenikpf

ttylvwvrcifnrsllgpfadvlvcst

>Ss_XP_003132189_AGO61like.pro

MHLSAVLNALLVSVLAAVLWKHVRLREHAASLEEELVLGRRAPEPAPTLRIDYPKALQILTEG GTHMVCTGRTHTDRIC

RFKWLCYSSEAEEFIFFHGNASVMLPSLGSRRFQPALLDLSTVEDHNTQYFNFVELPAAALRFM PKPVFVPDVALIANR

FNPDNLMHVFHDDLLPLFYTLRQFPGLAREARLFFMEGWGEGAHFDLYKLLSPKQPLLRAQL KALGRLLCFSHAFVGLS

KVTTWYQYGFVQPQGPKANILVSGNEIRQFARFLTEKLNVSHAGGALGEEYILVFSRTQNRLIL NEAELLLALAQEFQM

KTVTVSLEDHAFADVVRLVSNASMLVSMHGAQLVTALFLPRGAAVVELFPYAVNPDHYTPYKT LATLPGMDLQYIAWRN

TMPENTVTHPERPWDQGGIAHLDRAEQARILQSQEVPRHLCCRNPEWLFRIYQDTKVDIPSLIQ TIRRVVKGRPGPRKQ

KWTVSLYPGKVREARCQSSVQGASEARLTVSWQIPWNLKYLKVREVKYEVWLQEQGENTYVP YILTLQNHTFTENIKPF

TTYLVWVRCIFNKTLLGPFADVLVCNT

>Bt_Q5NDF2_AGO61.pro LOCUS AGO61_BOVIN 580 aa linear MAM 19-OCT-2011

mhlsavlnallvsvlaavlwkhvrlrehaasleeelavgrraadpapalridypkalqilteggthmvctgrthtdrlc

rfkwlcysseaeefiffhgnasvmlpslgsrrfqpalldlstvedhntqyfnfvelpaaalrfmpkpvfvpdvalianr

fnpdnlmhvfhddllplfytlrqfpglarearlffmegwgegahfdlykllspkqpllraqlkalgrllcfshafvgls

kvttwyqygfvqpqgpkanilvsgneirqfahflmeklnvsqaggplgeeyilvfsrtqnrlilneaelllalaqefqm

ktvtvsledhafadvvrlvsnasmlvsmhgaqlvtalflprgaavvelfpyavnpdhytpyktlatlpgmdlqyiawqn

tmpentvthperpwdqggiahldraeqarilqsrevprhlccrnpewlfriyqdtkvdipsliqtirrvvkghpgprkq

kwtvslypgkvrearcqasvqgasearlsvswqipwnlkylkvrevkyevwlqeqgentyvpymlalqnhtftenikpf

ttylvwircifnktllgpfadvlvcst

>xenopus_tropicalis_Q5NDE6_AGO61.pro LOCUS AGO61_XENTR 576 aa linear VRT 19-OCT-2011

mnisavfnallvsimaavlwkhvklleqfyvieeeleltrqsqelsqvridyqaalqalvedgtrmvcsgrmhtdrvcr

feslcysteaeefvffhsnssimlpnlgprrfqpalldlssvddhntqyfnfielpaaalkfmpkpvfvpdvalimnrf

npdnlmhvfhddlipifytiqqfadldfesrlffmegwneglhfelykfmsnkqpllkeqlktlgrllcftksyvglsk

ittwyqygfvqpqgpkanilvsgneirhfakfmmgklnitkdqnaaeayivlfsrsmnrlivneaelllalaqefqmkt

itvsledhsfadivrlisnatmlvsmhgaqlitslflpkgaivvelfpygvnpehytpyktlstlpgmelqyvawqnte

eentiaypnrpweqggivhldkteqerikkskevprhlccrnpewlfriyqdtkvnissliqviksktklgsrrqkwtq

glypgkvreskcqasaqgtgeaklfvswqipwnlkflkvrdvkyevwiqeqgensympyilsqqnytfsenikplttyl

vwircifnktllgpfaevlvcnt

>Xenopus_laevis_Q5NDE7_AGO61.pro

MNISAVFSALLVSIMAAVLWKHVKLLDQFYVIEEELELTRQSQELSQVRIDYQAALQALVEDGTRMVCSGRMHTDRVCR

FESLCYSTEAEEFVFFHSNASIMLPNLGPRRFQPALLDLSSVDDHNTQYFNFIELPAAALKFMPKPVFVPDVALIMNRF

NPDNLMHVFHDDLLPIFYTIQQFPDLDFESRLFFMEGWNEGLHFELYKFMSNKQPLLKEQLKT

LGRLLCFTKSYVGLSK

ITTWYQYGFVQPQGPKANILVSGNEIRHFAKFMMGKLNITLDQNAAEAYIVLFSRSMNRLIVNEAELLLALAQEFQMKT

ITVSLEDHSFSDIVRLLSNATMLVSMHGAQLVTSLFLPKGAVVVELFPYGINPEHYTPYKTLSTLPGMELQYVAWQNTE

EENTITYPDRPWEQGGIVHLETKEQERIKKSKEVPRHLCCRNPEWLFRIYQDTKVNISSLIQVIKSTVKKKLGLRRQKW

TQGLYPGKVRESKCQASAQGTSEAKLFVSWQIPWNLKFLKVRDVKYEVWIQEQGENSYMPYILSQQNYTFSENIKPFTT

YLVWIRCIFNKTLLGPFAEVLVCST

>Gg_Q5NDE8_AGO61.pro LOCUS AGO61_CHICK 577 aa linear VRT 19-OCT-2011

mniaavfnallvsvlatvlwkyiklrehafmveeelvlmrqsqelsqvqidyhaalqtlledgtrmvctgrmhtdricr

feslcysteaeefiyfhsnssvmlpnlgsrrfqpalldlssvedhntqyfnfvelpaaalkfmpkpvfvpdvalianrf

npdnlmhvfhddllpiyytmqqftdldpetrlffmegwsegvhfdlykllsnkqpllreqlktlgrllcftksyvglsk

ittwyqygfvqpqgpkanilvsgneirqftkfmmqklnvsleessseeyivvfsrtinrlilneaelilalaqefqmkt

itvsleehsfsdivrlisnasmlvsmhgaqlvmslflprgatvvelfpyainpehytpyktlatlpgmdlqyiawqnta

redtvtypdrpwdqggiahldkaeqeriikstevprhlccrnpewlfrayqdtkvdipslihvirqtvkskpgpkkkws

gslypgkvrdarcqasvqgtsearlsvswqvpwnlkylkvrevkyevwiqeqgentympyilshqnhtfsenikpftiy

lvwircifnknllgpfadvllcst

>Dario_rerio_Q5NDE5_AGO61.pro LOCUS AGO61_DANRE 578 aa linear VRT 19-OCT-2011

mnlpavlngllvsvvaallwkyvrlvehtsqleeelqltrqsqefsqvridyhgallalqehgtrmvctgkmhtdricr

fdylcycteaeefvffhsnasvmlpnlgsrrfqpalldlssvedhntqyfnflelpaaalkfmpkpvfvpdvtlilnrf

npdnlmhifhddllpvyytmqqysdlddearlvfmegwgegahfdlyrllsskqpllkdqlktfgklmcftksyvglsk

mttwyqygfvqpqgpkanilisgneirqfasflmerlnitreeeeedddyivvfkrttnrlilneaelllalaqefqmr

tvtvsleeqsfdniiqiisraamlvsmhgaqmitsmflprgaavvelfpygvnpeqytpyktlaslpgmdlqyvawrnt

meentvtfpdrpwdqggivhlekeeqerilaskevprhlccrnpewlfriyqdttvdlasfldvlrdglkklnlkkakv

astvhpgrvrepkcqtsvqatneaklsvswqipwnlkylkvkevkyevwiqeqgentympyilphqnytfsenikpftt

ylvwvrcifnknllgpfadvlickt

>tetraodon_nigroviridis_Q5NDE3_AGO61.pro

MGVGTLLNGLLVSVVAALLWKYSKLSEHAALLEEELHMTRRSQELSQAHIDYHVALQALQEH GTRMVCTGKMHTDRICR

FDYLCYCSEAEEFVFFHSNSSVMLPNLGSRRFQPALLDLSSVEDHNTQYFNFLELPAATLRFLPK PVFVPDVALILNRF

NPDNLMHVFHDDLLPAFYTMKQFLDLDEDARLVFMEGWDEGPHFHLYRLLSDKQPLLKEQLR NFGKLMCFTKSYIGLSK

MTTWYQYGFVQPQGPKANILVSGNEIRHFAKVLMEKMNVTRAEGGQEDEYIVVFSRSSTRLIL NQAELVMALAQEFQMR

VVTVSLEEQSFASIVQVIGAASMLVSMHGAQLITALFLPPGAVVVELFPFAVNPDQYTPYRTLAA LPGMDLHYISWRNT

EEENTITHPDRPWEQGGIAHLEKEEQERIVASKDVPRHLCCRNPEWLFRIYQDTFVDIPSFLEAL QAGLKAKPVWKKSK

LSGGLHPGRVRDARCQTSVQTSSEAKLTVSWQMPWNLKYLKVREVKYEVWIQEQGENTYMP YILPQQNYTFSDNIKPFT

TYLVWVRCIFNKNLLGPFADVLMCRT

>takifugu_rubripes_Q5NDE4_AGO61.pro

MSVGTLLNGLLVSIVAALLWKYSKLSEHAALLEEELHMTRQSQELSQAHIDYHVALQALQEHG TRMVCTGKMHTDRICR

FDYLCYCSEAEEFVFFHSNSSVMLPNLGSRRFQPALLDLSSVEDHNTQYFNFLELPAATLRFMPK PVFVPDVTLILNRF

NPDNLMHVFHDDLLPAFYTMKQFLDSDEDARLVFMEGWEEGPHFELYRLLSNKQPLLKEQLR NFGKLMCFTKSYIGLSK

MTTWYQYGFVQPQGPKANILVSGNEIRHFAKVLMEKMNITRAAGGEKDQGNAEDEKPKDEYI VVFSRSTTRLILNEAEL

IMALAQEFQMRVVTVSLEEQSFPSIVQVISGASMLVSMHGAQLITSLFLPPGAVVVELYPFAVNP DQYTPYRTLASLPG

MDLHYIPWRNTEEENTVTHPDRPWEQGGIAHLEKEEQEQIMASKDVPRHLCCRNPEWLFRIYQ

DTLVDIPSFLEVLQEG

VKAKPLLKKSKLSSTLHPGRVRDPQCQTSVQTSNEAKLTVSWQIPWNLKYLKVREVKYEVWIQ EQGENTYMPYILPQQN

YTFSDNIKPFTTYLVWVRCIFNKNLLGPFADVLMCRT

>eqquus_caballus_XP_001493422_GT41.pro

MASSVGNVADSTEPTKRMLSFQGLAELAHREYQAGDFEAAERHCMQLWRQEPDNTGVLLLLS SIHFQCRRLDRSAHFST

LAIKQNPLLAEAYSNLGNVYKERGQLQEAIEHYRHALRLKPDFIDGYINLAAALVAAGDMEGA VQAYVSALQYNPDLYC

VRSDLGNLLKALGRLEEAKACYLKAIETQPNFAVAWSNLGCVFNAQGEIWLAIHHFEKAVTLD PNFLDAYINLGNVLKE

ARIFDRAVAAYLRALSLSPNHAVVHGNLACVYYEQGLIDLAIDTYRRAIELQPHFPDAYCNLAN ALKEKGSVAEAEDCY

NTALRLCPTHADSLNNLANIKREQGNIEEAVRLYRKALEVFPEFAAAHSNLASVLQQQGKLQEA LMHYKEAIRISPTFA

DAYSNMGNTLKEMQDVQGALQCYTRAIQINPAFADAHSNLASIHKDSGNIPEAIASYRTALKLK PDFPDAYCNLAHCLQ

IVCDWTDYDERMKKLVSIVADQLEKNRLPSVHPHHSMLYPLSHGFRKAIAERHGNLCLDKINV LHKPPYEHPKDLKLSD

GRLRVGYVSSDFGNHPTSHLMQSIPGMHNPDKFEVFCYALSPDDGTNFRVKVMAEANHFIDLS QIPCNGKAADRIHQDG

IHILVNMNGYTKGARNELFALRPAPIQAMWLGYPGTSGALFMDYIITDQETSPAEVAEQYSEKL AYMPHTFFIGDHANM

FPHLKKKAVIDFKSNGHIYDNRIVLNGIDLKAFLDSLPDVKIVKMKCPDGGDNADSSNTALNMP VIPMNTIAEAVIEMI

NRGQIQITINGFSISNGLATTQINNKAATGEEVPRTIIVTTRSQYGLPEDAIVYCNFNQLYKIDPST LQMWANILKRVP

NSVLWLLRFPAVGEPNIQQYAQNMGLPQNRIIFSPVAPKEEHVRRGQLADVCLDTPLCNGHTT GMDVLWAGTPMVTMPG

ETLASRVAASQLTCLGCLELIAKNRQEYEDIAVKLGTDLEYLKKIRGKVWKQRISSPLFNTKQY TMELERLYLQMWEHY

AAGNKPDHMIKPVEVTESA

>Ss_Q27HV0_GT41_OGT.pro

MASSVGNVADSTEPTKRMLSFQGLAELAHREYQAGDFEAAERHCMQLWRQEPDNTGVLLLLS SIHFQCRRLDRSAHFST

LAIKQNPLLAEAYSNLGNVYKERGQLQEAIEHYRHALRLKPDFIDGYINLAAALVAAGDMEGA VQAYVSALQYNPDLYC

VRSDLGNLLKALGRLEEAKACYLKAIETQPNFAVAWSNLGCVFNAQGEIWLAIHHFEKAVTLD PNFLDAYINLGNVLKE

ARIFDRAVAAYLRALSLSPNHAVVHGNLACVYYEQGLIDLAIDTYRRAIELQPHFPDAYCNLAN ALKEKGSVAEAEDCY

NTALRLCPTHADSLNNLANIKREQGNIEEAVRLYRKALEVFPEFAAAHSNLASVLQQQGKLQEA LMHYKEAIRISPTFA

DAYSNMGNTLKEMQDVQGALQCYTRAIQINPAFADAHSNLASIHKDSGNIPEAIASYRTALKLK PDFPDAYCNLAHCLQ

IVCDWTDYDERMKKLVSIVADQLEKNRLPSVHPHHSMLYPLSHGFRKAIAERHGNLCLDKINV LHKPPYEHPKDLKLSD

GRLRVGYVSSDFGNHPTSHLMQSIPGMHNPDKFEVFCYALSPDDGTNFRVKVMAEANHFIDLS QIPCNGKAADRIHQDG

IHILVNMNGYTKGARNELFALRPAPIQAMWLGYPGTSGALFMDYIITDQETSPAEVAEQYSEKL AYMPHTFFIGDHANM

FPHLKKKAVIDFKSNGHIYDNRIVLNGIDLKAFLDSLPDVKIVKMKCPDGGDNADSSNTALNMP VIPMNTIAEAVIEMI

NRGQIQITINGFSISNGLATTQINNKAATGEEVPRTIIVTTRSQYGLPEDAIVYCNFNQLYKIDPST LQMWANILKRVP

NSVLWLLRFPAVGEPNIQQYAQNMGLPQNRIIFSPVAPKEEHVRRGQLADVCLDTPLCNGHTT GMDVLWAGTPMVTMPG

ETLASRVAASQLTCLGCLELIAKNRQEFEDIAVKLGTDLEYLKKIRGKVWKQRISSPLFNTKQY TMELERLYLQMWEHY

AAGNKPDHMIKPVEVTESA

>Hs_O15294_3_GT41_OGTlike.pro

MASSVGNVADSTEPTKRMLSFQGLAELAHREYQAGDFEAAERHCMQLWRQEPDNTGVLLLLS

SIHFQCRRLDRSAHFST

LAIKQNPLLAEAYSNLGNVYKERGQLQEAIEHYRHALRLKPDFIDGYINLAAALVAAGDMEGA VQAYVSALQYNPDLYC

VRSDLGNLLKALGRLEEAKACYLKAIETQPNFAVAWSNLGCVFNAQGEIWLAIHHFEKAVTLD PNFLDAYINLGNVLKE

ARIFDRAVAAYLRALSLSPNHAVVHGNLACVYYEQGLIDLAIDTYRRAIELQPHFPDAYCNLAN ALKEKGSVAEAEDCY

NTALRLCPTHADSLNNLANIKREQGNIEEAVRLYRKALEVFPEFAAAHSNLASVLQQQGKLQEA LMHYKEAIRISPTFA

DAYSNMGNTLKEMQDVQGALQCYTRAIQINPAFADAHSNLASIHKDSGNIPEAIASYRTALKLK PDFPDAYCNLAHCLQ

IVCDWTDYDERMKKLVSIVADQLEKNRLPSVHPHHSMLYPLSHGFRKAIAERHGNLCLDKINV LHKPPYEHPKDLKLSD

GRLRVGYVSSDFGNHPTSHLMQSIPGMHNPDKFEVFCYALSPDDGTNFRVKVMAEANHFIDLS QIPCNGKAADRIHQDG

IHILVNMNGYTKGARNELFALRPAPIQAMWLGYPGTSGALFMDYIITDQETSPAEVAEQYSEKL AYMPHTFFIGDHANM

FPHLKKKAVIDFKSNGHIYDNRIVLNGIDLKAFLDSLPDVKIVKMKCPDGGDNADSSNTALNMP VIPMNTIAEAVIEMI

NRGQIQITINGFSISNGLATTQINNKAATGEEVPRTIIVTTRSQYGLPEDAIVYCNFNQLYKIDPST LQMWANILKRVP

NSVLWLLRFPAVGEPNIQQYAQNMGLPQNRIIFSPVAPKEEHVRRGQLADVCLDTPLCNGHTT GMDVLWAGTPMVTMPG

ETLASRVAASQLTCLGCLELIAKNRQEYEDIAVKLGTDLEYLKKVRGKVWKQRISSPLFNTKQY TMELERLYLQMWEHY

AAGNKPDHMIKPVEVTESA

>Hs_Q548W1_GT41_OGT.pro

MLQGHFWLVREGIMISPSSPPPPNLFFFPLQIFPFPFTSFPSHLLSLTPPKACYLKAIETQPNFAVA WSNLGCVFNAQG

EIWLAIHHFEKAVTLDPNFLDAYINLGNVLKEARIFDRAVAAYLRALSLSPNHAVVHGNLACVY YEQGLIDLAIDTYRR

AIELQPHFPDAYCNLANALKEKGSVAEAEDCYNTALRLCPTHADSLNNLANIKREQGNIEEAVR

LYRKALEVFPEFAAA

HSNLASVLQQQGKLQEALMHYKEAIRISPTFADAYSNMGNTLKEMQDVQGALQCYTRAIQINPAFADAHSNLASIHKDS

GNIPEAIASYRTALKLKPDFPDAYCNLAHCLQIVCDWTDYDERMKKLVSIVADQLEKNRLPSVHPHHSMLYPLSHGFRK

AIAERHGNLCLDKINVLHKPPYEHPKDLKLSDGRLRVGYVSSDFGNHPTSHLMQSIPGMHNPDKFEVFCYALSPDDGTN

FRVKVMAEANHFIDLSQIPCNGKAADRIHQDGIHILVNMNGYTKGARNELFALRPAPIQAMWLGYPGTSGALFMDYIIT

DQETSPAEVAEQYSEKLAYMPHTFFIGDHANMFPHLKKKAVIDFKSNGHIYDNRIVLNGIDLKAFLDSLPDVKIVKMKC

PDGGDNADSSNTALNMPVIPMNTIAEAVIEMINRGQIQITINGFSISNGLATTQINNKAATGEEVPRTIIVTTRSQYGL

PEDAIVYCNFNQLYKIDPSTLQMWANILKRVPNSVLWLLRFPAVGEPNIQQYAQNMGLPQNRIIFSPVAPKEEHVRRGQ

LADVCLDTPLCNGHTTGMDVLWAGTPMVTMPGETLASRVAASQLTCLGCLELIAKNRQEYEDIAVKLGTDLEYLKKVRG

KVWKQRISSPLFNTKQYTMELERLYLQMWEHYAAGNKPDHMIKPVEVTESA

>eqquus_caballus_XP_001493438_GT41.pro

MASSVGNVADSTGLAELAHREYQAGDFEAAERHCMQLWRQEPDNTGVLLLLSSIHFQCRRLDRSAHFSTLAIKQNPLLA

EAYSNLGNVYKERGQLQEAIEHYRHALRLKPDFIDGYINLAAALVAAGDMEGAVQAYVSALQYNPDLYCVRSDLGNLLK

ALGRLEEAKACYLKAIETQPNFAVAWSNLGCVFNAQGEIWLAIHHFEKAVTLDPNFLDAYINLGNVLKEARIFDRAVAA

YLRALSLSPNHAVVHGNLACVYYEQGLIDLAIDTYRRAIELQPHFPDAYCNLANALKEKGSVAEAEDCYNTALRLCPTH

ADSLNNLANIKREQGNIEEAVRLYRKALEVFPEFAAAHSNLASVLQQQGKLQEALMHYKEAIRISPTFADAYSNMGNTL

KEMQDVQGALQCYTRAIQINPAFADAHSNLASIHKDSGNIPEAIASYRTALKLKPDFPDAYCNLAHCLQIVCDWTDYDE

RMKKLVSIVADQLEKNRLPSVHPHHSMLYPLSHGFRKAIAERHGNLCLDKINVLHKPPYEHPK

DLKLSDGRLRVGYVSS

DFGNHPTSHLMQSIPGMHNPDKFEVFCYALSPDDGTNFRVKVMAEANHFIDLSQIPCNGKAAD RIHQDGIHILVNMNGY

TKGARNELFALRPAPIQAMWLGYPGTSGALFMDYIITDQETSPAEVAEQYSEKLAYMPHTFFIG DHANMFPHLKKKAVI

DFKSNGHIYDNRIVLNGIDLKAFLDSLPDVKIVKMKCPDGGDNADSSNTALNMPVIPMNTIAEAV IEMINRGQIQITIN

GFSISNGLATTQINNKAATGEEVPRTIIVTTRSQYGLPEDAIVYCNFNQLYKIDPSTLQMWANILK RVPNSVLWLLRFP

AVGEPNIQQYAQNMGLPQNRIIFSPVAPKEEHVRRGQLADVCLDTPLCNGHTTGMDVLWAGTP MVTMPGETLASRVAAS

QLTCLGCLELIAKNRQEYEDIAVKLGTDLEYLKKIRGKVWKQRISSPLFNTKQYTMELERLYL QMWEHYAAGNKPDHMI

KPVEVTESA

>Bt_A5D7G1_GT41.pro

MASSVGNVADSTGLAELAHREYQAGDFEAAERHCMQLWRQEPDNTGVLLLLSSIHFQCRRLD RSAHFSTLAIKQNPLLA

EAYSNLGNVYKERGQLQEAIEHYRHALRLKPDFIDGYINLAAALVAAGDMEGAVQAYVSALQY NPDLYCVRSDLGNLLK

ALGRLEEAKACYLKAIETQPNFAVAWSNLGCVFNAQGEIWLAIHHFEKAVTLDPNFLDAYINLG NVLKEARIFDRAVAA

YLRALSLSPNHAVVHGNLACVYYEQGLIDLAIDTYRRAIELQPHFPDAYCNLANALKEKGSVAE AEDCYNTALRLCPTH

ADSLNNLANIKREQGNIEEAVRLYRKALEVFPEFAAAHSNLASVLQQQGKLQEALMHYKEAIRI SPTFADAYSNMGNTL

KEMQDVQGALQCYTRAIQINPAFADAHSNLASIHKDSGNIPEAIASYRTALKLKPDFPDAYCNLA HCLQIVCDWTDYDE

RMKKLVSIVADQLEKNRLPSVHPHHSMLYPLSHGFRKAIAERHGNLCLDKINVLHKPPYEHPK DLKLSDGRLRVGYVSS

DFGNHPTSHLMQSIPGMHNPDKFEVFCYALSPDDGTNFRVKVMAEANHFIDLSQIPCNGKAAD RIHQDGIHILVNMNGY

TKGARNELFALRPAPIQAMWLGYPGTSGALFMDYIITDQETSPAEVAEQYSEKLAYMPHTFFIG

DHANMFPHLKKKAVI

DFKSNGHIYDNRIVLNGIDLKAFLDSLPDVKIVKMKCPDGGDNVDSSNTALNMPVIPMNTIAEAV IEMINRGQIQITIN

GFSISNGLATTQINNKAATGEEVPRTIIVTTRSQYGLPEDAIVYCNFNQLYKIDPSTLQMWANILK RVPNSVLWLLRFP

AVGEPNIQQYAQNMGLPQNRIIFSPVAPKEEHVRRGQLADVCLDTPLCNGHTTGMDVLWAGTP MVTMPGETLASRVAAS

QLTCLGCLELIAKNRQEYEDIAVKLGTDLEYLKKIRGKVWKQRISSPLFNTKQYTMELERLYL QMWEHYAAGNKPDHMI

KPVEVTESA

>Mm_P56558_GT41_OGT.pro

MASSVGNVADSTGLAELAHREYQAGDFEAAERHCMQLWRQEPDNTGVLLLLSSIHFQCRRLD RSAHFSTLAIKQNPLLA

EAYSNLGNVYKERGQLQEAIEHYRHALRLKPDFIDGYINLAAALVAAGDMEGAVQAYVSALQY NPDLYCVRSDLGNLLK

ALGRLEEAKACYLKAIETQPNFAVAWSNLGCVFNAQGEIWLAIHHFEKAVTLDPNFLDAYINLG NVLKEARIFDRAVAA

YLRALSLSPNHAVVHGNLACVYYEQGLIDLAIDTYRRAIELQPHFPDAYCNLANALKEKGSVAE AEDCYNTALRLCPTH

ADSLNNLANIKREQGNIEEAVRLYRKALEVFPEFAAAHSNLASVLQQQGKLQEALMHYKEAIRI SPTFADAYSNMGNTL

KEMQDVQGALQCYTRAIQINPAFADAHSNLASIHKDSGNIPEAIASYRTALKLKPDFPDAYCNLA HCLQIVCDWTDYDE

RMKKLVSIVAEQLEKNRLPSVHPHHSMLYPLSHGFRKAIAERHGNLCLDKINVLHKPPYEHPK DLKLSDGRLRVGYVSS

DFGNHPTSHLMQSIPGMHNPDKFEVFCYALSPDDGTNFRVKVMAEANHFIDLSQIPCNGKAAD RIHQDGIHILVNMNGY

TKGARNELFALRPAPIQAMWLGYPGTSGALFMDYIITDQETSPAEVAEQYSEKLAYMPHTFFIG DHANMFPHLKKKAVI

DFKSNGHIYDNRIVLNGIDLKAFLDSLPDVKIVKMKCPDGGDNADTTNTALNMPVIPMNTIAEA VIEMINRGQIQITIN

GFSISNGLATTQINNKAATGEEVPRTIIVTTRSQYGLPEDAIVYCNFNQLYKIDPSTLQMGANILK

RVPNSVLWLLRFP

AVGEPNIQQYAQNMGLPQNRIIFSPVAPKEEHVRRGQLADVCLDTPLCNGHTTGMDVLWAGTPMVTMPGETLASRVAAS

QLTCLGCLELIAKSRQEYEDIAVKLGTDLEYLKKIRGKVWKQRISSPLFNTKQYTMELERLYLQMWEHYAAGNKPDHMI

KPVEVTESA

>Gg_XP_003641145_GT41_OGT.pro

MATSVGNVADSTEPTKRMLSFQGLAELAHREYQAGDFEAAERHCMQLWRQEPDNTGVLLLLSSIHFQCRRLDRSAHFST

LAIKQNPLLAEAYSNLGNVYKERGQLQEAIEHYRHALRLKPDFIDGYINLAAALVAAGDMEGAVQAYVSALQYNPDLYC

VRSDLGNLLKALGRLEEAKACYLKAIETQPNFAVAWSNLGCVFNAQGEIWLAIHHFEKAVTLDPNFLDAYINLGNVLKE

ARIFDRAVAAYLRALSLSPNHAVVHGNLACVYYEQGLIDLAIDTYRRAIELQPHFPDAYCNLANALKEKGSVAEAEECY

NTALRLCPTHADSLNNLANIKREQGNIEEAVRLYRKALEVFPEFAAAHSNLASVLQQQGKLQEALMHYKEAIRISPTFA

DAYSNMGNTLKEMQDVQGALQCYTRAIQINPAFADAHSNLASIHKDSGNIPEAIASYRTALKLKPDFPDAYCNLAHCLQ

IVCDWTDYDERMKKLVSIVADQLEKNRLPSVHPHHSMLYPLSHSFRKAIAERHGNLCLDKINVLHKPPYEHPKDLKASE

GRLRIGYVSSDFGNHPTSHLMQSIPGMHNPDKFEVFCYALSPDDGTNFRVKVMAEANHFVDLSQIPCNGKAADRIHQDG

IHILINMNGYTKGARNELFALRPAPIQAMWLGYPGTSGALFMDYIITDKETSPVEVAEQYSEKLAYMPNTFFIGDHANM

FPHLKKKAVIDFKSNGHIYDNRIVLNGIDLKAFLDSLPDVKIVKMKCPDSGDSADSNAALSMPVIPMNTIAEAVIEMIN

RGQIQITINGFNISNGLATTQINNKAATGEEVPRTIIVTTRSQYGLPEDAVVYCNFNQLYKIDPSTLQMWANILKRVPN

SVLWLLRFPAVGEPNIQQYAQNLGLSQNRIIFSPVAPKEEHVRRGQLADVCLDTPLCNGHTTGMDVLWAGTPMVTMPGE

TLASRVAASQLTCLGCLELIAKSRQEYEDIAVKLGTDLEYLKKIRGKVWKQRISSPLFNTKQYT

MDLERLYLQMWDHYA

AGNKPDHMIKPVEASESA

>Xenopus_laevis_NP_001087833_GT41.pro

MAASVGNVADSTEPTKRMLSFQGLAELAHREYQAGDFEAAERHCMQLWRQEPDNTGVLLLLS SIHFQCRRLDRSAHFST

LAIKQNPLLAEAYSNLGNVYKERGQLQEAIEHYRHALRLKPDFIDGYINLAAALVAAGDMEGA VQAYVSALQYNPDLYC

VRSDLGNLLKALGRLEEAKACYLKAIETQPNFAVAWSNLGCVFNAQGEIWLAIHHFEKAVTLD PNFLDAYINLGNVLKE

ARIFDRAVAAYLRALSLSPNHAVVHGNLACVYYEQGLIDLAIDTYRRAIELQPHFPDAYCNLAN ALKEKGSVVDAEECY

NTALRLCPTHADSLNNLANIKREQGNIEEAVRLYRKALEVFPEFAAAHSNLASVLQQQGKLQEA LMHYKEAIRISPTFA

DAYSNMGNTLKEMQDVQGALQCYTRAIQINPAFADAHSNLASIHKDSGNIPEAIASYRTALKLK PDFPDAYCNLAHCLQ

IVCDWTDYDERMKKLVSIVAEQLEKNRLPSVHPHHSMLYPLSHAFRKAIAERHGNLCLDKINV LHKPPYEHPKDLKASD

GRLRVGYVSSDFGNHPTSHLMQSIPGMHNPDKFEVFCYALSPDDGTNFRVKVMAEANHFVDLS QIPCNGKAADRIHQDG

IHILVNMNGYTKGARNELFALKAAPIQAMWLGYPGTSGAPFMDYIISDKETSPIDVAEQYSEKL AYMPNTFFIGDHANM

FPHLKKKAVIDFKSNGHIYDNRIVLNGIDLKAFLESLPDVKIVKMKGPDSGDNVDNNSALSMPVI PMSSIAEAVIEMIN

RGQIQITINGFNISNGLATTQINNKAATGEEVPRTIIVTTRSQYGLPEDAVVYCNFNQLYKIDPST LQMWANILKRVPN

SVLWLLRFPAVGEPNIQQYAQNMGLPQSRIIFSPVAPKEEHVRRGQLADVCLDTPLCNGHTTG MDVLWAGTPMVTMPGK

ESAESLGLHNKDCFLSGDTLASRVAASQLTCLGCPELIAKSRQDYEDIAVKLGTDLEYLKKIRA KVWKQRISSPLFNTK

QYTMDLERLYLEMWEHFAAGNKRDHLIKPVESTESA

>xenopus_tropicalis_NP_001019747_GT41.pr

MAASVGSVADSTGLAELAHREYQAGDFEAAERHCMQLWRQEPDNTGVLLLLSSIHFQCRRLD RSAHFSTLAIKQNPLLA

EAYSNLGNVYKERGQLQEAIEHYRHALRLKPDFIDGYINLAAALVAAGDMEGAVQAYVSALQY NPDLYCVRSDLGNLLK

ALGRLEEAKACYLKAIETQPNFAVAWSNLGCVFNAQGEIWLAIHHFEKAVTLDPNFLDAYINLG NVLKEARIFDRAVAA

YLRALSLSPNHAVVHGNLACVYYEQGLIDLAIDTYRRAIELQPHFPDAYCNLANALKEKGSVVD AEECYNTALRLCPTH

ADSLNNLANIKREQGNIEEAVRLYRKALEVFPEFAAAHSNLASVLQQQGKLQEALMHYKEAIRI SPTFADAYSNMGNTL

KEMQDVQGALQCYTRAIQINPAFADAHSNLASIHKDSGNIPEAIASYRTALKLKPDFPDAYCNLA HCLQIVCDWTDYDE

RMKKLVSIVADQLEKNRLPSVHPHHSMLYPLSHGFRKAIAERHGNLCLDKINVLHKPPYEHPK DLKASDGRLRVGYVSS

DFGNHPTSHLMQSIPGMHNPDKFEVFCYALSPDDGTNFRVKVMAEANHFVDLSQIPCNGKAAD RIHQDGVHILINMNGY

TKGARNELFALKAAPIQAMWLGYPGTSGASFMDYIISDIETSPVDVAEQYSEKLAYMPNTFFIGD HANMFPHLKKKAVI

DFKSNGHIYDNRIVLNGIDLKAFLESLPDVKIVKMKGPDSGDNVDNNSALNMPVIPMSSIAEAVI EMINRGQIQITING

FNISNGLATTQINNKAATGEEVPRTIIVTTRSQYGLPEDAVVYCNFNQLYKIDPSTLQMWANILK RVPNSVLWLLRFPA

VGEPNIQQYAQNMGLPQSRIIFSPVAPKEEHVRRGQLADVCLDTPLCNGHTTGMDVLWAGTP MVTMPGDTLASRVAASQ

LTCLGCPELIAKGRQDYEDTAVKLGTDLEYLKKIRSKVWKQRISSPLFNTKQYTIDLERLYLQM WEHYSAGNKTDHLIK

TVESTESA

>Dario_rerio_B3DKP3_GT41.pro

MASSVGNVADSTEPTKRVLSFQGLAELAHREYQSGDFEAAERHCMQLWRQEPDNTGVLLLLSS IHFQCRRLDRSAHFST

LAIKQNPMLAEAYSNLGNVYKERGQLQEAIEHYRHALRLKPDFIDGYINLAAALVAAGDMEGA VQAYVSALQYNPDLYC

VRSDLGNLLKALGRLEEAKACYLKAIETQPNFAVAWSNLGCVFNAQGEIWLAIHHFEKAVTLD PNFLDAYINLGNVLKE

ARIFDRAVAGYLRALSLSPNHAVVHGNLACVYYEQGLIDLAIDTYRRAIELQPHFPDAYCNLAN ALKEKGNVSEAEECY

NTALRLCPTHADSLNNLANIKREQGNIEEAVQLYRKALEVFPEFAAAHSNLASVLQQQGKLQEA LMHYKEAIRISPTFA

DAYSNMGNTLKEMQDVQGALQCYTRAIQINPAFADAHSNLASIHKDSGNIPEAIASYRTALKLK PDFPDAYCNLAHCLQ

IVCDWTDYDERMKKLVSIVADQLEKNRLPSVHPHHSMLYPLSHGFRKAIAERHGNLCLDKINA LHKPAYEHPKDLKASS

GRLRVGYISSDFGNHPTSHLMQSIPGMHNSEKFEVFCYALSPDDGTNFRVKVMAEAHHFIDLSQ IPCNGKAADRIHQDG

IHILVNMNGYTKGARNELFALRPAPIQAMWLGYPGTSGAPFMDYIVSDKATSPIEVAEQYSEKL AYMPNTFFIGDHANM

FPHLKKKAVIDFKSNGHIFDNRIVLNGIDLKAFLESLPDVKVVKMECDGQEVADSNGALSMPIIP MNTAAEAIINMINQ

GQIQVTINGFTVSNGLATTQINNKAATGEEVPRTIVVTTRSQYGLPEDSIVYCNFNQLYKIDPPTL QMWANILKRVPNS

VIWLLRFPAVGEPNIQQYAQNLGLPASRIIFSPVAPKEEHVRRGQLADVCLDTPLCNGHTTGMD VLWAGTPMVTMPGET

LASRVAASQLTCLGCPELIAQSRQEYEDVAVKLGTDMEFLKKVRARVWKQRICSPLFNTKQYT MDLEKLYLQMWENHAS

GGKPDHLVKMQSLETSEST

>tetraodon_nigroviridis_CAF99103_GT41.pr

MATSVGNVADSTGLAELAHREYQSGDFEAAERHCMQLWRQEPDNTAMLATRDDFFYTLAIKQ NPLLAEAYSNLGNVYKE

RGQLQEAIEHYRHALRLKPDFIDGYINLAAALVAAGDMEGAVQAYVSALQYNPDLYCVRSDLG NLLKALGRLEEAKPAA

ATSLTKSKNMACYLKAIETQPNFAVAWSNLGCVFNAQGEIWLAIHHFEKAVTLDPNFLDAYINL GNVLKEARIFDRAVA

GYLRALSLSPNHAVVHGNLACVYYEQGLIDLAIDTYRRAIELQPHFPDAYCNLANALKEKGNVS EAEECYNTALRLCPT

HADSLNNLANIKREQGNIEEAIQLYRKALEVFPEFAAAHSNLASVLQQQGKLQEALMHYKEAIR ISPTFADAYSNMGNT

LKEMQDVQGALQCYTRAIQINPAFADAHSNLASIHKDSGNIPEAIASYRTALKLKPDFPDAYCNL AHCLQIVCDWTDYD

ERMKKLVTIVADQLDKNRLPSVHPHHSMLYPLSHGFRKAIAERHGNLCLDKINALHKPPYEHP KDLKASGGRLRVGYVS

SDFGNHPTSHLMQSIPGMHNPEKFEVFCYALSPDDSTNFRVKVVAEAHHFVDLSQISCNGKAAD RIHQDGVHILVNMNG

YTKGARNELFALRPAPVQAMWLGYPGTSGAPFMDYIISDKETSPIEVAEQYSEKLAYMPHTFFI GDHANMFPHLKKKAV

IDFKSNGHIFDNRIVLNGIDLKAFLDSLPDVKVIKMKCDNNQEPSADTNGALSMPVIPMNTAAEA IINMINQGQIQVTI

NGFTVSNGLATTQITNKAATGEEVLRTVVVTTRSQYGLPEDAIVYCNFNQLYKIDPPTLQMWA NILTRVPNSVLWLLRF

PAVGEPNIQQYAQKVGLPASRIIFSPVAPKEEHVRRGQLADVCLDTPLCNGHTTGMDVLWAGT PMVTMPGETLASRVAT

SQLSCLGCPELIAQSHEEYEDIAVKLGSDMEYLKMVRARVWKQRICSPLFNTKQYTMDLEKLY LRMWEHYSKGNKPEHL

VQTVEASENA

>anopheles_gambiae_XP_316319_GT41_OGT.pr

MSSVENAENGVGKYSYQTVYYIITNNNNNSTEEGESDQVENLHAITAAVDSELTRKRRRIEAIKM QGQIPGAAAVQVQG

GSPQAAALAAQQQQQQQSISVKMDPANQLSSAGLLELAHREYQAVDYDNAERHCMQLWRQ ESNNTGVLLLLSSIHFQC

RRLDKSAQFSTLAIKQNPLLAEAYSNLGNVYKERGQLQEALENYRHAVRLKPDFIDGYINLAAA LVAARDMEQAVQAYV

TALQYNPDLYCVRSDLGNLLKALGRLDEAKACYLKAIETRPDFAVAWSNLGCVFNAQGEIWLA IHHFEKAVALDPNFLD

AYINLGNVLKEARIFDRAVAAYLRALNLSPNNAVVHGNLACVYYEQGLIDLAIDTYRRAIDLQH NFPDAYCNLANALKE

KGQVKEAEESYNIALRLCPNHADSLNNLANIKREQGYIEEATRLYLKALEVFPEFAAAHSNLAS VLQQQGKLNEALLHY

KEAIRIQPTFADAYSNMGNTLKEMQDVAGALQCYTRAIQINPAFADAHSNLASIHKDSGNIPEAI QSYRTALKLKPDFP

DAYCNLAHCLQIVCDWTDYEARMKKLVAIVADQLDKNRLPSVHPHHSMLYPLSHEFRKTIAAR HANLCLEKINVLHKPP

YKFGRELSGRLRIGYVSSDFGNHPTSHLMQSIPGLHDRNRVEIFCYALSPDDGTTFRGKISREAE HFIDLSQTPCNGKA

ADRIHADGIHILVNMNGYTKGARNEIFALRPAPVQVMWLGYPGTSGASFMDYIVTDAVTSPLSL ESQYSEKLAYMPHTY

FIGDHRQMFPHLKERVIVSNRANNSLADNVAVINATDLSPLVESTDVKTVREVVLANKPVEIQH KVAELPTTTPIETMI

ASGQVQTSLNGVVVQNGLATTQTNNKAATGEEVPQNIVVTTRQQYGLPDDAIVYCNFNQLYKI DPLTLQSWVTILKHVP

NSVLWLLRFPAVGEANIQATAQQMGIAAGRIIFSNVAAKEEHVRRGQLADVCLDTPLCNGHTTS MDVLWTGTPVVTLPA

ETLASRVAASQLATLGCPELIAKSRQEYQDIAIKLGTDREYLKAMRAKVWVARCESPLFDCKQ YAQGLEMLFYKMWERF

ARGERPDHISAKDK

>Aedes_aegypti_XP_001649477_GT41_OGT.pro

MFKAVAAYLRALNLSPYNAVVHGNLACVYYEQGLIDLAIDTYRRAIELQPNFPDAYCNLANAL KEKGQVQEAEDCYNTA

LRLCPNHADSLNNLANIKREQGYIEEATRLYLKALEVFPEFAAAHSNLASVLQQQGKLNEALLH YKEAIRIQPTFADAY

SNMGNTLKEMQDVAGALQCYTRAIQINPAFADAHSNLASIHKDSGNIPDAIQSYRTALKLKPDF PDAYCNLAHCLQIVC

DWTDYEARMKKLVAIVADQLEKNRLPSVHPHHSMLYPLSHDFRKAIAARHANLCLEKIHILHK PPYKFSRDMSQRLRIG

YVSSDFGNHPTSHLMQSIPGMHDRSRVEVFCYALSPDDGTTFRSKISREAEHFIELSQIPCNGKA ADRIHADGIHILVN

MNGYTKGARNEIFALRPAPIQVMWLGYPGTSGASFMDYIITDTVTSPMELADQYSEKLAYMPH TYFIGDHRQMFPHLKE

RLIVSGKQQNNQLVDNVAVINATDLSPLVENTDVKTVREVILAHKPVEIQHKVVELPTTTPVET MIASGQIQTSLNGVV

VQNGLATTQTNNKAATGEEVPQNIVVTTRQQYGLPDDAVVYCNFNQLYKIDPHTLASWVNILK HVPNSVLWLLRFPAVG

ETNIQAAAQQLGLSPGRIIFSNVAAKEEHVRRGQLADVCLDTPLCNGHTTSMDVLWTGTPVVT LPAETLASRVAASQLA

TLGCPELIARSRQEYQDIAIKLGTDKEYLKAIRAKVWLARCESPLFDCKQYAQGMEALFYKMW ERFARGEKPDHISAKE

GPK

>bombyx_mori_BGIBMGA011033_OGT1.pro

MQPQANVAVPQSVTTQPQAQQIVGVPANAVILKMSDLQQISTVGLLELAHREYQAGDYESAEH HCMQLWRQDSTNTGVL

LLLSSIHFQCRRLDKSAHFSTLAIKQNPLLAEAYSNLGNVYKERGQLQEALENYRHAVRLKPDFI DGYINLAAALVAAG

DMEQAVQAYVTALQYNPDLYCVRSDLGNLLKALGRLDEAKACYLKAIETRPDFAVAWSNLGC VFNAQSEIWLAIHHFEK

AVALDPNFLDAYINLGNVLKEARIFDRAVAAYLRALNLSPNNAVVHGNLACVYYEQGLIDLAID TYRRAIELQQNFPDA

YCNLANALKEKGQVTDAEECYNTALRLCPSHADSLNNLANIKREQGYIEEATRLYLKALEVFPE FAAAHSNLASVLQQQ

GKLNEALMHYKEAIRIQPTFADAYSNMGNTLKEMQDVAGALQCYTRAIQINPAFADAHSNLASI HKDSGNIPEAIQSYR

TALKLKPDFPDAYCNLAHCLQIVCDWTDYEARMKKLVSIVAEQLEKNRLPSVHPHHSMLYPLT HDFRKAIAARHANLCL

EKVQVLHKNPYKFPRDLQGRLRIGYVSSDFGNHPTSHLMQSVPGLHDRTKVEIFCYALSPDDGT TFRSKIAREAEHFID

LSQMPCNGKAADKIYGDGIHILVNMNGYTKGARNEIFALRPAPVQVMWLGYPGTSGASYMDY LVTDAVTSPVELASQYS

EKLAYMPHTYFVGDHKQMFPHLQERLILSDKVKSHNNLGSVADNVAVINATDLSPLVENTDIKE IKEIVRAARPVEISL

KVAELPTTTPIETMIASGQVQTSVNGVILQNGLATTQTNNKAATGEEVPQSIVITTRQQYGLPDD AVVYCNFNQLYKID

PLTLHMWVYILKHVPNSVLWLLRFPAVGEPNLQSTANQLGLPPGRIIFSNVAAKEEHVRRGQL ADVCLDTPLCNGHTTS

MDILWTGTPVVTLPGETLASRVAASQLNTLGCPELIARTRQEYQDIAVRLGTDREYLKAIRAKV WTARTDSPLFDCKAY

ATGLEMLYNRMWSRHARGDRPDHIQALEK

>Apis_mellifera_XP_003249419_GT41_OGT1.p

MVAIIMQAQQQPQQQQQQNHQQIAGTSVILKMNEIQQLSTVGLLELAHREYQAGDYENAERH CMQLWRQETNNTGVLLL

LSSIHFQCRRLEKSAHYSSLAIKQNPLLAEAYSNLGNVFKERGQLQEALENYRHAVRLKPDFIDG YINLAAALVAAGDM

EQAVQAYVTALQYNPDLYCVRSDLGNLLKALARLDEAKACYLKAIETRPDFAVAWSNLGCVFN AQGEIWLAIHHFEKAV

ALDPNFLDAYINLGNVLKEARIFDRAVAAYLRALNLSPNNAVVHGNLACVYYEQGLIDLAIDTY RRAIELQPNFPDAYC

NLANALKEKGQVVEAEDCYNTALRLCPSHADSLNNLANIKREQGYIEEATRLYLKALEVFPEFA AAHSNLASVLQQQGK

LNEALMHYKEAIRIQPTFADAYSNMGNTLKEMQDIQGALQCYTRAIQINPAFADAHSNLASIHK DSGNIPEAIQSYRTA

LKLKPDFPDAYCNLAHCLQIVCDWTDYEARMKKLVSIVAEQLDKNRLPSVHPHHSMLYPLSHE FRKAIAARHANLCIEK

IHVLHKQPYKYPREIGGRLRIGYVSSDFGNHPTSHLMQSIPGLHERQNVEIFCYALSADDGTTFR AKIARETEHFVDLS

QIPCNGKAADRINADGIHILVNMNGYTKGARNEIFALRPAPVQVMWLGYPGTSGASFMDYLITD EVTSPLELASQYSEK

LAYMPHTYFIGDHKQMFPHLKERLILTDKLNMKGKVADNVAVINATDLSPMIENTCVKEIREV VVPDAKNKPVEISLKV

AELPTTTPIETMIASGQCQMSVNGVVVQNGMATTQVNNKTATGEEVPQNIMITTRQQYGLPED AVVYCNFNQLYKIDPL

TLHMWAHILKHVPNSVLWLLRFPAVGEPNLQATAQQLGLAPGRILFSNVAAKEEHVRRGQLA DVCLDTPLCNGHTTSMD

VLWTGTPVVTLPGETLASRVAASQLNTLGCPELIARTRQEYQDIAIRLGTDREYLKATRAKVW KARSESPLFNCKLYAM

GMEMLYKKMWERYARGEKPDHVSAVDKNEREKLLTAAS

>Apis_mellifera_XP_XP_623820_GT41.pro

MSIRLAEKKENVIKNSSGIIGNVVNSSDDVCNGTVEWQNGQNLLVVTQDTQGKELIAIADNQTIN VAVDSYWLLELAHR

EYQAGDYENAERHCMQLWRQETNNTGVLLLLSSIHFQCRRLEKSAHYSSLAIKQNPLLAEAYS NLGNVFKERGQLQEAL

ENYRHAVRLKPDFIDGYINLAAALVAAGDMEQAVQAYVTALQYNPDLYCVRSDLGNLLKALAR LDEAKACYLKAIETRP

DFAVAWSNLGCVFNAQGEIWLAIHHFEKAVALDPNFLDAYINLGNVLKEARIFDRAVAAYLRA LNLSPNNAVVHGNLAC

VYYEQGLIDLAIDTYRRAIELQPNFPDAYCNLANALKEKGQVVEAEDCYNTALRLCPSHADSLN NLANIKREQGYIEEA

TRLYLKALEVFPEFAAAHSNLASVLQQQGKLNEALMHYKEAIRIQPTFADAYSNMGNTLKEMQ DIQGALQCYTRAIQIN

PAFADAHSNLASIHKDSGNIPEAIQSYRTALKLKPDFPDAYCNLAHCLQIVCDWTDYEARMKKL VSIVAEQLDKNRLPS

VHPHHSMLYPLSHEFRKAIAARHANLCIEKIHVLHKQPYKYPREIGGRLRIGYVSSDFGNHPTSH LMQSIPGLHERQNV

EIFCYALSADDGTTFRAKIARETEHFVDLSQIPCNGKAADRINADGIHILVNMNGYTKGARNEIF ALRPAPVQVMWLGY

PGTSGASFMDYLITDEVTSPLELASQYSEKLAYMPHTYFIGDHKQMFPHLKERLILTDKLNMKG KVADNVAVINATDLS

PMIENTCVKEIREVVVPDAKNKPVEISLKVAELPTTTPIETMIASGQCQMSVNGVVVQNGMATT QVNNKTATGEEVPQN

IMITTRQQYGLPEDAVVYCNFNQLYKIDPLTLHMWAHILKHVPNSVLWLLRFPAVGEPNLQAT AQQLGLAPGRILFSNV

AAKEEHVRRGQLADVCLDTPLCNGHTTSMDVLWTGTPVVTLPGETLASRVAASQLNTLGCPE LIARTRQEYQDIAIRLG

TDREYLKATRAKVWKARSESPLFNCKLYAMGMEMLYKKMWERYARGEKPDHVSAVDKNER EKLLTAAS

>bombyx_mori_BGIBMGA000643_OGT2.pro

MSHTCRYTQNLGNVKREQGKIDEATNLYMKALEVSLILRQLIAIWHPFYSNKAIGIQPKFADAY SNMGNTLRELQDMTG

ALACFKKAIEINPTFSDAHCNLASIYKDTGNIKEAIESYKNALYFKPDFPDAYCNLAHCLQIVCN WDDYYERMHNIIEI

VNKQLKMDKLSSVHPHHSILYPLTNEARREIATRHANLYIDKLHFLQNTVTFQHPKVMEGRLRI GYVSSDFGNHPTSHL

MQSIPGSHDRSKVEVFCYALNADDGTTFRRKIVEESENFIDLSCIPCNMEAATRIHRDGIHILINM NGYTKGARNEIFA

LKPAPIQVMWLGYPGTSGAGYIDYMITDEVSAPLSLSEDFSEKFAYMPHTYFVGDHKKMFPHL KTKYKVLIDNNSIAHE

NVALINSSEFINIDEVFNVRCDKTIVSFENLEEIEFIIRKVNIPNYVLDTTINPKQEKLYKIDPRIME SWVTILKLVPN

SVLWLLSFPAAGEPNIQKYGLNLGKDSQTECLTPGRIIFSKIACKEEHVRRGQLADICLDTPLCN GHTTTMDILWTGTP

VVTLPGDTLASRVAASQLTALHCTELIAKNRKHYEDIAIKLGTDSAYRRYIRAKVSKARLESTLF DCEHYARGLESLYS

KMWELYQRGDKPDHIAVSSK

>Dm_Q9V3X6_OGT_sxc_GT41.pro

MHVEQTRINMQSQGQSHQLPSAAHILLDQNPNSTGSNLVVKQNDIQSLSSVGLLELAHREYQAV DYESAEKHCMQLWRQ

DSTNTGVLLLLSSIHFQCRRLDKSAQFSTLAIKQNPVLAEAYSNLGNVFKERGQLQEALDNYRR AVRLKPDFIDGYINL

AAALVAARDMESAVQAYITALQYNPDLYCVRSDLGNLLKALGRLEEAKACYLKAIETCPGFAV AWSNLGCVFNAQGEIW

LAIHHFEKAVTLDPNFLDAYINLGNVLKEARIFDRAVAAYLRALNLSPNNAVVHGNLACVYYEQ GLIDLAIDTYRRAIE

LQPNFPDAYCNLANALKEKGQVKEAEDCYNTALRLCSNHADSLNNLANIKREQGYIEEATRLY LKALEVFPDFAAAHSN

LASVLQQQGKLKEALMHYKEAIRIQPTFADAYSNMGNTLKELQDVSGALQCYTRAIQINPAFA DAHSNLASIHKDSGNI

PEAIQSYRTALKLKPDFPDAYCNLAHCLQIVCDWTDYDIRMKKLVSIVTEQLEKNRLPSVHPHH SMLYPLTHDCRKAIA

ARHANLCLEKVHVLHKKPYNFLKKLPTKGRLRIGYLSSDFGNHPTSHLMQSVPGLHDRSKVEI FCYALSPDDGTTFRHK

ISRESENFVDLSQIPCNGKAADKIFNDGIHILVNMNGYTKGARNEIFALRPAPIQVMWLGYPGTS GASFMDYIITDSVT

SPLELAYQYSEKLSYMPHTYFIGDHKQMFPHLKERIIVCDKQQSSVVDNVTVINATDLSPLVENT DVKEIKEVVNAQKP

VEITHKVAELPNTTQIVSMIATGQVQTSLNGVVVQNGLATTQTNNKAATGEEVPQNIVITTRRQ YMLPDDAVVYCNFNQ

LYKIDPQTLESWVEILKNVPKSVLWLLRFPAVGEQNIKKTVSDFGISPDRVIFSNVAAKEEHVRR GQLADICLDTPLCN

GHTTSMDVLWTGTPVVTLPGETLASRVAASQLATLGCPELIARTREEYQNIAIRLGTKKEYLKA LRAKVWKARVESPLF

DCSQYAKGLEKLFLRMWEKYENGELPDHISAV

>Aedes_aegypti_XP_001662511_OGTlike.pro

MQGQVPGAQAAQPSPVAQQIQVQTQPQTVVIQQQAPQQTIQVQVQQQQVVQNSVKIDQTQQL SSVGLLELAHREYQAVD

YENAERHCMQLWRQESNNTGVLLLLSSIHFQCRRLDKSAQFSTLAIKQNPLLAEAYSNLGNVY KERGQLQEALENYRHA

VRLKPDFIDGYINLAAALVAARDMEQAVQAYVTALQYNPDLYCVRSDLGNLLKALGRLDEAKA CYLKAIETRPDFAVAW

SNLGCVFNAQGEIWLAIHHFEKAVALDPNFLDAYINLGNVLKEARIFD

>Aedes_aegypti_XP_001652461_OGTlike.pro

MSDVVLVVVVPTLFSTSSRLPVIFRNEQKNQPASESNEYTEIGRPVALGNLGSVLSAQGRHQEAK EALKAALSYRPNMA

DVHYNLGILLQNQQDYEEAVESFRKAIQFRPSLALAYLNLGTSLIALGRCQEAASVLREGSKLD GVGLRDRAAHDNARI

SALLQLGNLYADQGKLQRALAVYREALHILPDRYPPQGIYHRLGEVFARLNQWSEAERFQRAA LEAQPDHIAAHISYGS

MLARNSSRTSEAEQWFKRALRLAPADASVHHHYAEFLASVRRTAEACQYRVKAAELAPDDYSL VTAAASALRLLDRKVE

AERWYRQAVTLRPEEARSHTNLGAILHLLGRPQEAAISYKEALRLQPNDLTTLTNLAKLGIAEV S

2. Supplementary Data 2

Supplementary data 2a: Alignment using MUSCLE (default parameters) and GBlock curation in Phylogeny.fr website

Parameters used

Minimum Number Of Sequences For A Conserved Position: 5

Minimum Number Of Sequences For A Flanking Position: 7

Maximum Number Of Contiguous Nonconserved Positions: 8

Minimum Length Of A Block: 10

Allowed Gap Positions: None

Use Similarity Matrices: Yes

Flank positions of the 5 selected block(s)

Flanks: [310 325] [598 607] [609 624] [656 665] [688 700]

New number of positions in input.fasta-gb: **65** (5% of the original 1095 positions)

```
                         10        20        30        40        50        60
                 =========+=========+=========+=========+=========+=========+
Dm_eOGT          -------------------------------------------MPILPILIG--------
Hs_eOGT_Q5NDL2   --------------------------------------------MLMLFVFG--------
Ss_eOGT          --------------------------------------------MLMLLVFG--------
Ss_AGO61         -----------------------------------MHLSAVLNALLVSVLAA--------
Hs_AGO61.        -----------------------------------MHLSAVFNALLVSVLAA--------
Dm_OGT           MHVEQTRINMQSQGQSHQLPSAAHILLDQNPNSTGSNLVVKQNDIQSLSSVGLLELAHRE
Hs_OGT           ------------------------------------------------MLQG--------
Ss_OGT           -----------------------------MASSVGNVADSTEPTKRMLSFQGLAELAHRE

                         70        80        90       100       110       120
```

```
                    =========+=========+=========+=========+=========+=========+

Dm_eOGT             --------------ILHLSLAEDAKHLDGFSLPSLPSEHLIRYLNTFPKLKQQLPTNLTG
Hs_eOGT_Q5NDL2      --------------VLLHEVSLSGQNEAPPNTHSIPGEPLYNYASIR-LPEEHIPFFLHN
Ss_eOGT             --------------ALLHEVPLSGQDKAPPQADGILGAPLFNYASLR-LPEEHIPFFLHN
Ss_AGO61            --------------VLWKHVRLREHAASLEEELVLGRRAPEPAPTLRIDYPKALQILTEG
Hs_AGO61.           --------------VLWKHVRLREHAATLEEELALSRQATEPAPALRIDYPKALQILMEG
Dm_OGT              YQAVDYESAEKHCMQLWRQDSTNTGVLLLLSSIHFQCRRLDKSAQFSTLAIKQNPVLAEA
Hs_OGT              --------------HFWL---VREGIMISPSSPPPPNLFFFP-LQIFPFPFTSFPSHLLS
Ss_OGT              YQAGDFEAAERHCMQLWRQEPDNTGVLLLLSSIHFQCRRLDRSAHFSTLAIKQNPLLAEA

                           130       140       150       160       170       180
                    =========+=========+=========+=========+=========+=========+

Dm_eOGT             ------------------------------------------------------------
Hs_eOGT_Q5NDL2      ------------------------------------------------------------
Ss_eOGT             ------------------------------------------------------------
Ss_AGO61            ------------------------------------------------------------
Hs_AGO61.           ------------------------------------------------------------
Dm_OGT              YSNLGNVFKERGQLQEALDNYRRAVRLKPDFIDGYINLAAALVAARDMESAVQAYITALQ
Hs_OGT              ------------------------------------------------------------
Ss_OGT              YSNLGNVYKERGQLQEAIEHYRHALRLKPDFIDGYINLAAALVAAGDMEGAVQAYVSALQ

                           190       200       210       220       230       240
                    =========+=========+=========+=========+=========+=========+

Dm_eOGT             --------------------KGTISSAC--------------------------------
Hs_eOGT_Q5NDL2      --------------------NRHIATVCRKDSL--------------CPYKKHLEKLKYC
Ss_eOGT             --------------------NRHIATVCKKDSR--------------CPYKKYLENLKYC
```

```
Ss_AGO61        ------------GTHMVCTGRTHTDRICRFK-----------W----LCYSSEAEEFIFF

Hs_AGO61.       ------------GTHMVCTGRTHTDRICRFK-----------W----LCYSNEAEEFIFF

Dm_OGT          YNPDLYCVRSDLGNLLKALGRLEEAKACYLKAIETCPGFAVAWSNLGCVFNAQGEIWLAI

Hs_OGT          ---------------------LTPPKACYLKAIETQPNFAVAWSNLGCVFNAQGEIWLAI

Ss_OGT          YNPDLYCVRSDLGNLLKALGRLEEAKACYLKAIETQPNFAVAWSNLGCVFNAQGEIWLAI




                       250       260       270       280       290       300

                =========+=========+=========+=========+=========+=========+

Dm_eOGT         WGHERDC--------------------------------------TPAGRFQTPQCPGEH

Hs_eOGT_Q5NDL2  WGYEKSC--------------------------------------KPEFRFGYPVCSYVD

Ss_eOGT         WGYEKSC--------------------------------------KPQFRFGYPVCTYVD

Ss_AGO61        HG-------------------------------------------NASVMLPSLGSRRFQ

Hs_AGO61.       HG-------------------------------------------NTSVMLPNLGSRRFQ

Dm_OGT          HHFEKAVTLDPNFLDAYINLGNVLKEARIFDRAVAAYLRALNLSPNNAVVHGNLACVYYE

Hs_OGT          HHFEKAVTLDPNFLDAYINLGNVLKEARIFDRAVAAYLRALSLSPNHAVVHGNLACVYYE

Ss_OGT          HHFEKAVTLDPNFLDAYINLGNVLKEARIFDRAVAAYLRALSLSPNHAVVHGNLACVYYE




                       310       320       330       340       350       360

                =========+=========+=========+=========+=========+=========+

Dm_eOGT         TGWARS--KEAQVRTFYNQADFGYIQEQLSQLTPQCVPTYLGDSSLECTHY---LRFCRG

Hs_eOGT_Q5NDL2  MGWTDT--LESAEDIFWKQADFGYARERLEEMHVLCQPKETSDSSLVCSRY---LQYCRA

Ss_eOGT         MGWTDT--LESAEDIFWKQADFGYAGERLEELHVLCQAEEMNDSSLVCSRY---LQYCRA

Ss_AGO61        PALLDLSTVEDHNTQYFNFVELPAAALRFMP-----KPVFVPDVALIANRFNPDNLMHVF

Hs_AGO61.       PALLDLSTVEDHNTQYFNFVELPAAALRFMP-----KPVFVPDVALIANRFNPDNLMHVF

Dm_OGT          QGLIDLA-IDTYRRAIELQPNFPDAYCNLAN-----ALKEKGQVKEAEDCYNTALRLCSN

Hs_OGT          QGLIDLA-IDTYRRAIELQPHFPDAYCNLAN-----ALKEKGSVAEAEDCYNTALRLCPT
```

```
Ss_OGT            QGLIDLA-IDTYRRAIELQPHFPDAYCNLAN-----ALKEKGSVAEAEDCYNTALRLCPT
                           ################

                          370       380       390       400       410       420
                  =========+=========+=========+=========+=========+=========+
Dm_eOGT           R-NLLFDFRGLEQR----EERIRYHMDVLGPGQLLGHCKLNRTRLSGEMEHIGSALQSWG
Hs_eOGT_Q5NDL2    T-NLYLDLRNIKRN----HD--RFKEDFFQSGEIGGHCKLDIRTLTSEGQR-KSPLQSWF
Ss_eOGT           T-NIYLDLRNIKRN----HD--RFKEDFFQNGEIGGHCKLDIRTLMSEGQR-KSPLQSWF
Ss_AGO61          HDDLLPLFYTLRQFPGLAREARLFFMEGWGEG-----AHFDLYKLLSPKQPL--------
Hs_AGO61.         HDDLLPLFYTLRQFPGLAHEARLFFMEGWGEG-----AHFDLYKLLSPKQPL--------
Dm_OGT            HADSLNNLANIKREQGYIEEATRLYLKALEVFPDFAAAHSNLASVLQQQGKLKEALMHYK
Hs_OGT            HADSLNNLANIKREQGNIEEAVRLYRKALEVFPEFAAAHSNLASVLQQQGKLQEALMHYK
Ss_OGT            HADSLNNLANIKREQGNIEEAVRLYRKALEVFPEFAAAHSNLASVLQQQGKLQEALMHYK

                          430       440       450       460       470       480
                  =========+=========+=========+=========+=========+=========+
Dm_eOGT           PELRNFDVLPHPVLESGLCDVVVNTPTFIMKIDATYNMYHHFCDFFNLYASLFVNQSHPA
Hs_eOGT_Q5NDL2    AELQSYTQLNFRPIEDAKCDIVIEKPTYFMKLDAGVNMYHHFCDFINLYITQHVNNS---
Ss_eOGT           AELQSYTQLNFRPIEDAACDIVIEKPTYFMKLDAGVNMYHHFCDFVNLYITQHVNNS---
Ss_AGO61          -------------------------LRAQLKALGRLLCFSH----------AFVGLSKVT
Hs_AGO61.         -------------------------LRAQLKTLGRLLCFSH----------AFVGLSKIT
Dm_OGT            EAIR------IQPTFADAYSNMGNTLKELQDVSGALQCYTRAIQINPAFADAHSNLASIH
Hs_OGT            EAIR------ISPTFADAYSNMGNTLKEMQDVQGALQCYTRAIQINPAFADAHSNLASIH
Ss_OGT            EAIR------ISPTFADAYSNMGNTLKEMQDVQGALQCYTRAIQINPAFADAHSNLASIH
```

```
                       490       500       510       520       530       540
                  =========+=========+=========+=========+=========+=========+
Dm_eOGT           AFNTDVQILI-WETYPY--DSPFRDTFKAFS--QRPVWTLSDVEGKRVCFKNVVLPLLPR
Hs_eOGT_Q5NDL2    -FSTDVYIVM-WDTSSYGYGDLFSDTWNAFT--DYDVIHLKTYDSKRVCFKEAVFSLLPR
Ss_eOGT           -FSTDVYVVM-WDTSSYGYGDLFSDTWKAFT--DYDVIHLKTYDSKRVCFKEAIFSLLPR
Ss_AGO61          T----------WYQYGF-----------------------------------VQPQGPK
Hs_AGO61.         T----------WYQYGF-----------------------------------VQPQGPK
Dm_OGT            KDSGNIPEAIQSYRTALKLKPDFPDAYCNLAHCLQIVCDWTDYDIRMKKLVSIVTEQLEK
Hs_OGT            KDSGNIPEAIASYRTALKLKPDFPDAYCNLAHCLQIVCDWTDYDERMKKLVSIVADQLEK
Ss_OGT            KDSGNIPEAIASYRTALKLKPDFPDAYCNLAHCLQIVCDWTDYDERMKKLVSIVADQLEK
```

```
                       550       560       570       580       590       600
                  =========+=========+=========+=========+=========+=========+
Dm_eOGT           M-------IFGLFYNTPIIQGCSNSGLFRAFSEFILHRLQIPYKPP----------QQKI
Hs_eOGT_Q5NDL2    M-------RYGLFYNTPLISGCQNTGLFRAFAQHVLHRLNITQEGP---------KDGKI
Ss_eOGT           M-------RYGLFYNTPLISGCQNTGLFRAFSQHVLHRLNITQQGP---------KDGKI
Ss_AGO61          A--------------NILVSGNE----IRQFARFLTEKLNVSHAGG---------ALGEE
Hs_AGO61.         A--------------NILVSGNE----IRQFARFMTEKLNVSHTGV---------PLGEE
Dm_OGT            NRLPSVHPHHSMLY--PLTHDCRKA-IAARHANLCLEKVHVLHKKPYNFLKKLP-TKGRL
Hs_OGT            NRLPSVHPHHSMLY--PLSHGFRKA-IAERHGNLCLDKINVLHKPPYEHPKDLKLSDGRL
Ss_OGT            NRLPSVHPHHSMLY--PLSHGFRKA-IAERHGNLCLDKINVLHKPPYEHPKDLKLSDGRL
                                                                         ###
```

```
                       610       620       630       640       650       660
                  =========+=========+=========+=========+=========+=========+
Dm_eOGT           RITYLSRRTKYRQVLNEDELLAPLEANDKYDVQ--------------RVSYERLPFTNQL
Hs_eOGT_Q5NDL2    RVTILARSTEYRKILNQNELVNALKTVSTFEVQ------------IVDYKYRELGFLDQL
```

```
Ss_eOGT          RVTILARSTEYRKILNQNELVNALKTVSTFEVR------------IVDYKYKELAFLDQL
Ss_AGO61         YILVFSR-TQNRLILNEAELLLAL--AQEFQMK------------TVTVSLEDHAFADVV
Hs_AGO61.        YILVFSR-TQNRLILNEAELLLAL--AQEFQMK------------TVTVSLEDHTFADVV
Dm_OGT           RIGYLSSDFGNHPTSHLMQSVPGLHDRSKVEIFCYALSPDDGTTFRHKISRESENFVDLS
Hs_OGT           RVGYVSSDFGNHPTSHLMQSIPGMHNPDKFEVFCYALSPDDGTNFRVKVMAEANHFIDLS
Ss_OGT           RVGYVSSDFGNHPTSHLMQSIPGMHNPDKFEVFCYALSPDDGTNFRVKVMAEANHFIDLS
                 ####### ################                                #####

                        670       680       690       700       710       720
                 =========+=========+=========+=========+=========+=========+
Dm_eOGT          AITRN-----------TDILIGMHG--AGLTHLLFLPNWACIFEL--------------Y
Hs_eOGT_Q5NDL2   RITHN-----------TDIFIGMHG--AGLTHLLFLPDWAAVFEL--------------Y
Ss_eOGT          RITHN-----------TDIFIGMHG--AGLTHLLFLPDWAAVFEL--------------Y
Ss_AGO61         RLVSN-----------ASMLVSMHG--AQLVTALFLPRGAAVVELFP------------Y
Hs_AGO61.        RLVSN-----------ASMLVSMHG--AQLVTTLFLPRGATVVELFP------------Y
Dm_OGT           QIPCNGKAADKIFNDGIHILVNMNGYTKGARNEIFALRPAPIQVMWLGYPGTSGASFMDY
Hs_OGT           QIPCNGKAADRIHQDGIHILVNMNGYTKGARNELFALRPAPIQAMWLGYPGTSGALFMDY
Ss_OGT           QIPCNGKAADRIHQDGIHILVNMNGYTKGARNELFALRPAPIQAMWLGYPGTSGALFMDY
                 #####                      #############

                        730       740       750       760       770       780
                 =========+=========+=========+=========+=========+=========+
Dm_eOGT          NCEDPNC---------------------------------------------YKDLARL
Hs_eOGT_Q5NDL2   NCEDERC---------------------------------------------YLDLARL
Ss_eOGT          NCGDERC---------------------------------------------YLDLARL
Ss_AGO61         AVNPDHYTP-------------------------------------------YKTLATL
Hs_AGO61.        AVNPDHYTP-------------------------------------------YKTLAML
Dm_OGT           IITDSVTSPLELAYQYSEKLSYMPHTYFIGDHKQMFPHLKERIIVCDKQQSSVVDNVTVI
```

```
Hs_OGT            IITDQETSPAEVAEQYSEKLAYMPHTFFIGDHANMFPHLKKKAVIDFKSNGHIYDNRIVL

Ss_OGT            IITDQETSPAEVAEQYSEKLAYMPHTFFIGDHANMFPHLKKKAVIDFKSNGHIYDNRIVL


                         790       800       810       820       830       840

                  =========+=========+=========+=========+=========+=========+

Dm_eOGT           RGVR--YRTWEQR-----------------------------------------------

Hs_eOGT_Q5NDL2    RGVH--YITWRRQ-----------------------------------------------

Ss_eOGT           RGVH--YITWRRQ-----------------------------------------------

Ss_AGO61          PGMDLQYIAWRNTMPENTVTHPERPWDQGGIAHLDRAEQ---------------------

Hs_AGO61.         PGMDLQYVAWRNMMPENTVTHPERPWDQGGITHLDRAEQ---------------------

Dm_OGT            NATD--LSPLVEN-TDVKEIK-EVVNAQKPVEITHKVAELPN------TTQIVSMIATGQ

Hs_OGT            NGID--LKAFLDSLPDVKIVKMKCPDGGDNADSSNTALNMPVIPMNTIAEAVIEMINRGQ

Ss_OGT            NGID--LKAFLDSLPDVKIVKMKCPDGGDNADSSNTALNMPVIPMNTIAEAVIEMINRGQ


                         850       860       870       880       890       900

                  =========+=========+=========+=========+=========+=========+

Dm_eOGT           -----------------------------------------------------DLVYPQD

Hs_eOGT_Q5NDL2    -----------------------------------------------------NKVFPQD

Ss_eOGT           -----------------------------------------------------NKVFPQD

Ss_AGO61          --------------------ARILQSQEVPRHLCCRNPEWL------------FRIYQDT

Hs_AGO61.         --------------------ARILQSREVPRHLCCRNPEWL------------FRIYQDT

Dm_OGT            VQTSLNGVVVQNGLATTQTNNKAATGEEVPQNIVITTRRQYMLPDDAVVYCNFNQLYKID

Hs_OGT            IQITINGFSISNGLATTQINNKAATGEEVPRTIIVTTRSQYGLPEDAIVYCNFNQLYKID

Ss_OGT            IQITINGFSISNGLATTQINNKAATGEEVPRTIIVTTRSQYGLPEDAIVYCNFNQLYKID
```

```
                    910       920       930       940       950       960
               =========+=========+=========+=========+=========+=========+
Dm_eOGT        EGHHPE---------------------GGAHAKFTNYSFDVK------------------
Hs_eOGT_Q5NDL2 KGHHPT---------------------LGEHPKFTNYSFDVE------------------
Ss_eOGT        KGHHPT---------------------LGEHPKFTNYSFDVE------------------
Ss_AGO61       KVDIPSLIQTIRRVVK-----------GRPGPRKQKWTVSLYPGKVREARCQSSVQGASE
Hs_AGO61.      KVDIPSLIQTIRRVVK-----------GRPGPRKQKWTVGLYPGKVREARCQASVHGASE
Dm_OGT         PQTLESWVEILKNVPKSVLWLLRFPAVGEQNIKKTVSDFGISPDRVIFSNVAAKEEHVRR
Hs_OGT         PSTLQMWANILKRVPNSVLWLLRFPAVGEPNIQQYAQNMGLPQNRIIFSPVAPKEEHVRR
Ss_OGT         PSTLQMWANILKRVPNSVLWLLRFPAVGEPNIQQYAQNMGLPQNRIIFSPVAPKEEHVRR
```

```
                    970       980       990      1000      1010      1020
               =========+=========+=========+=========+=========+=========+
Dm_eOGT        ------------------------------------------------------------
Hs_eOGT_Q5NDL2 ------------------------------------------------------------
Ss_eOGT        ------------------------------------------------------------
Ss_AGO61       ARL-------------TVSWQIPW--------------------NLKYLKVREV------
Hs_AGO61.      ARL-------------TVSWQIPW--------------------NLKYLKVREV------
Dm_OGT         GQLADICLDTPLCNGHTTSMDVLWTGTPVVTLPGETLASRVAASQLATLGCPELIARTRE
Hs_OGT         GQLADVCLDTPLCNGHTTGMDVLWAGTPMVTMPGETLASRVAASQLTCLGCLELIAKNRQ
Ss_OGT         GQLADVCLDTPLCNGHTTGMDVLWAGTPMVTMPGETLASRVAASQLTCLGCLELIAKNRQ
```

```
                   1030      1040      1050      1060      1070      1080
               =========+=========+=========+=========+=========+=========+
Dm_eOGT        EFVHLVDGAAEE-------ILSHKEFPRRASE-----NPSKTQRNEL-------------
```

Hs_eOGT_Q5NDL2	EFMYLVLQAADH-------VLQHPKW------------PFKKKHDEL-------------
Ss_eOGT	EFMFLVLQAADH-------VLQHPKW------------PFKNKHDEL-------------
Ss_AGO61	KYEVWLQEQGENTYVPYILTLQNHTFTENI-------KPFTTYL--VWVRCIFNKTLLGP
Hs_AGO61.	KYEVWLQEQGENTYVPYILALQNHTFTENI-------KPFTTYL--VWVRCIFNKILLGP
Dm_OGT	EYQNIAIRLGTK--KEYLKALRAKVWKARVESPLFDCSQYAKGLEKLFLR-MWEKYENGE
Hs_OGT	EYEDIAVKLGTD--LEYLKKVRGKVWKQRISSPLFNTKQYTMELERLYLQ-MWEHYAAGN
Ss_OGT	EYEDIAVKLGTD--LEYLKKIRGKVWKQRISSPLFNTKQYTMELERLYLQ-MWEHYAAGN

	1090
	=========+=====
Dm_eOGT	---------------
Hs_eOGT_Q5NDL2	---------------
Ss_eOGT	---------------
Ss_AGO61	FADVLVCNT------
Hs_AGO61.	FADVLVCNT------
Dm_OGT	LPDHISAV-------
Hs_OGT	KPDHMIKPVEVTESA
Ss_OGT	KPDHMIKPVEVTESA

Supplementary data 2b: Alignment using MUSCLE (default parameters) and no curation in Phylogeny.fr website

```
AeO2            ------------------------------------------------------------
Ae.a.OGTli      ------------------------------------------------------------
b.mo.GT41       ------------------------------------------------------------
H.G.OGT2        ------------------------------------------------------------
X.l.GT41_O      ------------------------------------------------------------
x.t.GT41_O      ------------------------------------------------------------
G.G.OGT         ------------------------------------------------------------
M.G.OGT         ------------------------------------------------------------
B.G.OGT         ------------------------------------------------------------
H.G.OGT1        ------------------------------------------------------------
S.G.OGT         ------------------------------------------------------------
eG1             ------------------------------------------------------------
e.c.GT41_O      ------------------------------------------------------------
Da.re.GT41      ------------------------------------------------------------
te.n.GT41       ------------------------------------------------------------
Dm.G.OGT        ------------------------------------------------------------
a.g.GT41_O      mssvenaengvgkysyqtvyyiitnnnnnsteegesdqvenlhaitaavdseltrkrrri
Ae.a.GT41       ------------------------------------------------------------
bG1             ------------------------------------------------------------
Ap.me.GT41      ------------------------------------------------------------
ApG2            --------------------------------------------------------msir
Da.re.AGO6      ------------------------------------------------------------
te.n.AGO61      ------------------------------------------------------------
ta.ru.AGO6      ------------------------------------------------------------
M.AGO61         ------------------------------------------------------------
R.AGO61         ------------------------------------------------------------
H.AGO61         ------------------------------------------------------------
e.c.AGO61       ------------------------------------------------------------
```

```
S.AGO61         ------------------------------------------------------------

B.AGO61         ------------------------------------------------------------

G.AGO611        ------------------------------------------------------------

x.t.AGO61       ------------------------------------------------------------

X.l.AGO61       ------------------------------------------------------------

ta.ru.eOGT      ------------------------------------------------------------

te.n.eOGT       ------------------------------------------------------------

X.l.eOGT        ------------------------------------------------------------

x.t.eOGT        ------------------------------------------------------------

G.eOGT          ------------------------------------------------------------

M.eOGT          ------------------------------------------------------------

R.eOGT          ------------------------------------------------------------

S.eOGT          ------------------------------------------------------------

B.eOGT          ------------------------------------------------------------

H.eOGT          ------------------------------------------------------------

e.c.eOGT        ------------------------------------------------------------

Dm.eOGT         ------------------------------------------------------------

a.g.eOGT        ------------------------------------------------------------

Ae.a.eOGT       ------------------------------------------------------------

Ap.me.eOGT      ------------------------------------------------------------

b.mo.eOGT       ------------------------------------------------------------


AeO2            ------------------------------------------------------------

Ae.a.OGTli      --mqgQvpgaqaAqpspvaqQiqvQtqpQtvviqqqapqqtiQvqvqqqqvvqnSvkidq

b.mo.GT41       ------------------------------------------------------------

H.G.OGT2        ------------------------------------------------------------

X.l.GT41_O      ------------------------------MAASVGNVADSTEPTkRMLSFQG-------

x.t.GT41_O      ------------------------------MAASVGsVADST----------G-------

G.G.OGT         ------------------------------MATSVGNVADSTEPTkRMLSFQG-------

M.G.OGT         ------------------------------MASSVGNVADST----------G-------

B.G.OGT         ------------------------------MASSVGNVADST----------G-------

H.G.OGT1        ------------------------------MASSVGNVADSTEPTkRMLSFQG-------
```

```
S.G.OGT       ------------------------------MASSVGNVADSTEPTkRMLSFQG-------
eG1           ------------------------------MASSVGNVADSTEPTkRMLSFQG-------
e.c.GT41_O    ------------------------------MASSVGNVADST----------G-------
Da.re.GT41    ------------------------------MASSVGNVADSTEPTkRVLSFQG-------
te.n.GT41     ------------------------------MATSVGNVADST----------G-------
Dm.G.OGT      -mhveQtrinmqSqgqshqlpsaahilldQnpNStGsnlvvkQndiqsLSsvG-------
a.g.GT41_O    eAikmQgqipgaAAvqvqggspqaaalaaQqqqqqqqsisvkmdpanqLSsaG-------
Ae.a.GT41     ------------------------------------------------------------
bG1           ---------mqpqAnvavpqsvttQpqaQQivgvpaNavilkmsdlqqIStvG-------
Ap.me.GT41    -----------mvAiimqaqQqpqQqqqQnhqqiaGtsvilkmneiqqLStvG-------
ApG2          lAekkenviknsSgiignvvnssddvcngtvewqnGqnllvvtqdtqgkeliaiAdnqti
Da.re.AGO6    ------------------------------------------------------------
te.n.AGO61    ------------------------------------------------------------
ta.ru.AGO6    ------------------------------------------------------------
M.AGO61       ------------------------------------------------------------
R.AGO61       ------------------------------------------------------------
H.AGO61       ------------------------------------------------------------
e.c.AGO61     ------------------------------------------------------------
S.AGO61       ------------------------------------------------------------
B.AGO61       ------------------------------------------------------------
G.AGO611      ------------------------------------------------------------
x.t.AGO61     ------------------------------------------------------------
X.l.AGO61     ------------------------------------------------------------
ta.ru.eOGT    ------------------------------------------------------------
te.n.eOGT     ------------------------------------------------------------
X.l.eOGT      ------------------------------------------------------------
x.t.eOGT      ------------------------------------------------------------
G.eOGT        ------------------------------------------------------------
M.eOGT        ------------------------------------------------------------
R.eOGT        ------------------------------------------------------------
S.eOGT        ------------------------------------------------------------
B.eOGT        ------------------------------------------------------------
```

```
H.eOGT          ------------------------------------------------------------

e.c.eOGT        ------------------------------------------------------------

Dm.eOGT         ------------------------------------------------------------

a.g.eOGT        ------------------------------------------------------------

Ae.a.eOGT       ------------------------------------------------------------

Ap.me.eOGT      ------------------------------------------------------------

b.mo.eOGT       ------------------------------------------------------------


AeO2            ------------------------------------------------------------

Ae.a.OGTli      tqqLsSvgLlELAHREYQAvDyEnAERHCMQLWRQEsnNTGVLLLLSSIHFQCRRLDkSA

b.mo.GT41       ------------------------------------------------------------

H.G.OGT2        -----------------------------------------------------------m

X.l.GT41_O      --------LAELAHREYQAGDFEAAERHCMQLWRQEPDNTGVLLLLSSIHFQCRRLDRSA

x.t.GT41_O      --------LAELAHREYQAGDFEAAERHCMQLWRQEPDNTGVLLLLSSIHFQCRRLDRSA

G.G.OGT         --------LAELAHREYQAGDFEAAERHCMQLWRQEPDNTGVLLLLSSIHFQCRRLDRSA

M.G.OGT         --------LAELAHREYQAGDFEAAERHCMQLWRQEPDNTGVLLLLSSIHFQCRRLDRSA

B.G.OGT         --------LAELAHREYQAGDFEAAERHCMQLWRQEPDNTGVLLLLSSIHFQCRRLDRSA

H.G.OGT1        --------LAELAHREYQAGDFEAAERHCMQLWRQEPDNTGVLLLLSSIHFQCRRLDRSA

S.G.OGT         --------LAELAHREYQAGDFEAAERHCMQLWRQEPDNTGVLLLLSSIHFQCRRLDRSA

eG1             --------LAELAHREYQAGDFEAAERHCMQLWRQEPDNTGVLLLLSSIHFQCRRLDRSA

e.c.GT41_O      --------LAELAHREYQAGDFEAAERHCMQLWRQEPDNTGVLLLLSSIHFQCRRLDRSA

Da.re.GT41      --------LAELAHREYQsGDFEAAERHCMQLWRQEPDNTGVLLLLSSIHFQCRRLDRSA

te.n.GT41       --------LAELAHREYQsGDFEAAERHCMQLWRQEPDNTamL------------atRdd

Dm.G.OGT        --------LlELAHREYQAvDyEsAEkHCMQLWRQdstNTGVLLLLSSIHFQCRRLDkSA

a.g.GT41_O      --------LlELAHREYQAvDydnAERHCMQLWRQEsnNTGVLLLLSSIHFQCRRLDkSA

Ae.a.GT41       ------------------------------------------------------------

bG1             --------LlELAHREYQAGDyEsAEhHCMQLWRQdstNTGVLLLLSSIHFQCRRLDkSA

Ap.me.GT41      --------LlELAHREYQAGDyEnAERHCMQLWRQEtnNTGVLLLLSSIHFQCRRLekSA

ApG2            nvaVdSywLlELAHREYQAGDyEnAERHCMQLWRQEtnNTGVLLLLSSIHFQCRRLekSA

Da.re.AGO6      ------------------------------------------------------------

te.n.AGO61      ------------------------------------------------------------

ta.ru.AGO6      ------------------------------------------------------------
```

```
M.AGO61        ------------------------------------------------------------
R.AGO61        ------------------------------------------------------------
H.AGO61        ------------------------------------------------------------
e.c.AGO61      ------------------------------------------------------------
S.AGO61        ------------------------------------------------------------
B.AGO61        ------------------------------------------------------------
G.AGO611       ------------------------------------------------------------
x.t.AGO61      ------------------------------------------------------------
X.l.AGO61      ------------------------------------------------------------
ta.ru.eOGT     ------------------------------------------------------------
te.n.eOGT      ------------------------------------------------------------
X.l.eOGT       ------------------------------------------------------------
x.t.eOGT       ------------------------------------------------------------
G.eOGT         ------------------------------------------------------------
M.eOGT         ------------------------------------------------------------
R.eOGT         ------------------------------------------------------------
S.eOGT         ------------------------------------------------------------
B.eOGT         ------------------------------------------------------------
H.eOGT         ------------------------------------------------------------
e.c.eOGT       ------------------------------------------------------------
Dm.eOGT        ------------------------------------------------------------
a.g.eOGT       ------------------------------------------------------------
Ae.a.eOGT      ------------------------------------------------------------
Ap.me.eOGT     ----------------------------------------------------------mm
b.mo.eOGT      ------------------------------------------------------------

AeO2           msdvvlVVvvPtLfstsSrLpv-------------ifRneQknqpasesneYteIgRpv-
Ae.a.OGTli     qFstLAIkqNPLLaEAYSNLGNVYKERGQlQEALEnYRHA-vRLKPDFIDgYiNLAaALV
b.mo.GT41      ------------------------------------msHt--------------------
H.G.OGT2       lqghfwLVregimispsSp-------------------------pPpnlffF--------
X.l.GT41_O     HFstLAIkqNPLLaEAYSNLGNVYKERGQlQEAIEhYRHA-LRLKPDFIDgYiNLAaALV
x.t.GT41_O     HFstLAIkqNPLLaEAYSNLGNVYKERGQlQEAIEhYRHA-LRLKPDFIDgYiNLAaALV
```

```
G.G.OGT        HFstLAIkqNPLLaEAYSNLGNVYKERGQlQEAIEhYRHA-LRLKPDFIDgYiNLAaALV
M.G.OGT        HFstLAIkqNPLLaEAYSNLGNVYKERGQlQEAIEhYRHA-LRLKPDFIDgYiNLAaALV
B.G.OGT        HFstLAIkqNPLLaEAYSNLGNVYKERGQlQEAIEhYRHA-LRLKPDFIDgYiNLAaALV
H.G.OGT1       HFstLAIkqNPLLaEAYSNLGNVYKERGQlQEAIEhYRHA-LRLKPDFIDgYiNLAaALV
S.G.OGT        HFstLAIkqNPLLaEAYSNLGNVYKERGQlQEAIEhYRHA-LRLKPDFIDgYiNLAaALV
eG1            HFstLAIkqNPLLaEAYSNLGNVYKERGQlQEAIEhYRHA-LRLKPDFIDgYiNLAaALV
e.c.GT41_O     HFstLAIkqNPLLaEAYSNLGNVYKERGQlQEAIEhYRHA-LRLKPDFIDgYiNLAaALV
Da.re.GT41     HFstLAIkqNPmLaEAYSNLGNVYKERGQlQEAIEhYRHA-LRLKPDFIDgYiNLAaALV
te.n.GT41      fFytLAIkqNPLLaEAYSNLGNVYKERGQlQEAIEhYRHA-LRLKPDFIDgYiNLAaALV
Dm.G.OGT       qFstLAIkqNPvLaEAYSNLGNVFKERGQlQEALDnYRrA-vRLKPDFIDgYiNLAaALV
a.g.GT41_O     qFstLAIkqNPLLaEAYSNLGNVYKERGQlQEALEnYRHA-vRLKPDFIDgYiNLAaALV
Ae.a.GT41      ------------------------------------------------------------
bG1            HFstLAIkqNPLLaEAYSNLGNVYKERGQlQEALEnYRHA-vRLKPDFIDgYiNLAaALV
Ap.me.GT41     HyssLAIkqNPLLaEAYSNLGNVFKERGQlQEALEnYRHA-vRLKPDFIDgYiNLAaALV
ApG2           HyssLAIkqNPLLaEAYSNLGNVFKERGQlQEALEnYRHA-vRLKPDFIDgYiNLAaALV
Da.re.AGO6     --MnLpaVlNgLLvsvvAaL---------------lwKyvRLvehtsqlEeeLqLtR---
te.n.AGO61     --MgvgtLlNgLLvsvvAaL---------------lwKySKLsehaallEeeLhMtR---
ta.ru.AGO6     --MsvgtLlNgLLvsivAaL---------------lwKySKLsehaallEeeLhMtR---
M.AGO61        --MhLSaVfNaLLvsvlAaV---------------lwKHvRLRehaatlEeeLaLgQq--
R.AGO61        --MhLSaVfNaLLvsvlAaV---------------lwKHvRLRehaatlEeeLaLgQq--
H.AGO61        --MhLSaVfNaLLvsvlAaV---------------lwKHvRLRehaatlEeeLaLSRq--
e.c.AGO61      --MhLSaVfNaLLvsvlAaV---------------lwKHvRLRehaatlEeeLaLSRq--
S.AGO61        --MhLSaVlNaLLvsvlAaV---------------lwKHvRLRehaaslEeeLvLgRr--
B.AGO61        --MhLSaVlNaLLvsvlAaV---------------lwKHvRLRehaaslEeeLaVgRr--
G.AGO611       --MniAaVfNaLLvsvlAtV---------------lwKyiKLRehafmVEeeLvLmR---
x.t.AGO61      --MniSaVfNaLLvsimAaV---------------lwKHvKLleqfyvIEeeLeLtR---
X.l.AGO61      --MniSaVfsaLLvsimAaV---------------lwKHvKLldqfyvIEeeLeLtR---
ta.ru.eOGT     --MlLeVVlgivfpfAvvta-----DsatnhkpapllsyngtsLplDhVpyFLNnnKkL-
te.n.eOGT      --MlLeVVlvsvfsfcvvta-----EspvnntpVpltsyngisLplqhVpyFfNnnKkL-
X.l.eOGT       ---mvpLrlvlLLhiiHfSceNevgsaanngsAq-lYnyrKihLpdDhIpyYLhsnRhv-
x.t.eOGT       ---mvpLwlllLfhviHfShGNeidsaasngsAL-sYhygKLyLpdDhIpyYLhsnRhi-
G.eOGT         --MfilLMfvlLLqEilANsrdenltelnsvleeptYsyrainLpaEhIpyFLhnnRhi-
```

```
M.eOGT        --MlmlLVfgvLLhEvplSgqdkahseaddapgkalYdySsLRLpaEhIpfFLhnnRhv-
R.eOGT        --MlmlLVfgvLLhEvplSgqdeahpeadrvpgealYdySsLRLpeEhIpfFLhsnRhv-
S.eOGT        --MlmlLVfgaLLhEvplSgqdkappqadgilgaplfnyAsLRLpeEhIpfFLhnnRhi-
B.eOGT        --MfmlLVfgaLLpEvplSgqdkappqadgisAtplfnyAsLRLpeEhIpfFLhnnRhi-
H.eOGT        --MlmlfVfgvLLhEvslSgqNeappnthsipgeplYnyAsiRLpeEhIpfFLhnnRhi-
e.c.eOGT      --MlmlLVfgvLLhDiplSgqaeaspeadgipgeplfnyAsiRLpeEhIpfFLhnnghi-
Dm.eOGT       --MpilpIligiLhlslAe------------DAkhldgfSlpsLpsEhlirYLNtfpkL-
a.g.eOGT      -----------mvqyipSatcgk------------pYey--inLpkahlpmYfkrfpAL-
Ae.a.eOGT     ----mirVllvvLtfgafagGsknd-----------Yey--inLpkshlplYfrrfprL-
Ap.me.eOGT    idhaLSIkcsvtivivFAvtst-------------ysnytdidLpsDhIkyYfNyfptv-
b.mo.eOGT     ------------------------------------------------------------

AeO2          ----------------------------------ALgnlGsvl-------------saqG
Ae.a.OGTli    AArDMEqAVQAYVtAlQYnPDLYCVrSDLGNlLKALgRldEAK-------------ACYl
b.mo.GT41     ---------------------------------------------------------C--
H.G.OGT2      --------------plQifPfpFtsFp---ShLlSLtppk----------------ACYl
X.l.GT41_O    AAGDMEGAVQAYVSAlQYnPDLYCVrSDLGNlLKALgRleEAK-------------ACYl
x.t.GT41_O    AAGDMEGAVQAYVSAlQYnPDLYCVrSDLGNlLKALgRleEAK-------------ACYl
G.G.OGT       AAGDMEGAVQAYVSAlQYnPDLYCVrSDLGNlLKALgRleEAK-------------ACYl
M.G.OGT       AAGDMEGAVQAYVSAlQYnPDLYCVrSDLGNlLKALgRleEAK-------------ACYl
B.G.OGT       AAGDMEGAVQAYVSAlQYnPDLYCVrSDLGNlLKALgRleEAK-------------ACYl
H.G.OGT1      AAGDMEGAVQAYVSAlQYnPDLYCVrSDLGNlLKALgRleEAK-------------ACYl
S.G.OGT       AAGDMEGAVQAYVSAlQYnPDLYCVrSDLGNlLKALgRleEAK-------------ACYl
eG1           AAGDMEGAVQAYVSAlQYnPDLYCVrSDLGNlLKALgRleEAK-------------ACYl
e.c.GT41_O    AAGDMEGAVQAYVSAlQYnPDLYCVrSDLGNlLKALgRleEAK-------------ACYl
Da.re.GT41    AAGDMEGAVQAYVSAlQYnPDLYCVrSDLGNlLKALgRleEAK-------------ACYl
te.n.GT41     AAGDMEGAVQAYVSAlQYnPDLYCVrSDLGNlLKALgRleEAKpaaatsltksknmACYl
Dm.G.OGT      AArDMEsAVQAYitAlQYnPDLYCVrSDLGNlLKALgRleEAK-------------ACYl
a.g.GT41_O    AArDMEqAVQAYVtAlQYnPDLYCVrSDLGNlLKALgRldEAK-------------ACYl
Ae.a.GT41     ------------------------------------------------------------
bG1           AAGDMEqAVQAYVtAlQYnPDLYCVrSDLGNlLKALgRldEAK-------------ACYl
Ap.me.GT41    AAGDMEqAVQAYVtAlQYnPDLYCVrSDLGNlLKALaRldEAK-------------ACYl
```

```
ApG2          AAGDMEqAVQAYVtAlQYnPDLYCVrSDLGNlLKALaRldEAK-------------ACYl
Da.re.AGO6    --------------qsQefsqVridYh---gaLlALqehGtrm-------------vCtG
te.n.AGO61    --------------rsQelsqaHidYh---vaLqALqehGtrm-------------vCtG
ta.ru.AGO6    --------------qsQelsqaHidYh---vaLqALqehGtrm-------------vCtG
M.AGO61       --------------sldpvlgLkidYp---kaLqiLmegGthm-------------vCtG
R.AGO61       --------------sldpvPgLridYp---kaLqiLmegGthm-------------vCtG
H.AGO61       --------------AtEpaPaLridYp---kaLqiLmegGthm-------------vCtG
e.c.AGO61     --------------ApEpaPaLridYp---kaLqiLmegGthm-------------vCtG
S.AGO61       --------------ApEpaPtLridYp---kaLqiLtegGthm-------------vCtG
B.AGO61       --------------AadpaPaLridYp---kaLqiLtegGthm-------------vCtG
G.AGO611      --------------qsQelsqVqidYh---aaLqtLledGtrm-------------vCtG
x.t.AGO61     --------------qsQelsqVridYq---aaLqALvedGtrm-------------vCsG
X.l.AGO61     --------------qsQelsqVridYq---aaLqALvedGtrm-------------vCsG
ta.ru.eOGT    --------------AkQcrsDplCpFr---vscdALqdls----------------vCwG
te.n.eOGT     --------------AkQcrsDpFCpFkvrhDaLqdLs-------------------vCwG
X.l.eOGT      --------------AalclqDLHCpYk---qhLqnLns-------------------CwG
x.t.eOGT      --------------AalcrrDpHCpFk---qhLqnLn-------------------sCwG
G.eOGT        --------------AgickqDsrCpYk-vGfyfvlhkylkklK-------------sCwG
M.eOGT        --------------AsvcreDsHCpYk---khLenLny-------------------CwG
R.eOGT        --------------AsvcreDsHCpYk---khLeSLns-------------------CwG
S.eOGT        --------------AtvckkDsrCpYk---kyLenLky-------------------CwG
B.eOGT        --------------AtvcrkDsHCpYk---kyLenLky-------------------CwG
H.eOGT        --------------AtvcrkDslCpYk---khLekLky-------------------CwG
e.c.eOGT      --------------AtvckkDsHCpYk---khLenLky-------------------CwG
Dm.eOGT       -------------------------kqqLptnLtgkgtiss---------------ACwG
a.g.eOGT      --------------ekQcaeDetCpYr---tviaSqsyqnrke-------------gCwG
Ae.a.eOGT     --------------ekrclkDetCeYq---klLKSeafkakkd-------------tCwG
Ap.me.eOGT    --------------AqEcrnntvCpYk---DsLdtk--------------------ACwG
b.mo.eOGT     ------------------------------------------------------------

AeO2          RhqEaKealkaA------------------------LsyrPNmaDvHyNLGilLqnqqdy
Ae.a.OGTli    KAiETRPdFAVAWSNLGCVFNAQGEIWLAIHHFEKAVaLDPNFLDAYINLGNVLKEARIF
```

```
b.mo.GT41       ------------------------------------------------------------
H.G.OGT2        KAiETQPNFAVAWSNLGCVFNAQGEIWLAIHHFEKAVtLDPNFLDAYINLGNVLKEARIF
X.l.GT41_O      KAiETQPNFAVAWSNLGCVFNAQGEIWLAIHHFEKAVtLDPNFLDAYINLGNVLKEARIF
x.t.GT41_O      KAiETQPNFAVAWSNLGCVFNAQGEIWLAIHHFEKAVtLDPNFLDAYINLGNVLKEARIF
G.G.OGT         KAiETQPNFAVAWSNLGCVFNAQGEIWLAIHHFEKAVtLDPNFLDAYINLGNVLKEARIF
M.G.OGT         KAiETQPNFAVAWSNLGCVFNAQGEIWLAIHHFEKAVtLDPNFLDAYINLGNVLKEARIF
B.G.OGT         KAiETQPNFAVAWSNLGCVFNAQGEIWLAIHHFEKAVtLDPNFLDAYINLGNVLKEARIF
H.G.OGT1        KAiETQPNFAVAWSNLGCVFNAQGEIWLAIHHFEKAVtLDPNFLDAYINLGNVLKEARIF
S.G.OGT         KAiETQPNFAVAWSNLGCVFNAQGEIWLAIHHFEKAVtLDPNFLDAYINLGNVLKEARIF
eG1             KAiETQPNFAVAWSNLGCVFNAQGEIWLAIHHFEKAVtLDPNFLDAYINLGNVLKEARIF
e.c.GT41_O      KAiETQPNFAVAWSNLGCVFNAQGEIWLAIHHFEKAVtLDPNFLDAYINLGNVLKEARIF
Da.re.GT41      KAiETQPNFAVAWSNLGCVFNAQGEIWLAIHHFEKAVtLDPNFLDAYINLGNVLKEARIF
te.n.GT41       KAiETQPNFAVAWSNLGCVFNAQGEIWLAIHHFEKAVtLDPNFLDAYINLGNVLKEARIF
Dm.G.OGT        KAiETcPgFAVAWSNLGCVFNAQGEIWLAIHHFEKAVtLDPNFLDAYINLGNVLKEARIF
a.g.GT41_O      KAiETRPdFAVAWSNLGCVFNAQGEIWLAIHHFEKAVaLDPNFLDAYINLGNVLKEARIF
Ae.a.GT41       -------------------------------mF---------------------------
bG1             KAiETRPdFAVAWSNLGCVFNAQsEIWLAIHHFEKAVaLDPNFLDAYINLGNVLKEARIF
Ap.me.GT41      KAiETRPdFAVAWSNLGCVFNAQGEIWLAIHHFEKAVaLDPNFLDAYINLGNVLKEARIF
ApG2            KAiETRPdFAVAWSNLGCVFNAQGEIWLAIHHFEKAVaLDPNFLDAYINLGNVLKEARIF
Da.re.AGO6      Kmht--------------------------------------------------------
te.n.AGO61      Kmht--------------------------------------------------------
ta.ru.AGO6      Kmht--------------------------------------------------------
M.AGO61         Rtht--------------------------------------------------------
R.AGO61         Rtht--------------------------------------------------------
H.AGO61         Rtht--------------------------------------------------------
e.c.AGO61       Rtht--------------------------------------------------------
S.AGO61         Rtht--------------------------------------------------------
B.AGO61         Rtht--------------------------------------------------------
G.AGO611        Rmht--------------------------------------------------------
x.t.AGO61       Rmht--------------------------------------------------------
X.l.AGO61       Rmht--------------------------------------------------------
ta.ru.eOGT      yekncdPgkrfs--------------------YpvctraDygwthsletareIfwkqadF
```

te.n.eOGT	yekncdPgkrfs--------------------YpvcIraDsgwthsletareIfwkqadF
X.l.eOGT	yektcaeghrfg--------------------YpvcdqVDfgwaktieesqqVfwrqadF
x.t.eOGT	yeksctkghgys--------------------YpvcdqVDfgwaktieesqeVfwkqadF
G.eOGT	yekscKsdYrfs--------------------YpvcdyVesgwandietaqqIfwkqadF
M.eOGT	yekscaPeFrfg--------------------spvcsyVDlgwtDtlesaqdmfwrqadF
R.eOGT	yeksctPesrfg--------------------spicsyVDlgwtDtlesaqdmfwkqadF
S.eOGT	yekscKPqFrfg--------------------YpvctyVDmgwtDtlesaedIfwkqadF
B.eOGT	yekscRPeFrfg--------------------YpvctyVDmgwtDtlesaqeIfwkqadF
H.eOGT	yekscKPeFrfg--------------------YpvcsyVDmgwtDtlesaedIfwkqadF
e.c.eOGT	yekscKPeFrfg--------------------YpvctyVDmgwtDtlesaqdIfwkqadF
Dm.eOGT	herDctPagrfq--------------------tpqcpgehtgwarskeaqvrtfynqadF
a.g.eOGT	yeegcternryA--------------------nhscpgshigyvkskqaqldtfysqadF
Ae.a.eOGT	yedDcRkenrfs--------------------kpecpgdfhgyvkskeaqletfyaqadF
Ap.me.eOGT	yepncKaensfs--------------------vpqcpgdhrgwvttkkaqvetfyaqgdF
b.mo.eOGT	--

AeO2	eeAVesFrkAiqfrPSlALa----------------------------------YlNlgt
Ae.a.OGTli	D---
b.mo.GT41	--
H.G.OGT2	DRAVAaYlRAlsLsPNHAVvHGNLACVYYEQGLIDLAIDTYRRAIELQPHFPDAYCNlAN
X.l.GT41_O	DRAVAaYlRAlsLsPNHAVvHGNLACVYYEQGLIDLAIDTYRRAIELQPHFPDAYCNlAN
x.t.GT41_O	DRAVAaYlRAlsLsPNHAVvHGNLACVYYEQGLIDLAIDTYRRAIELQPHFPDAYCNlAN
G.G.OGT	DRAVAaYlRAlsLsPNHAVvHGNLACVYYEQGLIDLAIDTYRRAIELQPHFPDAYCNlAN
M.G.OGT	DRAVAaYlRAlsLsPNHAVvHGNLACVYYEQGLIDLAIDTYRRAIELQPHFPDAYCNlAN
B.G.OGT	DRAVAaYlRAlsLsPNHAVvHGNLACVYYEQGLIDLAIDTYRRAIELQPHFPDAYCNlAN
H.G.OGT1	DRAVAaYlRAlsLsPNHAVvHGNLACVYYEQGLIDLAIDTYRRAIELQPHFPDAYCNlAN
S.G.OGT	DRAVAaYlRAlsLsPNHAVvHGNLACVYYEQGLIDLAIDTYRRAIELQPHFPDAYCNlAN
eG1	DRAVAaYlRAlsLsPNHAVvHGNLACVYYEQGLIDLAIDTYRRAIELQPHFPDAYCNlAN
e.c.GT41_O	DRAVAaYlRAlsLsPNHAVvHGNLACVYYEQGLIDLAIDTYRRAIELQPHFPDAYCNlAN
Da.re.GT41	DRAVAgYlRAlsLsPNHAVvHGNLACVYYEQGLIDLAIDTYRRAIELQPHFPDAYCNlAN
te.n.GT41	DRAVAgYlRAlsLsPNHAVvHGNLACVYYEQGLIDLAIDTYRRAIELQPHFPDAYCNlAN
Dm.G.OGT	DRAVAaYlRAlnLsPNNAVvHGNLACVYYEQGLIDLAIDTYRRAIELQPnFPDAYCNlAN

```
a.g.GT41_O    DRAVAaYlRAlnLsPNNAVvHGNLACVYYEQGLIDLAIDTYRRAIdLQhnFPDAYCNlAN
Ae.a.GT41     -KAVAaYlRAlnLsPyNAVvHGNLACVYYEQGLIDLAIDTYRRAIELQPnFPDAYCNlAN
bG1           DRAVAaYlRAlnLsPNNAVvHGNLACVYYEQGLIDLAIDTYRRAIELQqnFPDAYCNlAN
Ap.me.GT41    DRAVAaYlRAlnLsPNNAVvHGNLACVYYEQGLIDLAIDTYRRAIELQPnFPDAYCNlAN
ApG2          DRAVAaYlRAlnLsPNNAVvHGNLACVYYEQGLIDLAIDTYRRAIELQPnFPDAYCNlAN
Da.re.AGO6    ----dRicRfdyLCycteaee-------fvf-----------------------fhsnAs
te.n.AGO61    ----dRicRfdyLCycseaee-------fvf-----------------------fhsnss
ta.ru.AGO6    ----dRicRfdyLCycseaee-------fvf-----------------------fhsnss
M.AGO61       ----dRicRfkwLCySNeaee-------fif-----------------------fhgnss
R.AGO61       ----dRicRfkwLCySNeaee-------fif-----------------------fhgnss
H.AGO61       ----dRicRfkwLCySNeaee-------fif-----------------------fhgnts
e.c.AGO61     ----dRicRfkwLCySNeaee-------fif-----------------------fhgnts
S.AGO61       ----dRicRfkwLCySseaee-------fif-----------------------fhgnAs
B.AGO61       ----dRlcRfkwLCySseaee-------fif-----------------------fhgnAs
G.AGO611      ----dRicRfesLCySteaee-------fiy-----------------------fhsnss
x.t.AGO61     ----dRvcRfesLCySteaee-------fvf-----------------------fhsnss
X.l.AGO61     ----dRvcRfesLCySteaee-------fvf-----------------------fhsnAs
ta.ru.eOGT    gyvkeRlselkaLCkakkLggssLkCssYtr-----------------------fCkatN
te.n.eOGT     gyvkeRlselktLCkatkpvslahflIthtr-----------------------fCkttN
X.l.eOGT      gyvkeRlaetqiLCrpqeqgdsmLACsqnlQ-----------------------hCratN
x.t.eOGT      gyvkeRlaetqiLCrpqeqgdsmLACsrnlQ-----------------------hCratN
G.eOGT        gyireRlnemkthCkptvtgdssLtCsqflQ-----------------------hCratN
M.eOGT        gyAreRlgeirtiCqperasdssLvCsrYlQ-----------------------YCratg
R.eOGT        gyAreRleeirmfCrpeSasdssLlCsrYlQ-----------------------YCratg
S.eOGT        gyAgeRleelhvLCqaeeMndssLvCsrYlQ-----------------------YCratN
B.eOGT        gyAaeRleelhvLCqpkekndssLvCsrYlQ-----------------------YCratN
H.eOGT        gyAreRleemhvLCqpketsdssLvCsrYlQ-----------------------YCratN
e.c.eOGT      gyAgeRlgelrvLCrpeeVndssLvCsrYlQ-----------------------YCraAN
Dm.eOGT       gyiqeqlsqltpqCvptyLgdssLeCtHYlr-----------------------fCrgrN
a.g.eOGT      gfvrdqmretrimCepqfphdssLeCskYlr-----------------------fCrgrN
Ae.a.eOGT     gfvrdqiretrimCeptfphdsaLeCskYlr-----------------------fCrgrN
Ap.me.eOGT    gyvrdqrkemsifCeplfVddssLeCsehmr-----------------------fCrarN
```

```
b.mo.eOGT       ------------------------------------------------------------

AeO2            sLIalgrcQEAas------vL-----------------rEgsklDgvglrdraAhD--na
Ae.a.OGTli      ------------------------------------------------------------
b.mo.GT41       ---------------RYtqnL-------------gNvKrEqGkIDEatnLYmKALEVsli
H.G.OGT2        ALkEkgsVaEAED--cYNTALRLCPTHADSLNNLANIKrEqGNIEEavRLYrKALEVfPE
X.l.GT41_O      ALkEkgsVvDAEe--cYNTALRLCPTHADSLNNLANIKrEqGNIEEavRLYrKALEVfPE
x.t.GT41_O      ALkEkgsVvDAEe--cYNTALRLCPTHADSLNNLANIKrEqGNIEEavRLYrKALEVfPE
G.G.OGT         ALkEkgsVaEAEe--cYNTALRLCPTHADSLNNLANIKrEqGNIEEavRLYrKALEVfPE
M.G.OGT         ALkEkgsVaEAED--cYNTALRLCPTHADSLNNLANIKrEqGNIEEavRLYrKALEVfPE
B.G.OGT         ALkEkgsVaEAED--cYNTALRLCPTHADSLNNLANIKrEqGNIEEavRLYrKALEVfPE
H.G.OGT1        ALkEkgsVaEAED--cYNTALRLCPTHADSLNNLANIKrEqGNIEEavRLYrKALEVfPE
S.G.OGT         ALkEkgsVaEAED--cYNTALRLCPTHADSLNNLANIKrEqGNIEEavRLYrKALEVfPE
eG1             ALkEkgsVaEAED--cYNTALRLCPTHADSLNNLANIKrEqGNIEEavRLYrKALEVfPE
e.c.GT41_O      ALkEkgsVaEAED--cYNTALRLCPTHADSLNNLANIKrEqGNIEEavRLYrKALEVfPE
Da.re.GT41      ALkEkgnVsEAEe--cYNTALRLCPTHADSLNNLANIKrEqGNIEEavqLYrKALEVfPE
te.n.GT41       ALkEkgnVsEAEe--cYNTALRLCPTHADSLNNLANIKrEqGNIEEaiqLYrKALEVfPE
Dm.G.OGT        ALkEkgqVKEAED--cYNTALRLCsnHADSLNNLANIKrEqGyIEEatRLYlKALEVfPD
a.g.GT41_O      ALkEkgqVKEAEe--sYNiALRLCPnHADSLNNLANIKrEqGyIEEatRLYlKALEVfPE
Ae.a.GT41       ALkEkgqVQEAED--cYNTALRLCPnHADSLNNLANIKrEqGyIEEatRLYlKALEVfPE
bG1             ALkEkgqVtDAEe--cYNTALRLCPsHADSLNNLANIKrEqGyIEEatRLYlKALEVfPE
Ap.me.GT41      ALkEkgqVvEAED--cYNTALRLCPsHADSLNNLANIKrEqGyIEEatRLYlKALEVfPE
ApG2            ALkEkgqVvEAED--cYNTALRLCPsHADSLNNLANIKrEqGyIEEatRLYlKALEVfPE
Da.re.AGO6      vMLpnlGsR------RfqpAL-----------------LdlsSvEDHntqYfnfLELpaa
te.n.AGO61      vMLpnlGsR------RfqpAL-----------------LdlsSvEDHntqYfnfLELpaa
ta.ru.AGO6      vMLpnlGsR------RfqpAL-----------------LdlsSvEDHntqYfnfLELpaa
M.AGO61         vMLpnlGsR------RfqpAL-----------------LdlstvEDHnaqYfnfvELpaa
R.AGO61         vMLpnlGsR------RfqpAL-----------------LdlstvEDHnaqYfnfvELpaa
H.AGO61         vMLpnlGsR------RfqpAL-----------------LdlstvEDHntqYfnfvELpaa
e.c.AGO61       vMLpnlGsR------RfqpAL-----------------LdlstvEDHntqYfnfvELpaa
S.AGO61         vMLpslGsR------RfqpAL-----------------LdlstvEDHntqYfnfvELpaa
B.AGO61         vMLpslGsR------RfqpAL-----------------LdlstvEDHntqYfnfvELpaa
```

```
G.AGO611      vMLpnlGsR------RfqpAL-----------------LdlsSvEDHntqYfnfvELpaa
x.t.AGO61     iMLpnlGpR------RfqpAL-----------------LdlsSvDDHntqYfnfiELpaa
X.l.AGO61     iMLpnlGpR------RfqpAL-----------------LdlsSvDDHntqYfnfiELpaa
ta.ru.eOGT    lyLDlrkpRrShe--RYkedf-----------------IqkGelggHCRLnkaALaaegD
te.n.eOGT     lyLDlrepnrShe--RYkedf-----------------IErGelggYCRLnkaALaaegD
X.l.eOGT      lyLDlrhpRrgQe--nfkedf-----------------LqeGeIggHCdLdkqALlsqga
x.t.eOGT      lyLDlrnpRrgQe--nfkedf-----------------LqeGeIggrCnLdkqALlsqga
G.eOGT        lyIDlrtaKrnhe--Rfkedf-----------------fqkGeIggHCtLdvKAflaegQ
M.eOGT        lyLDlrnIKrnhD--Rfkedf-----------------LqgGeIggYCKLdshALvsegQ
R.eOGT        lyLDlrnIKrnhD--Rfkedf-----------------LqgGDIggYCKLdrhALvsegQ
S.eOGT        iyLDlrnIKrnhD--Rfkedf-----------------fqnGeIggHCKLdiRtLmsegQ
B.eOGT        iyLDlrnIKrnhD--Rfkedf-----------------VqsGeIggYCKLdiRSLmsqgQ
H.eOGT        lyLDlrnIKrnhD--Rfkedf-----------------fqsGeIggHCKLdiRtLtsegQ
e.c.eOGT      lyLDlrnIQrnhD--Rfkedf-----------------fqsGeIggHCKLdiRtLmsegQ
Dm.eOGT       lLfDfrGLeQrEeriRYhmdv-----------------LgpGqllgHCKLnrtrLsgemE
a.g.eOGT      lMLnftdLvhrtePLRYkmdv-----------------LgpGqIggHCRLhgerLrdelQ
Ae.a.eOGT     iMVnftdLihrtePLRYkmdv-----------------LshGqIggHCKLhrKrLEdelE
Ap.me.eOGT    iMInftdLirrkePiRYkmdv-----------------LkeGqIggYCtLneKrLEenaD
b.mo.eOGT     -MLnftGLvgrgDnLRYkTdi-----------------LsaGqIggYCKfYsdrLmkeaE

AeO2          ri-sAllqLgnLyadQGKLQ---------RalavYrEAlhilpdr----yppqGiyhrl
Ae.a.OGTli    ------------------------------------------------------------
b.mo.GT41     lr-qliaiwhpfysnk------------AIgiQPkFaDAYSNMgNTLRElQDMtGALaCf
H.G.OGT2      fA-AAHSNLASVLQQQGKLQEALMHYKEAIRisPTFaDAYSNMgNTLKEMQDVqGALQCY
X.l.GT41_O    fA-AAHSNLASVLQQQGKLQEALMHYKEAIRisPTFaDAYSNMgNTLKEMQDVqGALQCY
x.t.GT41_O    fA-AAHSNLASVLQQQGKLQEALMHYKEAIRisPTFaDAYSNMgNTLKEMQDVqGALQCY
G.G.OGT       fA-AAHSNLASVLQQQGKLQEALMHYKEAIRisPTFaDAYSNMgNTLKEMQDVqGALQCY
M.G.OGT       fA-AAHSNLASVLQQQGKLQEALMHYKEAIRisPTFaDAYSNMgNTLKEMQDVqGALQCY
B.G.OGT       fA-AAHSNLASVLQQQGKLQEALMHYKEAIRisPTFaDAYSNMgNTLKEMQDVqGALQCY
H.G.OGT1      fA-AAHSNLASVLQQQGKLQEALMHYKEAIRisPTFaDAYSNMgNTLKEMQDVqGALQCY
S.G.OGT       fA-AAHSNLASVLQQQGKLQEALMHYKEAIRisPTFaDAYSNMgNTLKEMQDVqGALQCY
eG1           fA-AAHSNLASVLQQQGKLQEALMHYKEAIRisPTFaDAYSNMgNTLKEMQDVqGALQCY
```

```
e.c.GT41_O      fA-AAHSNLASVLQQQGKLQEALMHYKEAIRisPTFaDAYSNMgNTLKEMQDVqGALQCY
Da.re.GT41      fA-AAHSNLASVLQQQGKLQEALMHYKEAIRisPTFaDAYSNMgNTLKEMQDVqGALQCY
te.n.GT41       fA-AAHSNLASVLQQQGKLQEALMHYKEAIRisPTFaDAYSNMgNTLKEMQDVqGALQCY
Dm.G.OGT        fA-AAHSNLASVLQQQGKLKEALMHYKEAIRiQPTFaDAYSNMgNTLKElQDVsGALQCY
a.g.GT41_O      fA-AAHSNLASVLQQQGKLnEALlHYKEAIRiQPTFaDAYSNMgNTLKEMQDVaGALQCY
Ae.a.GT41       fA-AAHSNLASVLQQQGKLnEALlHYKEAIRiQPTFaDAYSNMgNTLKEMQDVaGALQCY
bG1             fA-AAHSNLASVLQQQGKLnEALMHYKEAIRiQPTFaDAYSNMgNTLKEMQDVaGALQCY
Ap.me.GT41      fA-AAHSNLASVLQQQGKLnEALMHYKEAIRiQPTFaDAYSNMgNTLKEMQDIqGALQCY
ApG2            fA-AAHSNLASVLQQQGKLnEALMHYKEAIRiQPTFaDAYSNMgNTLKEMQDIqGALQCY
Da.re.AGO6      al---------------Kfm-----------pKPvFvpdvtlilNr----fnpDnlmhif
te.n.AGO61      tl---------------rfl-----------pKPvFvpdvalilNr----fnpDnlmhvf
ta.ru.AGO6      tl---------------rfm-----------pKPvFvpdvtlilNr----fnpDnlmhvf
M.AGO61         al---------------rfm-----------pKPvFvpdvaliaNr----fnpDnlmhvf
R.AGO61         al---------------rfm-----------pKPvFvpdvaliaNr----fnpDnlmhvf
H.AGO61         al---------------rfm-----------pKPvFvpdvaliaNr----fnpDnlmhvf
e.c.AGO61       al---------------rfm-----------pKPvFvpdvaliaNr----fnpDnlmhvf
S.AGO61         al---------------rfm-----------pKPvFvpdvaliaNr----fnpDnlmhvf
B.AGO61         al---------------rfm-----------pKPvFvpdvaliaNr----fnpDnlmhvf
G.AGO611        al---------------Kfm-----------pKPvFvpdvaliaNr----fnpDnlmhvf
x.t.AGO61       al---------------Kfm-----------pKPvFvpdvalimNr----fnpDnlmhvf
X.l.AGO61       al---------------Kfm-----------pKPvFvpdvalimNr----fnpDnlmhvf
ta.ru.eOGT      hk-splqswyaeLQtyteLd-----------sdPivngqcdltvdkptvfmkLDagvnmY
te.n.eOGT       hk-splqswyaeLQtyteLd-----------sdPivngqcdltvdkptvfmkLDagvnmY
X.l.eOGT        wk-splqswfaeLQsyssfK-----------fKPiedahcdiiiekptyfmkLDagvnmY
x.t.eOGT        wk-splqswfaeLQsyssLt-----------fKPvedahcdiiidkptyfmkLDagvnmY
G.eOGT          rk-splqswfaeLQtftsLn-----------fRPlddgkcdiviekptyfmkLDagvnmY
M.eOGT          rk-splqswfaeLQgytqLn-----------fRPiedakcdivvekptyfmkLDaginmY
R.eOGT          rk-splqswfaeLQgytqLn-----------fRPiedakcdivvekptyfmkLDaginmY
S.eOGT          rk-splqswfaeLQsytqLn-----------fRPiedaAcdiviekptyfmkLDagvnmY
B.eOGT          rk-splqswfaeLQsyteLn-----------fRPvedaqcdiviekptyfmkLDagvnmY
H.eOGT          rk-splqswfaeLQsytqLn-----------fRPiedakcdiviekptyfmkLDagvnmY
e.c.eOGT        rk-splqswfaeLQsytqLp-----------fRPiedakcdiviekptyfmkLDagvnmY
```

```
Dm.eOGT         higsAlqswgpeLrnfdvLp-----------hpvlesglcdvvvNTptfimkIDatynmY

a.g.eOGT        hi-splqswgpeLrfferLE-----------rpPietgvcdvvierpafimkIDaAinmY

Ae.a.eOGT       hi-splqswgpeLrffdtvd-----------kplsqggtcdvtidrpafimkIDatinmY

Ap.me.eOGT      hi-splqswgpeLrnfrKLs-----------rpPivnhdcdiviekptfvmkIDaivnmY

b.mo.eOGT       hm-sAlqswgpemvnfvKtp-----------kKPiadgmcdividkptyimkLDag----


AeO2            gevfarlnqwSeAerf----qraaleA--qPdhIaAh----IsyGsmlarnssrtseaeQ

Ae.a.OGTli      ------------------------------------------------------------

b.mo.GT41       kKAIeINPtFSdAHcNL---ASIYKDTGNIkEAIeSYKnA-LyfkPdFPDAYcnLAHClQ

H.G.OGT2        tRAIqINPaFAdAHSNL---ASIHKDSGNIPEAIaSYRTA-LKLkPdFPDAYcnLAHClQ

X.l.GT41_O      tRAIqINPaFAdAHSNL---ASIHKDSGNIPEAIaSYRTA-LKLkPdFPDAYcnLAHClQ

x.t.GT41_O      tRAIqINPaFAdAHSNL---ASIHKDSGNIPEAIaSYRTA-LKLkPdFPDAYcnLAHClQ

G.G.OGT         tRAIqINPaFAdAHSNL---ASIHKDSGNIPEAIaSYRTA-LKLkPdFPDAYcnLAHClQ

M.G.OGT         tRAIqINPaFAdAHSNL---ASIHKDSGNIPEAIaSYRTA-LKLkPdFPDAYcnLAHClQ

B.G.OGT         tRAIqINPaFAdAHSNL---ASIHKDSGNIPEAIaSYRTA-LKLkPdFPDAYcnLAHClQ

H.G.OGT1        tRAIqINPaFAdAHSNL---ASIHKDSGNIPEAIaSYRTA-LKLkPdFPDAYcnLAHClQ

S.G.OGT         tRAIqINPaFAdAHSNL---ASIHKDSGNIPEAIaSYRTA-LKLkPdFPDAYcnLAHClQ

eG1             tRAIqINPaFAdAHSNL---ASIHKDSGNIPEAIaSYRTA-LKLkPdFPDAYcnLAHClQ

e.c.GT41_O      tRAIqINPaFAdAHSNL---ASIHKDSGNIPEAIaSYRTA-LKLkPdFPDAYcnLAHClQ

Da.re.GT41      tRAIqINPaFAdAHSNL---ASIHKDSGNIPEAIaSYRTA-LKLkPdFPDAYcnLAHClQ

te.n.GT41       tRAIqINPaFAdAHSNL---ASIHKDSGNIPEAIaSYRTA-LKLkPdFPDAYcnLAHClQ

Dm.G.OGT        tRAIqINPaFAdAHSNL---ASIHKDSGNIPEAIqSYRTA-LKLkPdFPDAYcnLAHClQ

a.g.GT41_O      tRAIqINPaFAdAHSNL---ASIHKDSGNIPEAIqSYRTA-LKLkPdFPDAYcnLAHClQ

Ae.a.GT41       tRAIqINPaFAdAHSNL---ASIHKDSGNIPdAIqSYRTA-LKLkPdFPDAYcnLAHClQ

bG1             tRAIqINPaFAdAHSNL---ASIHKDSGNIPEAIqSYRTA-LKLkPdFPDAYcnLAHClQ

Ap.me.GT41      tRAIqINPaFAdAHSNL---ASIHKDSGNIPEAIqSYRTA-LKLkPdFPDAYcnLAHClQ

ApG2            tRAIqINPaFAdAHSNL---ASIHKDSGNIPEAIqSYRTA-LKLkPdFPDAYcnLAHClQ

Da.re.AGO6      Hd--DLlPvyyTmqqy----SdLddeA-----rlvfmeg--wgeGahF-DlYRlLsskqp

te.n.AGO61      Hd--DLlPaFyTmkqf----ldLdEDA-----rlvfmeg--wdeGPhF-hlYRlLsdkqp

ta.ru.AGO6      Hd--DLlPaFyTmkqf----ldsdEDA-----rlvfmeg--wEeGPhF-elYRlLsnkqp

M.AGO61         Hd--DLlPLFyTlrqf----pgLaQeA-----rlffmeg--wgeGahF-DlYKlLspkqp

R.AGO61         Hd--DLlPLFyTlrqf----pgLaQeA-----rlffmeg--wgeGahF-DlYKlLspkqp
```

```
H.AGO61        Hd--DLlPLFyTlrqf----pgLaheA-----rlffmeg--wgeGahF-DlYKlLspkqp
e.c.AGO61      Hd--DLlPLFyTlrqf----pgLaQeA-----rlffmeg--wgeGahF-DlYKlLspkqp
S.AGO61        Hd--DLlPLFyTlrqf----pgLaReA-----rlffmeg--wgeGahF-DlYKlLspkqp
B.AGO61        Hd--DLlPLFyTlrqf----pgLaReA-----rlffmeg--wgeGahF-DlYKlLspkqp
G.AGO611       Hd--DLlPIyyTmqqf----tdLdpeT-----rlffmeg--wseGvhF-DlYKlLsnkqp
x.t.AGO61      Hd--DLiPIFyTiqqf----AdLdfeS-----rlffmeg--wneGlhF-elYKfmsnkqp
X.l.AGO61      Hd--DLlPIFyTiqqf----pdLdfeS-----rlffmeg--wneGlhF-elYKfmsnkqp
ta.ru.eOGT     HhfcDfvnLyisqHiN----nSfssDi-----nIvmwdTsSyEyGdlFsetwRafsqndi
te.n.eOGT      HhfcDfvnLyisqHiN----nSfssDi-----nIvmwdTsfyEyGdlFsetwRafsendi
X.l.eOGT       HhfcDfvnLyiTqHvN----nSfstDi-----nIvmwtTsvygyGdlFsDtwKaftdyei
x.t.eOGT       HhfcDfvnLyiTqHvN----nSfstDi-----nIvmwtTsvygyGdlFsDtwKaftdydi
G.eOGT         HhfcDfvnLyiTqHiN----nSfstDv-----nIvmwdTsSygyGdlFsetwKaftdydi
M.eOGT         HhfcDflnLylTqHvN----nSfstDv-----yIvmwdTsTygyGdlFsDtwKaftdydv
R.eOGT         HhfcDflnLylTqHiN----nSfstDv-----yIvmwdTsSygyGdlFsDtwKaftdydv
S.eOGT         HhfcDfvnLyiTqHvN----nSfstDv-----yvvmwdTsSygyGdlFsDtwKaftdydv
B.eOGT         HhfcDfinLyiTqHvN----nSfstDv-----yvvmwdTsSygyGdlFsDtwKaftdydv
H.eOGT         HhfcDfinLyiTqHvN----nSfstDv-----yIvmwdTsSygyGdlFsDtwnaftdydv
e.c.eOGT       HhfcDfinLyiTqHvN----nSfstDv-----hIvmwdTsSygyGdlFsDtwKaftdydv
Dm.eOGT        HhfcDffnLyAslfvNqSHPAafntDv-----qIliweT--ypydspFrDtfKafsqrpv
a.g.eOGT       HhfcDflnLyAslHvNLSHaggfdtDT-----qvlvwes--ftyaspFaDtfKvfskhpi
Ae.a.eOGT      HhfcDfinLygslHANLSdPygfstDv-----qImvwes--ytydspFaetfKvftkhpi
Ap.me.eOGT     HhfcDffnLyAslHvNLSHPAgfstDN-----hImiwes--ysyrsaFqDAfeaftrnpl
b.mo.eOGT      -----------------------------------weT--ftydsaFkDAfKaftenpi

AeO2           wfk----------RalrlApADasvhHhyaef-------------------lAsvRRtAe
Ae.a.OGTli     ------------------------------------------------------------
b.mo.GT41      IVC-nwdDYyeRMhniIeiVnkqLkmDKLs---SVHPHHSiLYPLtneaR-ReIAtRhAN
H.G.OGT2       IVC-DwtDYDeRMKklVSiVADqLeKNRLP---SVHPHHSMLYPLshGfR-KAIAeRhgN
X.l.GT41_O     IVC-DwtDYDeRMKklVSiVAEqLeKNRLP---SVHPHHSMLYPLshafR-KAIAeRhgN
x.t.GT41_O     IVC-DwtDYDeRMKklVSiVADqLeKNRLP---SVHPHHSMLYPLshGfR-KAIAeRhgN
G.G.OGT        IVC-DwtDYDeRMKklVSiVADqLeKNRLP---SVHPHHSMLYPLshsfR-KAIAeRhgN
M.G.OGT        IVC-DwtDYDeRMKklVSiVAEqLeKNRLP---SVHPHHSMLYPLshGfR-KAIAeRhgN
```

```
B.G.OGT      IVC-DwtDYDeRMKklVSiVADqLeKNRLP---SVHPHHSMLYPLshGfR-KAIAeRhgN
H.G.OGT1     IVC-DwtDYDeRMKklVSiVADqLeKNRLP---SVHPHHSMLYPLshGfR-KAIAeRhgN
S.G.OGT      IVC-DwtDYDeRMKklVSiVADqLeKNRLP---SVHPHHSMLYPLshGfR-KAIAeRhgN
eG1          IVC-DwtDYDeRMKklVSiVADqLeKNRLP---SVHPHHSMLYPLshGfR-KAIAeRhgN
e.c.GT41_O   IVC-DwtDYDeRMKklVSiVADqLeKNRLP---SVHPHHSMLYPLshGfR-KAIAeRhgN
Da.re.GT41   IVC-DwtDYDeRMKklVSiVADqLeKNRLP---SVHPHHSMLYPLshGfR-KAIAeRhgN
te.n.GT41    IVC-DwtDYDeRMKklVTiVADqLdKNRLP---SVHPHHSMLYPLshGfR-KAIAeRhgN
Dm.G.OGT     IVC-DwtDYDiRMKklVSiVtEqLeKNRLP---SVHPHHSMLYPLthdCR-KAIAaRhAN
a.g.GT41_O   IVC-DwtDYeaRMKklVAiVADqLdKNRLP---SVHPHHSMLYPLshefR-KtIAaRhAN
Ae.a.GT41    IVC-DwtDYeaRMKklVAiVADqLeKNRLP---SVHPHHSMLYPLshdfR-KAIAaRhAN
bG1          IVC-DwtDYeaRMKklVSiVAEqLeKNRLP---SVHPHHSMLYPLthdfR-KAIAaRhAN
Ap.me.GT41   IVC-DwtDYeaRMKklVSiVAEqLdKNRLP---SVHPHHSMLYPLshefR-KAIAaRhAN
ApG2         IVC-DwtDYeaRMKklVSiVAEqLdKNRLP---SVHPHHSMLYPLshefR-KAIAaRhAN
Da.re.AGO6   LlkdqLKtf-GkLmCFtkSyvgLskmttwyqYGfVqPqgpkaniLISGne----iRqFAs
te.n.AGO61   LlkeqLRnf-GkLmCFtkSyigLskmttwyqYGfVqPqgpkaniLVSGne----iRhFAk
ta.ru.AGO6   LlkeqLRnf-GkLmCFtkSyigLskmttwyqYGfVqPqgpkaniLVSGne----iRhFAk
M.AGO61      LlrAqLKtl-GRLlCFshAfvgLskvttwyqYGfVqPqgpkaniLVSGne----iRqFtr
R.AGO61      LlrsqLKtl-GRLlCFshAfvgLskvttwyqYGfVqPqgpkaniLVSGne----iRqFtr
H.AGO61      LlrAqLKtl-GRLlCFshAfvgLskittwyqYGfVqPqgpkaniLVSGne----iRqFAr
e.c.AGO61    LlrAqLKtl-GRLlCFshAfvgLskittwyqYGfVqPqgpkaniLVSGne----iRqFAr
S.AGO61      LlrAqLKal-GRLlCFshAfvgLskvttwyqYGfVqPqgpkaniLVSGne----iRqFAr
B.AGO61      LlrAqLKal-GRLlCFshAfvgLskvttwyqYGfVqPqgpkaniLVSGne----iRqFAh
G.AGO611     LlreqLKtl-GRLlCFtkSyvgLskittwyqYGfVqPqgpkaniLVSGne----iRqFtk
x.t.AGO61    LlkeqLKtl-GRLlCFtkSyvgLskittwyqYGfVqPqgpkaniLVSGne----iRhFAk
X.l.AGO61    LlkeqLKtl-GRLlCFtkSyvgLskittwyqYGfVqPqgpkaniLVSGne----iRhFAk
ta.ru.eOGT   I---hLKvYDnkrvCFrdAlfsLLPRmRygLf-yntP-------LISdCysegmfRaFsq
te.n.eOGT    I---hLKtYDskrvCFrdAffsLLPRmRygLf-yntP-------LISdCysegmfRaFsq
X.l.eOGT     t---hLKaYDnkrvCFkdAVfaLLPRmRygLf-yntP-------LIShChgsglfRaFsq
x.t.eOGT     t---hLKaYDnkrvCFkdAVfaLLPRmRygLf-yntP-------LISnChgsglfRaFsq
G.eOGT       I---yLKtfDskrvCFkeAVfsLLPRmRygLf-yntP-------LISGChgtglfRaFsq
M.eOGT       I---hLKtYDskkvCFkeAVfsLLPRmRygLf-yntP-------LISGCqNtglfRaFsq
R.eOGT       I---hLKtYDskkvCFkeAVfsLLPRmRygLf-yntP-------LISGCqNtglfRaFsq
```

```
S.eOGT        I---hLKtYDskrvCFkeAIfsLLPRmRygLf-yntP-------LISGCqNtglfRaFsq
B.eOGT        I---hLKtYDakrvCFkeAIfsLLPRmRygLf-yntP-------LISGCqNtglfRaFsq
H.eOGT        I---hLKtYDskrvCFkeAVfsLLPRmRygLf-yntP-------LISGCqNtglfRaFAq
e.c.eOGT      I---hLKtYDskrvCFkeAVfsLLPRmRygLf-yntP-------LISGCqNtglfRaFsq
Dm.eOGT       w---tLsDveGkrvCFkNvVlpLLPRmifgLf-yntP-------iIqGCsNsglfRaFse
a.g.eOGT      a---DLKtYaGkvvCFkNvVlpLLPRmifgLY-yntP-------iIyGCeNsglfhaFse
Ae.a.eOGT     a---DLKtYaGkvvCFkNlVlpLLPRmifgLY-yntP-------iItGCeNsgffqaFse
Ap.me.eOGT    w---DLKtfrGetvCFkNlVfpLLPRmifgLY-yntP-------LIyGCeNsglfKaFge
b.mo.eOGT     w---DLKrfrGkvvCFkNAVfpLLPRmifgLY-yntP-------LIyGCersglfhaFsk

AeO2          aCqYrvka-----aelaPdD----------------------yslVtaaasalrlLdrkv
Ae.a.OGTli    ------------------------------------------------------------
b.mo.GT41     LyidKLhfLqntvtFQhPKvme---GR----LRIGYvSsDFGNhPtshLmqsIpgsHDrs
H.G.OGT2      LCLdKiNVLHKP-PYEhPKDLKLSDGR----LRvGYvSsDFGNhPtshLmqsIpgmHNpD
X.l.GT41_O    LCLdKiNVLHKP-PYEhPKDLKaSDGR----LRvGYvSsDFGNhPtshLmqsIpgmHNpD
x.t.GT41_O    LCLdKiNVLHKP-PYEhPKDLKaSDGR----LRvGYvSsDFGNhPtshLmqsIpgmHNpD
G.G.OGT       LCLdKiNVLHKP-PYEhPKDLKaSeGR----LRIGYvSsDFGNhPtshLmqsIpgmHNpD
M.G.OGT       LCLdKiNVLHKP-PYEhPKDLKLSDGR----LRvGYvSsDFGNhPtshLmqsIpgmHNpD
B.G.OGT       LCLdKiNVLHKP-PYEhPKDLKLSDGR----LRvGYvSsDFGNhPtshLmqsIpgmHNpD
H.G.OGT1      LCLdKiNVLHKP-PYEhPKDLKLSDGR----LRvGYvSsDFGNhPtshLmqsIpgmHNpD
S.G.OGT       LCLdKiNVLHKP-PYEhPKDLKLSDGR----LRvGYvSsDFGNhPtshLmqsIpgmHNpD
eG1           LCLdKiNVLHKP-PYEhPKDLKLSDGR----LRvGYvSsDFGNhPtshLmqsIpgmHNpD
e.c.GT41_O    LCLdKiNVLHKP-PYEhPKDLKLSDGR----LRvGYvSsDFGNhPtshLmqsIpgmHNpD
Da.re.GT41    LCLdKiNaLHKP-aYEhPKDLKaSsGR----LRvGYISsDFGNhPtshLmqsIpgmHNsE
te.n.GT41     LCLdKiNaLHKP-PYEhPKDLKaSgGR----LRvGYvSsDFGNhPtshLmqsIpgmHNpE
Dm.G.OGT      LCLeKvhVLHKk-PYnflKkLptk-GR----LRIGYLSsDFGNhPtshLmqsVpgLHDrs
a.g.GT41_O    LCLeKiNVLHKP-PYKfgREL--S-GR----LRIGYvSsDFGNhPtshLmqsIpgLHDrN
Ae.a.GT41     LCLeKihILHKP-PYKfsRDm--S-qR----LRIGYvSsDFGNhPtshLmqsIpgmHDrs
bG1           LCLeKvqVLHKn-PYKfPRDL--q-GR----LRIGYvSsDFGNhPtshLmqsVpgLHDrt
Ap.me.GT41    LCieKihVLHKq-PYKyPREig---GR----LRIGYvSsDFGNhPtshLmqsIpgLHerq
ApG2          LCieKihVLHKq-PYKyPREig---GR----LRIGYvSsDFGNhPtshLmqsIpgLHerq
Da.re.AGO6    flmerLNI-----trEeeeE-----dd----dyIvvfkRttn-RlILNeaELllALaqe-
```

```
te.n.AGO61      VlmeKmNV-----traeggq-----ed----eyIvvfSRsst-RlILNqaELVmALaqe-
ta.ru.AGO6      VlmeKmNItr---aaggeKDqgnaedekpkdeyIvvfSRstt-RlILNeaELImALaqe-
M.AGO61         fmterLNV-----sHagapl-----Ge----eyIlvfSRtqn-RlILNeaELlleLaqe-
R.AGO61         fmterLNV-----sHagapl-----Ge----eyIlvfSRtqn-RlILNeaELlleLaqe-
H.AGO61         fmteKLNV-----sHtgvpl-----Ge----eyIlvfSRtqn-RlILNeaELllALaqe-
e.c.AGO61       fmteKLNV-----sHtgapl-----Ge----eyIlvfSRtqn-RlILNeaELllALaqe-
S.AGO61         flteKLNV-----sHaggal-----Ge----eyIlvfSRtqn-RlILNeaELllALaqe-
B.AGO61         flmeKLNV-----sqaggpl-----Ge----eyIlvfSRtqn-RlILNeaELllALaqe-
G.AGO611        fmmqKLNV-----slEess------se----eyIvvfSRtin-RlILNeaELIlALaqe-
x.t.AGO61       fmmgKLNI-----tkDqna------ae----ayIvlfSRsmn-RlIVNeaELllALaqe-
X.l.AGO61       fmmgKLNI-----tlDqna------ae----ayIvlfSRsmn-RlIVNeaELllALaqe-
ta.ru.eOGT      hiLHrLhV-----PqDgPKD-----GR----VRvtlLaRsteyRkILNqvELVnALktvp
te.n.eOGT       hvLHrLNI-----PqDgPKD-----GR----VRvtlLaRsteyRkILNqvELVnALktvp
X.l.eOGT        hvLHrLNI-----tqhpatE-----ak----IRvtiLvRstefRkILNLdELVqALeavp
x.t.eOGT        hvLHrLNI-----tqQlPKE-----ak----IRItiLvRstefRkILNLdELVhALeaep
G.eOGT          hvLHrLNI-----tqEgPKD-----Gk----IRvtiLaRstdyRkILNqnELVnALktvs
M.eOGT          hvLHrLNI-----tqEgPKD-----Gk----VRvtiLaRsteyRkILNqdELVnALktvs
R.eOGT          hvLHrLNI-----sqEgPKD-----Gk----LRvtiLaRsteyRkILNqnELVnALktvs
S.eOGT          hvLHrLNI-----tqQgPKD-----Gk----IRvtiLaRsteyRkILNqnELVnALktvs
B.eOGT          hvLHrLNI-----tqEgPKg-----Gk----IRvtiLaRsteyRkILNqnELVnALktvs
H.eOGT          hvLHrLNI-----tqEgPKD-----Gk----IRvtiLaRsteyRkILNqnELVnALktvs
e.c.eOGT        hvLHrLNI-----tqEgPKD-----Gk----IRvtiLaRsteyRkILNqnELVnALktvs
Dm.eOGT         fiLHrLqI-----PYKpPq------qk----IRItYLSRrtkyRqVLNedELlapLeanD
a.g.eOGT        hvLHrLkV-----rmttrpD-----eR----VRItFLSRqtryRrVLNedELmgriaknp
Ae.a.eOGT       hvLHrLkV-----PqrsrsD-----rk----LRItFLSRqtkfRrVLNenaLleeisenE
Ap.me.eOGT      hvLHrLrI-----PlherKn-----qR----IRvtlLSRDtqyRrILNedELVrALkenp
b.mo.eOGT       hiLHsLNI-----klhmrtD-----dR----VRvtlLSRgttyRsILNekEIVdALlkeD

AeO2            EaErw-----------YRqaVTlrpeearshTnl--------------------------
Ae.a.OGTli      ------------------------------------------------------------
b.mo.GT41       KvEVFCYALnaDDGTtFRrKIveESeNFIDLScIPCNmeAAtRIHrDGIHILiNMnGYTK
H.G.OGT2        KFEVFCYALSPDDGTNFRVKVmaEAnHFIDLSQIPCNGKAADRIHQDGIHILVNMnGYTK
```

```
X.l.GT41_O     KFEVFCYALSPDDGTNFRVKVmaEAnHFVDLSQIPCNGKAADRIHQDGIHILVNMnGYTK
x.t.GT41_O     KFEVFCYALSPDDGTNFRVKVmaEAnHFVDLSQIPCNGKAADRIHQDGvHILiNMnGYTK
G.G.OGT        KFEVFCYALSPDDGTNFRVKVmaEAnHFVDLSQIPCNGKAADRIHQDGIHILiNMnGYTK
M.G.OGT        KFEVFCYALSPDDGTNFRVKVmaEAnHFIDLSQIPCNGKAADRIHQDGIHILVNMnGYTK
B.G.OGT        KFEVFCYALSPDDGTNFRVKVmaEAnHFIDLSQIPCNGKAADRIHQDGIHILVNMnGYTK
H.G.OGT1       KFEVFCYALSPDDGTNFRVKVmaEAnHFIDLSQIPCNGKAADRIHQDGIHILVNMnGYTK
S.G.OGT        KFEVFCYALSPDDGTNFRVKVmaEAnHFIDLSQIPCNGKAADRIHQDGIHILVNMnGYTK
eG1            KFEVFCYALSPDDGTNFRVKVmaEAnHFIDLSQIPCNGKAADRIHQDGIHILVNMnGYTK
e.c.GT41_O     KFEVFCYALSPDDGTNFRVKVmaEAnHFIDLSQIPCNGKAADRIHQDGIHILVNMnGYTK
Da.re.GT41     KFEVFCYALSPDDGTNFRVKVmaEAhHFIDLSQIPCNGKAADRIHQDGIHILVNMnGYTK
te.n.GT41      KFEVFCYALSPDDsTNFRVKVvaEAhHFVDLSQIsCNGKAADRIHQDGvHILVNMnGYTK
Dm.G.OGT       KvEIFCYALSPDDGTtFRhKISrESeNFVDLSQIPCNGKAADkIfnDGIHILVNMnGYTK
a.g.GT41_O     RvEIFCYALSPDDGTtFRgKISrEAeHFIDLSQtPCNGKAADRIHaDGIHILVNMnGYTK
Ae.a.GT41      RvEVFCYALSPDDGTtFRsKISrEAeHFIeLSQIPCNGKAADRIHaDGIHILVNMnGYTK
bG1            KvEIFCYALSPDDGTtFRsKIArEAeHFIDLSQmPCNGKAADkIygDGIHILVNMnGYTK
Ap.me.GT41     nvEIFCYALSaDDGTtFRaKIArEteHFVDLSQIPCNGKAADRInaDGIHILVNMnGYTK
ApG2           nvEIFCYALSaDDGTtFRaKIArEteHFVDLSQIPCNGKAADRInaDGIHILVNMnGYTK
Da.re.AGO6     -Fqm------------rtVtVSlEeqsFdnIiQIisraa-----------mLVsMHG---
te.n.AGO61     -Fqm------------rvVtVSlEeqsFasIvQVigaas-----------mLVsMHG---
ta.ru.AGO6     -Fqm------------rvVtVSlEeqsFpsIvQVisgas-----------mLVsMHG---
M.AGO61        -Fqm------------ktVtVSlEdhtFaDVvrlvsNas-----------mLVsMHG---
R.AGO61        -Fqm------------ktVtVSlEdhtFaDVvrlvsNas-----------mLVsMHG---
H.AGO61        -Fqm------------ktVtVSlEdhtFaDVvrlvsNas-----------mLVsMHG---
e.c.AGO61      -Fqm------------ktVtVSlEdyaFaDVvrlvsNas-----------mLVsMHG---
S.AGO61        -Fqm------------ktVtVSlEdhaFaDVvrlvsNas-----------mLVsMHG---
B.AGO61        -Fqm------------ktVtVSlEdhaFaDVvrlvsNas-----------mLVsMHG---
G.AGO611       -Fqm------------ktItVSlEehsFsDIvrlisNas-----------mLVsMHG---
x.t.AGO61      -Fqm------------ktItVSlEdhsFaDIvrlisNat-----------mLVsMHG---
X.l.AGO61      -Fqm------------ktItVSlEdhsFsDIvrllsNat-----------mLVsMHG---
ta.ru.eOGT     nlEV------------nvVdykykdvpFLvqlktthNsd-----------IfigMHG---
te.n.eOGT      hlkV------------nvVdfkykdvpFLvqlkIthNsd-----------IfigMHG---
X.l.eOGT       tFqV------------kvVdykyrvlgFLeqlsIthNsd-----------IfigMHG---
```

```
x.t.eOGT       tFqV------------kvVdykyrvlgFLeqlEIthNsd-----------IfigMHG---
G.eOGT         tlEV------------kvVdykykeleFseqlrIthNsd-----------IfigMHG---
M.eOGT         tFEV------------rvVdykyrelgFLDqlrIthNtd-----------IfigMHG---
R.eOGT         tFEV------------rvVdykyrelgFLDqlrIthNtd-----------IfigMHG---
S.eOGT         tFEV------------riVdykykelaFLDqlrIthNtd-----------IfigMHG---
B.eOGT         tFEV------------qiVdykykelgFLDqlrIthNtd-----------IfigMHG---
H.eOGT         tFEV------------qiVdykyrelgFLDqlrIthNtd-----------IfigMHG---
e.c.eOGT       tFEV------------qiVdykykqlgFLDqlrIthNtd-----------IfigMHG---
Dm.eOGT        KYdV--------------qRVSyErlpFtnqlaItrNtd-----------ILigMHG---
a.g.eOGT       nYaV------------qRVsyg-hdlpFVeqlrItrNtd-----------IfigMHG---
Ae.a.eOGT      dYlV------------nQasfTyk-tdFreqlkItrNtd-----------IfigMHG---
Ap.me.eOGT     lYkV------------kKVvyN-kkvsFkkqlEItrNsd-----------IfigiHG---
b.mo.eOGT      gYyV------------qRVvyd-rtvsFtkqlEIthNtd-----------VfigMHG---


AeO2           ----------------------------------------------------gailHlLg
Ae.a.OGTli     ------------------------------------------------------------
b.mo.GT41      GARNEiFALkPAPIQvMWLGYPGTSGAgyiDYmITDEvsaPlslsEdfSEKfAYmPHTyF
H.G.OGT2       GARNELFALRPAPIQAMWLGYPGTSGAlFMDYIITDQETSPAEVAEQYSEKLAYmPHTfF
X.l.GT41_O     GARNELFALkaAPIQAMWLGYPGTSGApFMDYIIsDKETSPidVAEQYSEKLAYmPnTfF
x.t.GT41_O     GARNELFALkaAPIQAMWLGYPGTSGAsFMDYIIsDiETSPvdVAEQYSEKLAYmPnTfF
G.G.OGT        GARNELFALRPAPIQAMWLGYPGTSGAlFMDYIITDKETSPvEVAEQYSEKLAYmPnTfF
M.G.OGT        GARNELFALRPAPIQAMWLGYPGTSGAlFMDYIITDQETSPAEVAEQYSEKLAYmPHTfF
B.G.OGT        GARNELFALRPAPIQAMWLGYPGTSGAlFMDYIITDQETSPAEVAEQYSEKLAYmPHTfF
H.G.OGT1       GARNELFALRPAPIQAMWLGYPGTSGAlFMDYIITDQETSPAEVAEQYSEKLAYmPHTfF
S.G.OGT        GARNELFALRPAPIQAMWLGYPGTSGAlFMDYIITDQETSPAEVAEQYSEKLAYmPHTfF
eG1            GARNELFALRPAPIQAMWLGYPGTSGAlFMDYIITDQETSPAEVAEQYSEKLAYmPHTfF
e.c.GT41_O     GARNELFALRPAPIQAMWLGYPGTSGAlFMDYIITDQETSPAEVAEQYSEKLAYmPHTfF
Da.re.GT41     GARNELFALRPAPIQAMWLGYPGTSGApFMDYIvsDKaTSPiEVAEQYSEKLAYmPnTfF
te.n.GT41      GARNELFALRPAPvQAMWLGYPGTSGApFMDYIIsDKETSPiEVAEQYSEKLAYmPHTfF
Dm.G.OGT       GARNEiFALRPAPIQvMWLGYPGTSGAsFMDYIITDsvTSPlElAyQYSEKLsYmPHTyF
a.g.GT41_O     GARNEiFALRPAPvQvMWLGYPGTSGAsFMDYIvTDavTSPlslesQYSEKLAYmPHTyF
Ae.a.GT41      GARNEiFALRPAPIQvMWLGYPGTSGAsFMDYIITDtvTSPmElAdQYSEKLAYmPHTyF
```

```
bG1           GARNEiFALRPAPvQvMWLGYPGTSGAsyMDYlvTDavTSPvElAsQYSEKLAYmPHTyF
Ap.me.GT41    GARNEiFALRPAPvQvMWLGYPGTSGAsFMDYlITDEvTSPlElAsQYSEKLAYmPHTyF
ApG2          GARNEiFALRPAPvQvMWLGYPGTSGAsFMDYlITDEvTSPlElAsQYSEKLAYmPHTyF
Da.re.AGO6    ----------------------------------------------------AqmitSMF
te.n.AGO61    ----------------------------------------------------AqLitaLF
ta.ru.AGO6    ----------------------------------------------------AqLitSLF
M.AGO61       ----------------------------------------------------AqLvtaLF
R.AGO61       ----------------------------------------------------AqLvtaLF
H.AGO61       ----------------------------------------------------AqLvtTLF
e.c.AGO61     ----------------------------------------------------AqLvtaLF
S.AGO61       ----------------------------------------------------AqLvtaLF
B.AGO61       ----------------------------------------------------AqLvtaLF
G.AGO611      ----------------------------------------------------AqLvmSLF
x.t.AGO61     ----------------------------------------------------AqLitSLF
X.l.AGO61     ----------------------------------------------------AqLvtSLF
ta.ru.eOGT    ----------------------------------------------------AgLtHlLF
te.n.eOGT     ----------------------------------------------------AgLtHlLF
X.l.eOGT      ----------------------------------------------------AgLtHlLF
x.t.eOGT      ----------------------------------------------------AgLtHlLF
G.eOGT        ----------------------------------------------------AgLtHlLF
M.eOGT        ----------------------------------------------------AgLtHlLF
R.eOGT        ----------------------------------------------------AgLtHlLF
S.eOGT        ----------------------------------------------------AgLtHlLF
B.eOGT        ----------------------------------------------------AgLtHlLF
H.eOGT        ----------------------------------------------------AgLtHlLF
e.c.eOGT      ----------------------------------------------------AgLtHlLF
Dm.eOGT       ----------------------------------------------------AgLtHlLF
a.g.eOGT      ----------------------------------------------------AgLtHlLF
Ae.a.eOGT     ----------------------------------------------------AgLtHlLF
Ap.me.eOGT    ----------------------------------------------------AgLtHlMF
b.mo.eOGT     ----------------------------------------------------AgLtHlLF
```

```
AeO2         rPqeAaI----------------------sYkealrLqpnDLttl---------------
Ae.a.OGTli   ------------------------------------------------------------
b.mo.GT41    vgDHkkmFphLKtKykvlid-----nNsiahENvAliNssefini---------------
H.G.OGT2     igDHAnmFphLKKKAviDfK-----SNGHiYDNrivLNGIDLKaFLDSLPDVKIVKMKCP
X.l.GT41_O   igDHAnmFphLKKKAviDfK-----SNGHiYDNrivLNGIDLKaFLeSLPDVKIVKMKgP
x.t.GT41_O   igDHAnmFphLKKKAviDfK-----SNGHiYDNrivLNGIDLKaFLeSLPDVKIVKMKgP
G.G.OGT      igDHAnmFphLKKKAviDfK-----SNGHiYDNrivLNGIDLKaFLDSLPDVKIVKMKCP
M.G.OGT      igDHAnmFphLKKKAviDfK-----SNGHiYDNrivLNGIDLKaFLDSLPDVKIVKMKCP
B.G.OGT      igDHAnmFphLKKKAviDfK-----SNGHiYDNrivLNGIDLKaFLDSLPDVKIVKMKCP
H.G.OGT1     igDHAnmFphLKKKAviDfK-----SNGHiYDNrivLNGIDLKaFLDSLPDVKIVKMKCP
S.G.OGT      igDHAnmFphLKKKAviDfK-----SNGHiYDNrivLNGIDLKaFLDSLPDVKIVKMKCP
eG1          igDHAnmFphLKKKAviDfK-----SNGHiYDNrivLNGIDLKaFLDSLPDVKIVKMKCP
e.c.GT41_O   igDHAnmFphLKKKAviDfK-----SNGHiYDNrivLNGIDLKaFLDSLPDVKIVKMKCP
Da.re.GT41   igDHAnmFphLKKKAviDfK-----SNGHifDNrivLNGIDLKaFLeSLPDVKVVKMeC-
te.n.GT41    igDHAnmFphLKKKAviDfK-----SNGHifDNrivLNGIDLKaFLDSLPDVKVIKMKCd
Dm.G.OGT     igDHkqmFphLKERiivcDK-----qqssvvDNvtviNatDLsplven-tDVKeIKevv-
a.g.GT41_O   igDHrqmFphLKERvivsNR-----aNnslaDNvAviNatDLsplveS-tDVKtVrevv-
Ae.a.GT41    igDHrqmFphLKERlivsgK----qqNnqlvDNvAviNatDLsplven-tDVKtVrevi-
bG1          vgDHkqmFphLqERlilsDKvkshNnlGsvaDNvAviNatDLsplven-tDIKeIKeiv-
Ap.me.GT41   igDHkqmFphLKERliltDK---lNmkGkvaDNvAviNatDLspmientcvkeIrevvvP
ApG2         igDHkqmFphLKERliltDK---lNmkGkvaDNvAviNatDLspmientcvkeIrevvvP
Da.re.AGO6   LPrgAaVvELfpygvNpEqy--------tpYktLAsLpGmDLQYv---------------
te.n.AGO61   LPpgAvVvELfpfavNpDqy--------tpYrtLAaLpGmDLhYi---------------
ta.ru.AGO6   LPpgAvVvELypfavNpDqy--------tpYrtLAsLpGmDLhYi---------------
M.AGO61      LPrgAtVvELfpyavNpDhy--------tpYktLAtLpGmDLQYv---------------
R.AGO61      LPrgAtVvELfpyavNpDhy--------tpYktLAtLpGmDLQYv---------------
H.AGO61      LPrgAtVvELfpyavNpDhy--------tpYktLAmLpGmDLQYv---------------
e.c.AGO61    LPrgAtVvELfpyavNpDhy--------tpYktLAmLpGmDLQYv---------------
S.AGO61      LPrgAaVvELfpyavNpDhy--------tpYktLAtLpGmDLQYi---------------
B.AGO61      LPrgAaVvELfpyavNpDhy--------tpYktLAtLpGmDLQYi---------------
G.AGO611     LPrgAtVvELfpyaiNpEhy--------tpYktLAtLpGmDLQYi---------------
x.t.AGO61    LPkgAiVvELfpygvNpEhy--------tpYktLstLpGmeLQYv---------------
```

```
X.l.AGO61      LPkgAvVvELfpygiNpEhy--------tpYktLstLpGmeLQYv---------------

ta.ru.eOGT     LPDwAvIFELy----NCqDE--------sCYrdLArLrG--vRYv---------------

te.n.eOGT      LPDwAvIFELy----NCqDE--------sCYrdLArLrG--vRYv---------------

X.l.eOGT       LPDwAvVFELy----NCEDa--------rCYldLArLrG--iQYm---------------

x.t.eOGT       LPDwAvVFELy----NCEDE--------rCYldLArLrG--iRYm---------------

G.eOGT         LPDwAvVFELy----NCEDE--------rCYldLArLrG--ihYi---------------

M.eOGT         LPDwAaVFELy----NCEDE--------rCYldLArLrG--ihYi---------------

R.eOGT         LPDwAaVFELy----NCEDE--------rCYldLArLrG--iyYi---------------

S.eOGT         LPDwAaVFELy----NCgDE--------rCYldLArLrG--vhYi---------------

B.eOGT         LPDwAaVFELy----NCgDE--------rCYldLArLrG--vhYi---------------

H.eOGT         LPDwAaVFELy----NCEDE--------rCYldLArLrG--vhYi---------------

e.c.eOGT       LPDwAaVFELy----NCEDE--------rCYldLArLrG--vhYi---------------

Dm.eOGT        LPnwAcIFELy----NCEDp--------NCYkdLArLrG--vRYr---------------

a.g.eOGT       LPkwAtlFELy----HCEDp--------NCYrdLArLrG--vhYl---------------

Ae.a.eOGT      LPkwAvlFELy----HCEDp--------NCYkdLArLkG--vRYl---------------

Ap.me.eOGT     LPDwAaVFEIy----NCEDp--------gCYkdLArLrG--vKYF---------------

b.mo.eOGT      LPDwAaVFEIy----NCEDp--------NCYadLsrLrG--LKYv---------------


AeO2           ------------------------------------------------------------

Ae.a.OGTli     ------------------------------------------------------------

b.mo.GT41      ---------------devfnvrcdktiVsfeNleeiEfiIrkVnIPNyVldttiNpkqe-

H.G.OGT2       DGGDNaDsSNTALNMPVIPMNTIAEAVIEMINrgQiQITINGfsIsNGLATtQiNNkAAt

X.l.GT41_O     DsGDNVD-NNSALsMPVIPMssIAEAVIEMINrgQiQITINGfnIsNGLATtQiNNkAAt

x.t.GT41_O     DsGDNVD-NNSALNMPVIPMssIAEAVIEMINrgQiQITINGfnIsNGLATtQiNNkAAt

G.G.OGT        DsGDsaD-SNaALsMPVIPMNTIAEAVIEMINrgQiQITINGfnIsNGLATtQiNNkAAt

M.G.OGT        DGGDNaDtTNTALNMPVIPMNTIAEAVIEMINrgQiQITINGfsIsNGLATtQiNNkAAt

B.G.OGT        DGGDNVDsSNTALNMPVIPMNTIAEAVIEMINrgQiQITINGfsIsNGLATtQiNNkAAt

H.G.OGT1       DGGDNaDsSNTALNMPVIPMNTIAEAVIEMINrgQiQITINGfsIsNGLATtQiNNkAAt

S.G.OGT        DGGDNaDsSNTALNMPVIPMNTIAEAVIEMINrgQiQITINGfsIsNGLATtQiNNkAAt

eG1            DGGDNaDsSNTALNMPVIPMNTIAEAVIEMINrgQiQITINGfsIsNGLATtQiNNkAAt

e.c.GT41_O     DGGDNaDsSNTALNMPVIPMNTIAEAVIEMINrgQiQITINGfsIsNGLATtQiNNkAAt

Da.re.GT41     -dGqeVadSNgALsMPIIPMNTaAEAiInMINqgQiQVTINGfTVsNGLATtQiNNkAAt
```

```
te.n.GT41      nnqEpsadTNgALsMPVIPMNTaAEAiInMINqgQiQVTINGfTVsNGLATtQitNkAAt
Dm.G.OGT       naqkpVEiThkvaelP------nttqiVsMIaTgQvQtSLNGVvVqNGLATtQtNNkAAt
a.g.GT41_O     lankpVEiqhkvaelP------tttpietMIaSgQvQtSLNGVvVqNGLATtQtNNkAAt
Ae.a.GT41      lahkpVEiqhkvvelP------tttpVetMIaSgQiQtSLNGVvVqNGLATtQtNNkAAt
bG1            raarpVEiSlkvaelP------tttpietMIaSgQvQtSVNGViLqNGLATtQtNNkAAt
Ap.me.GT41     Daknkpveislk-----vaelptttpietMIaSgQcQMSVNGVvVqNGMATtQvNNktAt
ApG2           Daknkpveislk-----vaelptttpietMIaSgQcQMSVNGVvVqNGMATtQvNNktAt
Da.re.AGO6     --------------------------------aWrntMeeNtVTfPdrpwdQggivHlek
te.n.AGO61     --------------------------------SWrnteeeNtIThPdrpweQggiaHlek
ta.ru.AGO6     --------------------------------pWrnteeeNtVThPdrpweQggiaHlek
M.AGO61        --------------------------------aWrnmIreNtVThPerpwdQggitHldr
R.AGO61        --------------------------------aWrnmIreNtVThPerpwdQggitHldr
H.AGO61        --------------------------------aWrnmMpeNtVThPerpwdQggitHldr
e.c.AGO61      --------------------------------aWrnmMpeNtVThPerpwdQggitHldq
S.AGO61        --------------------------------aWrntMpeNtVThPerpwdQggiaHldr
B.AGO61        --------------------------------aWQntMpeNtVThPerpwdQggiaHldr
G.AGO611       --------------------------------aWQntaredtVTyPdrpwdQggiaHldk
x.t.AGO61      --------------------------------aWQnteeeNtIayPNrpweQggivHldk
X.l.AGO61      --------------------------------aWQnteeeNtITyPdrpweQggivHlet
ta.ru.eOGT     --------------------------------TWQq---------mNkVfpQdkghHptl
te.n.eOGT      --------------------------------TWQk---------mNqVfpQdkghHptl
X.l.eOGT       --------------------------------TWek---------gdkVfpQdkghHpnl
x.t.eOGT       --------------------------------TWen---------rdkVfpQdkghHpnl
G.eOGT         --------------------------------TWrk---------rNkVfpQdqghHptl
M.eOGT         --------------------------------TWrk---------PskVfpQdkghHptl
R.eOGT         --------------------------------TWQk---------PskVfpQdkghHptl
S.eOGT         --------------------------------TWrr---------qNkVfpQdkghHptl
B.eOGT         --------------------------------TWrr---------qNkVfpQdkghHptl
H.eOGT         --------------------------------TWrr---------qNkVfpQdkghHptl
e.c.eOGT       --------------------------------TWrr---------qNkVfpQdkghHptl
Dm.eOGT        --------------------------------TWeq---------rdlVypQdeghHpeg
a.g.eOGT       --------------------------------TWer---------dhlVypEgegkHper
```

Ae.a.eOGT	--------------------------------SWer---------ddlVypEdeghHpdg
Ap.me.eOGT	--------------------------------TWen---------nskLvqQdpgtHpdg
b.mo.eOGT	--------------------------------TWed---------kskLvqQdeghspgg
AeO2	----------------------------tnlaKLgiaevs--------------------
Ae.a.OGTli	--
b.mo.GT41	-----------------------------klYKIDPrimesWvtILKlVPNSVLWLLsfP
H.G.OGT2	GEEVPRtIIVTTRsQYGLPEDAiVYCNFNQlYKIDPsTLQMWANILKRVPNSVLWLLRfP
X.l.GT41_O	GEEVPRtIIVTTRsQYGLPEDAvVYCNFNQlYKIDPsTLQMWANILKRVPNSVLWLLRfP
x.t.GT41_O	GEEVPRtIIVTTRsQYGLPEDAvVYCNFNQlYKIDPsTLQMWANILKRVPNSVLWLLRfP
G.G.OGT	GEEVPRtIIVTTRsQYGLPEDAvVYCNFNQlYKIDPsTLQMWANILKRVPNSVLWLLRfP
M.G.OGT	GEEVPRtIIVTTRsQYGLPEDAiVYCNFNQlYKIDPsTLQMgANILKRVPNSVLWLLRfP
B.G.OGT	GEEVPRtIIVTTRsQYGLPEDAiVYCNFNQlYKIDPsTLQMWANILKRVPNSVLWLLRfP
H.G.OGT1	GEEVPRtIIVTTRsQYGLPEDAiVYCNFNQlYKIDPsTLQMWANILKRVPNSVLWLLRfP
S.G.OGT	GEEVPRtIIVTTRsQYGLPEDAiVYCNFNQlYKIDPsTLQMWANILKRVPNSVLWLLRfP
eG1	GEEVPRtIIVTTRsQYGLPEDAiVYCNFNQlYKIDPsTLQMWANILKRVPNSVLWLLRfP
e.c.GT41_O	GEEVPRtIIVTTRsQYGLPEDAiVYCNFNQlYKIDPsTLQMWANILKRVPNSVLWLLRfP
Da.re.GT41	GEEVPRtIVVTTRsQYGLPEDsiVYCNFNQlYKIDPpTLQMWANILKRVPNSViWLLRfP
te.n.GT41	GEEVlRtVVVTTRsQYGLPEDAiVYCNFNQlYKIDPpTLQMWANILtRVPNSVLWLLRfP
Dm.G.OGT	GEEVPqnIVITTRrQYmLPDDAvVYCNFNQlYKIDPqTLesWveILKnVPkSVLWLLRfP
a.g.GT41_O	GEEVPqnIVVTTRqQYGLPDDAiVYCNFNQlYKIDPlTLQsWvtILKhVPNSVLWLLRfP
Ae.a.GT41	GEEVPqnIVVTTRqQYGLPDDAvVYCNFNQlYKIDPhTLasWvNILKhVPNSVLWLLRfP
bG1	GEEVPqsIVITTRqQYGLPDDAvVYCNFNQlYKIDPlTLhMWvyILKhVPNSVLWLLRfP
Ap.me.GT41	GEEVPqnIMITTRqQYGLPEDAvVYCNFNQlYKIDPlTLhMWAhILKhVPNSVLWLLRfP
ApG2	GEEVPqnIMITTRqQYGLPEDAvVYCNFNQlYKIDPlTLhMWAhILKhVPNSVLWLLRfP
Da.re.AGO6	eE-qeRIlaskevPrhlccrnpEwlfriyQdttVDlasf--------------LdvLRdg
te.n.AGO61	eE-qeRIVaskdvPrhlccrnpEwlfriyQdtfVDipsf--------------LeaLqag
ta.ru.AGO6	eE-qeqImaskdvPrhlccrnpEwlfriyQdtlVDipsf--------------LevLqeg
M.AGO61	aE-qaRIlqsrevPrhlccrnpEwlfriyQdtRVDipsL--------------mqsiRrv
R.AGO61	aE-qaRIlqsrevPrhlccrnpEwlfriyQdtRVDipsL--------------mqsiRrv
H.AGO61	aE-qaRIlqsrevPrhlccrnpEwlfriyQdtKVDipsL--------------iqtiRrv
e.c.AGO61	aE-qaRIlqsrevPrhlccrnpEwlfriyQdtKVDipsL--------------iqsiRrv

```
S.AGO61        aE-qaRIlqsqevPrhlccrnpEwlfriyQdtKVDipsL--------------iqtiRrv
B.AGO61        aE-qaRIlqsrevPrhlccrnpEwlfriyQdtKVDipsL--------------iqtiRrv
G.AGO611       aE-qeRIIksTevPrhlccrnpEwlfrayQdtKVDipsL--------------ihviRqt
x.t.AGO61      tE-qeRIkkskevPrhlccrnpEwlfriyQdtKVnissL--------------iqviKsk
X.l.AGO61      kE-qeRIkkskevPrhlccrnpEwlfriyQdtKVnissL--------------iqviKst
ta.ru.eOGT     Gd-hPk----------------------ftnYsfDvaef--------------mrIvlea
te.n.eOGT      Gd-hPk----------------------ftnYsfDvaef--------------vrLvlea
X.l.eOGT       GE-hPk----------------------ftnYafDveef--------------LrLvqqg
x.t.eOGT       GE-hPk----------------------ftnYafDveef--------------LrLvRqa
G.eOGT         GE-hPk----------------------ftnYsfDveef--------------mYLvlla
M.eOGT         GE-hPk----------------------ftnYsfDveef--------------mYLvlqa
R.eOGT         GE-hPk----------------------ftnYsfDveef--------------mYLvlqa
S.eOGT         GE-hPk----------------------ftnYsfDveef--------------mfLvlqa
B.eOGT         GE-hPk----------------------ftnYsfDveef--------------mYLvlqa
H.eOGT         GE-hPk----------------------ftnYsfDveef--------------mYLvlqa
e.c.eOGT       GE-hPk----------------------ftnYsfDveef--------------mYLvlqa
Dm.eOGT        Ga-hak----------------------ftnYsfDvkef--------------vhLvdga
a.g.eOGT       dErhak----------------------ftnYafDvgef--------------erLvaka
Ae.a.eOGT      Ggrhak----------------------ftnYafDakef--------------arLvaig
Ap.me.eOGT     Ga-hak----------------------ftnYsfDveef--------------LrIvsqa
b.mo.eOGT      Gs-hak----------------------ftnYsfDvkef--------------LrLvakc

AeO2           ------------------------------------------------------------
Ae.a.OGTli     ------------------------------------------------------------
b.mo.GT41      AaGEPNIQkYglNLGkdsqtecLtPGRIIFSkiAcKEEHVRRGQLADICLDTPLCNGHTT
H.G.OGT2       AvGEPNIQQYAQNMG-------LPqnRIIFSPVAPKEEHVRRGQLADVCLDTPLCNGHTT
X.l.GT41_O     AvGEPNIQQYAQNMG-------LPqsRIIFSPVAPKEEHVRRGQLADVCLDTPLCNGHTT
x.t.GT41_O     AvGEPNIQQYAQNMG-------LPqsRIIFSPVAPKEEHVRRGQLADVCLDTPLCNGHTT
G.G.OGT        AvGEPNIQQYAQNLG-------LsqnRIIFSPVAPKEEHVRRGQLADVCLDTPLCNGHTT
M.G.OGT        AvGEPNIQQYAQNMG-------LPqnRIIFSPVAPKEEHVRRGQLADVCLDTPLCNGHTT
B.G.OGT        AvGEPNIQQYAQNMG-------LPqnRIIFSPVAPKEEHVRRGQLADVCLDTPLCNGHTT
H.G.OGT1       AvGEPNIQQYAQNMG-------LPqnRIIFSPVAPKEEHVRRGQLADVCLDTPLCNGHTT
```

```
S.G.OGT       AvGEPNIQQYAQNMG-------LPqnRIIFSPVAPKEEHVRRGQLADVCLDTPLCNGHTT
eG1           AvGEPNIQQYAQNMG-------LPqnRIIFSPVAPKEEHVRRGQLADVCLDTPLCNGHTT
e.c.GT41_O    AvGEPNIQQYAQNMG-------LPqnRIIFSPVAPKEEHVRRGQLADVCLDTPLCNGHTT
Da.re.GT41    AvGEPNIQQYAQNLG-------LPasRIIFSPVAPKEEHVRRGQLADVCLDTPLCNGHTT
te.n.GT41     AvGEPNIQQYAQkVG-------LPasRIIFSPVAPKEEHVRRGQLADVCLDTPLCNGHTT
Dm.G.OGT      AvGEqNIkktvsdfG-------isPdRvIFSnVAaKEEHVRRGQLADICLDTPLCNGHTT
a.g.GT41_O    AvGEaNIQatAQqMG-------iaaGRIIFSnVAaKEEHVRRGQLADVCLDTPLCNGHTT
Ae.a.GT41     AvGEtNIQaaAQqLG-------LsPGRIIFSnVAaKEEHVRRGQLADVCLDTPLCNGHTT
bG1           AvGEPNLQstAnqLG-------LPPGRIIFSnVAaKEEHVRRGQLADVCLDTPLCNGHTT
Ap.me.GT41    AvGEPNLQatAQqLG-------LaPGRIlFSnVAaKEEHVRRGQLADVCLDTPLCNGHTT
ApG2          AvGEPNLQatAQqLG-------LaPGRIlFSnVAaKEEHVRRGQLADVCLDTPLCNGHTT
Da.re.AGO6    lk-klNLkkakvast-------vhPGR-------------vRepkcqtsvqatneaklsv
te.n.AGO61    lkakPvwkksklsgG-------LhPGR-------------vRdarcqtsvqTsseaklTv
ta.ru.AGO6    vkakPlLkksklsst-------LhPGR-------------vRdpqcqtsvqTsneaklTv
M.AGO61       vkGrPgprrqrwaIs-------LyPGk-------------vRearcqasvqgatearlsv
R.AGO61       vkGrPgprrqrwaIs-------LyPGk-------------vRearcqasvqgatearlsv
H.AGO61       vkGrPgprkqkwtVG-------LyPGk-------------vRearcqasvhgasearlTv
e.c.AGO61     vkGrPgprkqkwtVG-------LyPGk-------------vRearcqaavqgasearlTv
S.AGO61       vkGrPgprkqkwtVs-------LyPGk-------------vRearcqssvqgasearlTv
B.AGO61       vkGhPgprkqkwtVs-------LyPGk-------------vRearcqasvqgasearlsv
G.AGO611      vkskPgpkk-kwsgs-------LyPGk-------------vRdarcqasvqgtsearlsv
x.t.AGO61     tk--lgsrrqkwtqG-------LyPGk-------------vReskcqasaqgtgeaklfv
X.l.AGO61     vkkklgLrrqkwtqG-------LyPGk-------------vReskcqasaqgtseaklfv
ta.ru.eOGT    Ad----------------------------------------------------------
te.n.eOGT     Ad----------------------------------------------------------
X.l.eOGT      At----------------------------------------------------------
x.t.eOGT      Ak----------------------------------------------------------
G.eOGT        An----------------------------------------------------------
M.eOGT        Ae----------------------------------------------------------
R.eOGT        Ae----------------------------------------------------------
S.eOGT        Ad----------------------------------------------------------
B.eOGT        Ad----------------------------------------------------------
```

```
H.eOGT          Ad----------------------------------------------------------
e.c.eOGT        Ad----------------------------------------------------------
Dm.eOGT         Ae----------------------------------------------------------
a.g.eOGT        AayvqghaEFq-------------------------------------------------
Ae.a.eOGT       Ae----------------------------------------------------------
Ap.me.eOGT      tdyvkNhdsF--------------------------------------------------
b.mo.eOGT       Ae----------------------------------------------------------

AeO2            ------------------------------------------------------------
Ae.a.OGTli      ------------------------------------------------------------
b.mo.GT41       tMDiLWtGTPvVTlP------------------GdTLASRVAASQLtaLhCtELIAknRK
H.G.OGT2        gMDVLWAGTPmVTMP------------------GETLASRVAASQLtCLGClELIAknRQ
X.l.GT41_O      gMDVLWAGTPmVTMPgkesaeslglhnkdcflsGdTLASRVAASQLtCLGCPELIAksRQ
x.t.GT41_O      gMDVLWAGTPmVTMP------------------GdTLASRVAASQLtCLGCPELIAkgRQ
G.G.OGT         gMDVLWAGTPmVTMP------------------GETLASRVAASQLtCLGClELIAksRQ
M.G.OGT         gMDVLWAGTPmVTMP------------------GETLASRVAASQLtCLGClELIAksRQ
B.G.OGT         gMDVLWAGTPmVTMP------------------GETLASRVAASQLtCLGClELIAknRQ
H.G.OGT1        gMDVLWAGTPmVTMP------------------GETLASRVAASQLtCLGClELIAknRQ
S.G.OGT         gMDVLWAGTPmVTMP------------------GETLASRVAASQLtCLGClELIAknRQ
eG1             gMDVLWAGTPmVTMP------------------GETLASRVAASQLtCLGClELIAknRQ
e.c.GT41_O      gMDVLWAGTPmVTMP------------------GETLASRVAASQLtCLGClELIAknRQ
Da.re.GT41      gMDVLWAGTPmVTMP------------------GETLASRVAASQLtCLGCPELIAqsRQ
te.n.GT41       gMDVLWAGTPmVTMP------------------GETLASRVAtSQLsCLGCPELIAqshE
Dm.G.OGT        SMDVLWtGTPvVTlP------------------GETLASRVAASQLatLGCPELIArtRE
a.g.GT41_O      SMDVLWtGTPvVTlP------------------aETLASRVAASQLatLGCPELIAksRQ
Ae.a.GT41       SMDVLWtGTPvVTlP------------------aETLASRVAASQLatLGCPELIArsRQ
bG1             SMDiLWtGTPvVTlP------------------GETLASRVAASQLntLGCPELIArtRQ
Ap.me.GT41      SMDVLWtGTPvVTlP------------------GETLASRVAASQLntLGCPELIArtRQ
ApG2            SMDVLWtGTPvVTlP------------------GETLASRVAASQLntLGCPELIArtRQ
Da.re.AGO6      SwqipWnlkyLkv--------------------kEvkyevwiqeQgEntymPyiLpHQny
te.n.AGO61      SwqmpWnlkyLkv--------------------rEvkyevwiqeQgEntymPyiLpqQny
ta.ru.AGO6      SwqipWnlkyLkv--------------------rEvkyevwiqeQgEntymPyiLpqQny
```

```
M.AGO61      SwqipWnlkyLkv--------------------rEvkyevwlqeQgEntyvPymLtlQnh
R.AGO61      SwqipWnlkyLkv--------------------rEvryevwlqeQgEntyvPymLtlQnh
H.AGO61      SwqipWnlkyLkv--------------------rEvkyevwlqeQgEntyvPyiLAlQnh
e.c.AGO61    SwqipWnlkyLkv--------------------rEvkyevwlqeQgEntyvPymLAlQnh
S.AGO61      SwqipWnlkyLkv--------------------rEvkyevwlqeQgEntyvPyiLtlQnh
B.AGO61      SwqipWnlkyLkv--------------------rEvkyevwlqeQgEntyvPymLAlQnh
G.AGO611     SwqVpWnlkyLkv--------------------rEvkyevwiqeQgEntymPyiLSHQnh
x.t.AGO61    SwqipWnlkfLkv--------------------rdvkyevwiqeQgEnsymPyiLSqQny
X.l.AGO61    SwqipWnlkfLkv--------------------rdvkyevwiqeQgEnsymPyiLSqQny
ta.ru.eOGT   ----------------------------------------------------yvtrHpKw
te.n.eOGT    ----------------------------------------------------yvtrHpew
X.l.eOGT     ----------------------------------------------------yvsrHsKw
x.t.eOGT     ----------------------------------------------------nvsrHsKw
G.eOGT       ----------------------------------------------------hvsqHsKw
M.eOGT       ----------------------------------------------------hvLqHpqw
R.eOGT       ----------------------------------------------------hvLqHpqw
S.eOGT       ----------------------------------------------------hvLqHpKw
B.eOGT       ----------------------------------------------------yvLqHpKw
H.eOGT       ----------------------------------------------------hvLqHpKw
e.c.eOGT     ----------------------------------------------------yvLqHpKw
Dm.eOGT      ----------------------------------------------------EiLSHKef
a.g.eOGT     ----------------------------------------------------kfLttnan
Ae.a.eOGT    ----------------------------------------------------hvwnHEey
Ap.me.eOGT   ---------------------------------knfvAkKIqhkgtEmknqtnvMSdvKE
b.mo.eOGT    ----------------------------------------------------yvrnrQdf

AeO2         ------------------------------------------------------------
Ae.a.OGTli   ------------------------------------------------------------
b.mo.GT41    hYEDiaiKLGTDsaYrryIRaKVsKaRLeStLFdcehYargLEsLYskMWElYqrGdKPD
H.G.OGT2     EYEDiavKLGTDLEYLKkVRgKVWKqRISSPLFNTKQYTMELERLYlQMWEHYAAGNKPD
X.l.GT41_O   DYEDiavKLGTDLEYLKkIRaKVWKqRISSPLFNTKQYTMDLERLYlEMWEHfAAGNKrD
x.t.GT41_O   DYEDtavKLGTDLEYLKkIRsKVWKqRISSPLFNTKQYTiDLERLYlQMWEHYsAGNKtD
```

```
G.G.OGT       EYEDiavKLGTDLEYLKkIRgKVWKqRISSPLFNTKQYTMDLERLYlQMWdHYAAGNKPD
M.G.OGT       EYEDiavKLGTDLEYLKkIRgKVWKqRISSPLFNTKQYTMELERLYlQMWEHYAAGNKPD
B.G.OGT       EYEDiavKLGTDLEYLKkIRgKVWKqRISSPLFNTKQYTMELERLYlQMWEHYAAGNKPD
H.G.OGT1      EYEDiavKLGTDLEYLKkVRgKVWKqRISSPLFNTKQYTMELERLYlQMWEHYAAGNKPD
S.G.OGT       EFEDiavKLGTDLEYLKkIRgKVWKqRISSPLFNTKQYTMELERLYlQMWEHYAAGNKPD
eG1           EYEDiavKLGTDLEYLKkIRgKVWKqRISSPLFNTKQYTMELERLYlQMWEHYAAGNKPD
e.c.GT41_O    EYEDiavKLGTDLEYLKkIRgKVWKqRISSPLFNTKQYTMELERLYlQMWEHYAAGNKPD
Da.re.GT41    EYEDvavKLGTDMEfLKkVRarVWKqRIcSPLFNTKQYTMDLEKLYlQMWEnhAsGgKPD
te.n.GT41     EYEDiavKLGsDMEYLKmVRarVWKqRIcSPLFNTKQYTMDLEKLYlrMWEHYskGNKPe
Dm.G.OGT      EYQniaiRLGTkkEYLKalRaKVWKaRVeSPLFdcsQYakgLEKLflrMWEkYenGelPD
a.g.GT41_O    EYQDiaiKLGTDrEYLKamRaKVWvaRceSPLFdcKQYaqgLEmLfykMWErfArGerPD
Ae.a.GT41     EYQDiaiKLGTDkEYLKaIRaKVWlaRceSPLFdcKQYaqgmEaLfykMWErfArGeKPD
bG1           EYQDiavRLGTDrEYLKaIRaKVWtaRtdSPLFdcKaYatgLEmLYnrMWsrhArGdrPD
Ap.me.GT41    EYQDiaiRLGTDrEYLKatRaKVWKaRseSPLFNcKlYaMgmEmLYkkMWErYArGeKPD
ApG2          EYQDiaiRLGTDrEYLKatRaKVWKaRseSPLFNcKlYaMgmEmLYkkMWErYArGeKPD
Da.re.AGO6    tFsEnikpftTyLvwvrCIfNKnllG------------------pfadv-----------
te.n.AGO61    tFsDnikpftTyLvwvrCIfNKnllG------------------pfadv-----------
ta.ru.AGO6    tFsDnikpftTyLvwvrCIfNKnllG------------------pfadv-----------
M.AGO61       tFtEnikpftTyLvwvrCIfNrsllG----PfcrcagvqhvasRpwpglWraplAGsafp
R.AGO61       tFtEnikpftTyLvwvrCIfNrsllG------------------pfadv-----------
H.AGO61       tFtEnikpftTyLvwvrCIfNKIllG------------------pfadv-----------
e.c.AGO61     tFtEnikpftTyLvwvrCIfNKtllG------------------pfadv-----------
S.AGO61       tFtEnikpftTyLvwvrCIfNKtllG------------------pfadv-----------
B.AGO61       tFtEnikpftTyLvwirCIfNKtllG------------------pfadv-----------
G.AGO611      tFsEnikpftiyLvwirCIfNKnllG------------------pfadv-----------
x.t.AGO61     tFsEnikpLtTyLvwirCIfNKtllG------------------pfaEv-----------
X.l.AGO61     tFsEnikpftTyLvwirCIfNKtllG------------------pfaEv-----------
ta.ru.eOGT    QrrtprdeL---------------------------------------------------
te.n.eOGT     rrQtprdeL---------------------------------------------------
X.l.eOGT      plrrtrdeL---------------------------------------------------
x.t.eOGT      pFrrtrdeL---------------------------------------------------
G.eOGT        pFrvkhdef---------------------------------------------------
```

```
M.eOGT        pFKkkhdeL---------------------------------------------------
R.eOGT        plKknhdeL---------------------------------------------------
S.eOGT        pFKnkhdeL---------------------------------------------------
B.eOGT        pFKkkhdeL---------------------------------------------------
H.eOGT        pFKkkhdeL---------------------------------------------------
e.c.eOGT      pFKkkrdeL---------------------------------------------------
Dm.eOGT       prrasenpskTqrneL--------------------------------------------
a.g.eOGT      ravkrkdeL---------------------------------------------------
Ae.a.eOGT     QqflersRrkqEkls-----------------------------gkdEl-----------
Ap.me.eOGT    satskskevtelksdtrskdel--------------------------------------
b.mo.eOGT     QnfveaslIk----------------------------------MheEl-----------

AeO2          ------------------
Ae.a.OGTli    ------------------
b.mo.GT41     Hiavssk-----------
H.G.OGT2      HMI--KPvEvTESA----
X.l.GT41_O    HLI--KPvEsTESA----
x.t.GT41_O    HLI--KtvEsTESA----
G.G.OGT       HMI--KPvEaSESA----
M.G.OGT       HMI--KPvEvTESA----
B.G.OGT       HMI--KPvEvTESA----
H.G.OGT1      HMI--KPvEvTESA----
S.G.OGT       HMI--KPvEvTESA----
eG1           HMI--KPvEvTESA----
e.c.GT41_O    HMI--KPvEvTESA----
Da.re.GT41    HLVkmqsLEtSESt----
te.n.GT41     HLV--qtvEaSEnA----
Dm.G.OGT      Hi---sav----------
a.g.GT41_O    Hi---sakDk--------
Ae.a.GT41     Hi---sakEgpk------
bG1           Hi---qaLEk--------
Ap.me.GT41    HvsavdknEreklITAAS
```

```
ApG2            HvsavdknEreklllTAAS
Da.re.AGO6      --------LiCkt------
te.n.AGO61      --------LmCrt------
ta.ru.AGO6      --------LmCrt------
M.AGO61         gpqfc--------------
R.AGO61         --------LvCSt------
H.AGO61         --------LvCnt------
e.c.AGO61       --------LvCnt------
S.AGO61         --------LvCnt------
B.AGO61         --------LvCSt------
G.AGO611        --------LlCSt------
x.t.AGO61       --------LvCnt------
X.l.AGO61       --------LvCSt------
ta.ru.eOGT      -------------------
te.n.eOGT       -------------------
X.l.eOGT        -------------------
x.t.eOGT        -------------------
G.eOGT          -------------------
M.eOGT          -------------------
R.eOGT          -------------------
S.eOGT          -------------------
B.eOGT          -------------------
H.eOGT          -------------------
e.c.eOGT        -------------------
Dm.eOGT         -------------------
a.g.eOGT        -------------------
Ae.a.eOGT       -------------------
Ap.me.eOGT      -------------------
b.mo.eOGT       -------------------
```
